# Lymphatic dysfunction and ZFP36 deficiency contribute to myxomatous valve degeneration in Marfan syndrome mice

**DOI:** 10.1172/JCI195507

**Published:** 2026-06-02

**Authors:** Can Tan, Ziyou Ren, Shreya Kurup, Xianpeng Liu, Zhi-Dong Ge, Shodai Suzuki, Pritika Jakka, Cheryl Tang, M. Luisa Iruela-Arispe, Tsutomu Kume

**Affiliations:** 1 Feinberg Cardiovascular and Renal Research Institute, Department of Medicine,; 2 Department of Dermatology,; 3 Division of Thoracic Surgery/Canning Thoracic Institute,; 4 Cardiovascular-Thoracic Surgery and The Heart Center, Stanley Manne Children’s Research Institute, Ann & Robert H. Lurie Children’s Hospital of Chicago, Departments of Pediatrics, Surgery, and Pathology, and; 5 Department of Cell and Development Biology, Feinberg School of Medicine, Northwestern University, Chicago, Illinois, USA.

**Keywords:** Cardiology, Development, Vascular biology, Cardiovascular disease, Expression profiling, Genetic diseases

## Abstract

Enhanced TGF-β signaling caused by mutations in Fibrillin-1 (FBN1) in patients with Marfan syndrome (MFS) leads to myxomatous degeneration of the mitral valve (MDMV). MDMV can result in mitral valve prolapse, severe regurgitation, and sudden cardiac death. However, it remains unknown whether lymphatic vessel (LV) dysfunction contributes to MDMV development in MFS. Here, we show that lymphangiogenesis in murine mitral valves (MVs) begins postnatally. However, this process is inhibited in a mouse MFS model, *Fbn1* mutant (*Fbn1^C1039G/+^*) mice, accompanied by disrupted lymphatic cell-cell junctions, impaired lymphatic drainage, and an abnormally widespread distribution of MHCII^+^ infiltrating macrophages. Treatment of *Fbn1* mutant mice with VEGF-C156S, a selective VEGFR3 agonist, stimulates the ERK and Akt pathways, increases LV density in MVs, and ameliorates MDMV. *Fbn1* mutant MVs display disorganized valvular endothelial cells (VECs) and decreased expression of the antiinflammatory modulator *Zfp36* (zinc finger protein 36) in VECs and immune cells. Treatment with FTY720 (fingolimod), a ZFP36 activator and S1P antagonist, rescues MDMV phenotypes in *Fbn1* mutant mice by reducing immune cell infiltration and restoring lymphatic cell junctions and drainage. These findings suggest that the *Fbn1* mutation causes LV hypoplasia and defective lymphatic drainage in MVs, driven in part by proinflammatory VECs, leading to MFS-related MDMV.

## Abstract 2

Lymphatic dysfunction, linked to inflammation, contributes to mitral valve disease in a mouse model of Marfan syndrome. Treatments that restore lymphatic function reverse valve abnormalities.

## Graphical abstract

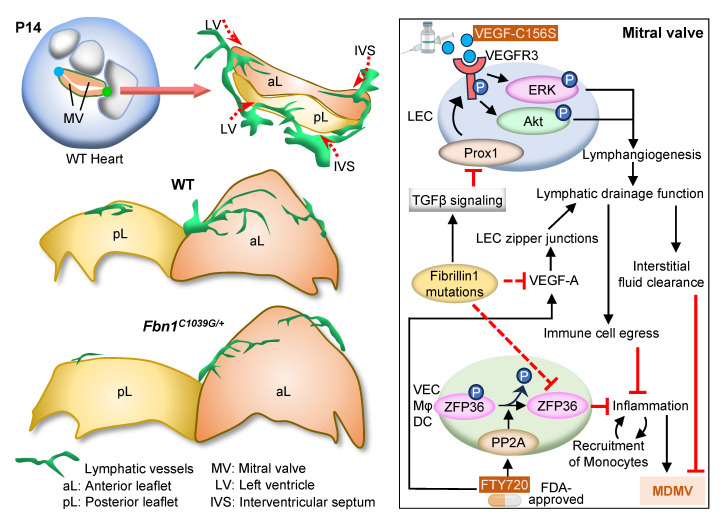

## Introduction

Marfan syndrome (MFS) is an autosome-dominant genetic disorder that chronically affects connective tissue, particularly in the skeletal, cardiovascular, and ocular systems, as well as the skin. More than 45% of MFS patients have mitral valve prolapse (1, 2), a condition caused by myxomatous degeneration of the mitral valve (MDMV) (3), which can lead to severe regurgitation and, ultimately, sudden cardiac arrest or death. However, the pathophysiology of MDMV in MFS remains only partially understood. The mitral valve (MV) consists of 2 leaflets composed of a highly organized, stratified ECM, valve interstitial cells, and an outer layer of valvular endothelial cells (VECs). As MDMV progresses, the MVs become thickened and elongated, with abnormal ECM remodeling, including disruption of elastin and collagen fibers and accumulation of proteoglycans (4). In addition to MFS, MV regurgitation is the most common heart valve disease in older patients (5). Therefore, understanding how MV integrity is maintained is crucial for developing new treatments for MDMV.

MFS is usually linked to a heterozygous mutation in the *FBN1* gene, which encodes Fibrillin-1 (FBN1), a glycoprotein in the ECM and a structural component of connective tissue microfibrils (6). FBN1 is produced by various cell types, including fibroblasts (7), vascular smooth muscle cells (8), vascular endothelial cells (9), and cultured human lymphatic endothelial cells (LECs) (10). FBN1 controls the bioavailability of TGF-β1, a vital cytokine that influences numerous cellular activities (11), including the suppression of lymphangiogenesis (12–14). Mutations in *FBN1* disrupt TGF-β bioavailability in the ECM, leading to excessive TGF-β signaling, a major factor in MFS development (15), including the MDMV. Our recent study uniquely showed the distribution of LVs within adult mouse MVs and confirmed that lymphatic drainage of interstitial fluid is crucial for maintaining MV structure by regulating ECM components (16). However, it remains unclear how LVs develop in MVs, whether *FBN1* mutations in MFS hinder the formation and function of LVs in MVs during MDMV development, and whether lymphatic targeting could be an effective treatment for MDMV.

Recent evidence indicates that various immune cells, including proinflammatory macrophages, accumulate in MDMV across species, including mice (17), sheep (18), and humans (19), as well as in myxomatous valves from genetically engineered pigs and human patients with MFS (20). Infiltrating immune cells are predominantly derived from CCR2^+^ classical monocytes circulating in the blood (20). LVs drain excess interstitial fluid and facilitate the transport of immune cells from the surrounding tissue to draining lymph nodes (21). We recently showed that although no blood vessels are present within murine MVs, small molecules such as Evans blue in the bloodstream can enter the MV interstitium through the permeable VEC layer and be drained into MV lymphatics (16). However, it remains to be determined whether the VEC layer contributes to recruiting inflammatory immune cells and how immune cell trafficking is altered in the MVs of MFS.

In this study, we investigated the development of LVs in mouse MVs. Postnatally, preexisting LVs from the left ventricle and interventricular septum grew into the MVs via lymphangiogenesis and continued to expand in the anterior MV leaflet (aL) until P14 and in the posterior leaflet (pL) until P21. In contrast, *Fbn1* mutant (*Fbn1^C1039G/+^*) mice, a model of MFS that replicates MDMV and aortic aneurysm (22, 23), showed reduced lymphangiogenesis, disrupted LEC junctions, and impaired lymphatic drainage in MVs. More importantly, treatment with VEGF-C156S, a selective agonist of VEGFR3, significantly promoted lymphatic vessel (LV) growth in *Fbn1* mutant MVs, thereby alleviating MV thickening. Additionally, our scRNA-seq analysis revealed a notable decrease in the expression of the antiinflammatory factor Zinc finger protein 36 (*Zfp36*) in VECs, macrophages, and DCs in *Fbn1* mutant MVs. ZFP36 functions as an RNA-binding protein that reduces levels of proinflammatory mRNA transcripts (24). In this study, we demonstrate that treatment of *Fbn1* mutant mice with FTY720, a ZFP36 activator and an FDA-approved immunosuppressant widely used in patients with relapsing multiple sclerosis (25), reduced immune cell infiltration, repaired cell-cell junctions in both VECs and LECs, improved lymphatic drainage, and rescued MDMV phenotypes. Overall, our research highlights the importance of lymphatic dysfunction in the progression of MDMV in MFS and characterizes therapeutic strategies aimed at restoring MV structure by promoting lymphangiogenesis with VEGF-C156S and by immunosuppression with FTY720.

## Results

### Development of LVs in the mouse MVs.

**Figure 1:**
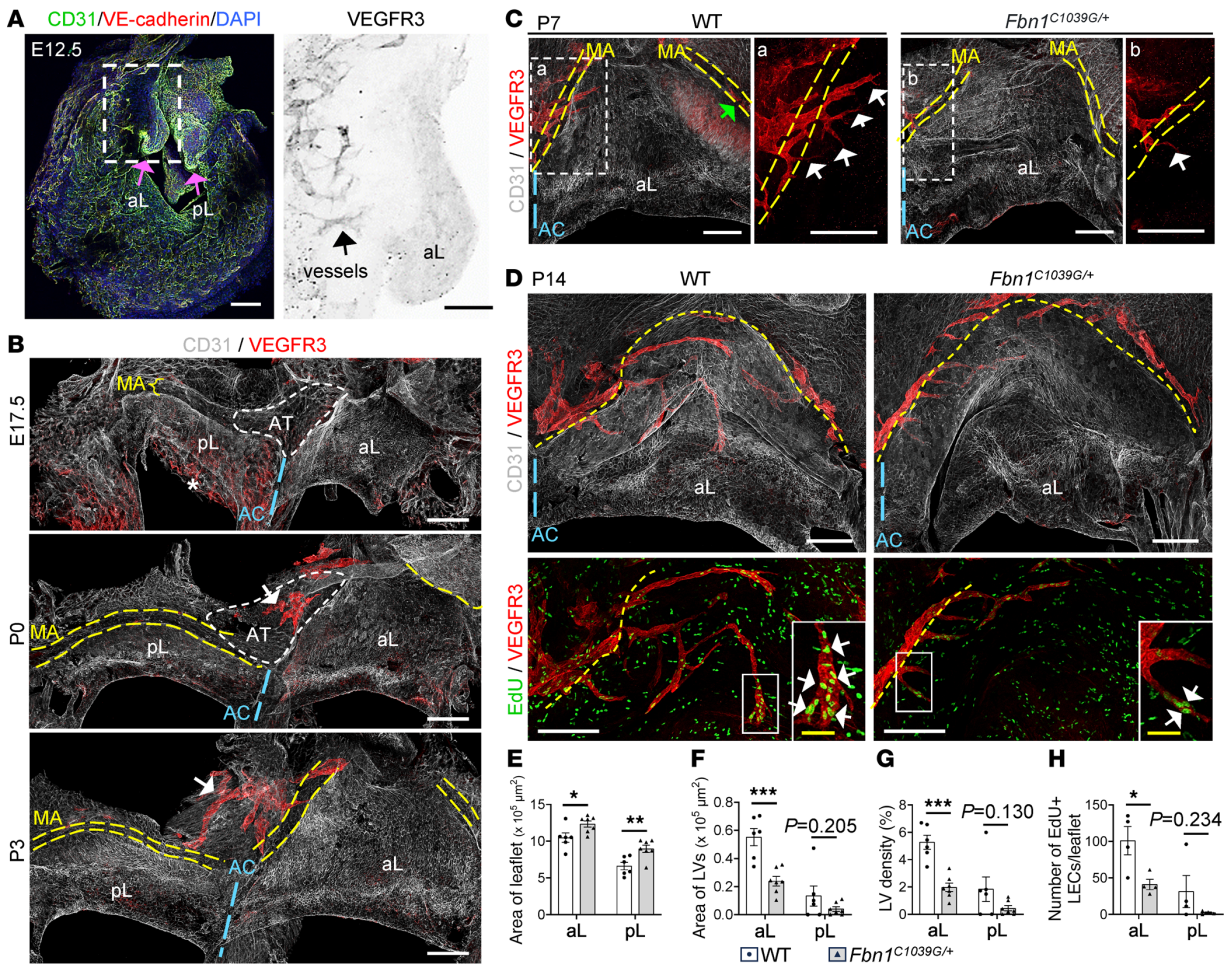
Development of LVs is inhibited in *Fbn1* mutant MVs. Whole-mount immunostaining followed by confocal imaging was performed on hearts and MV samples. (**A**) *Z*-stack (left) and optical section (right) images show vasculatures near MV leaflets in WT mouse at E12.5. White and black scale bars: 100 and 50 μm, respectively. (**B**) Development of LVs in MVs of WT mice at early stages. LVs (VEGFR3^+^, indicated by arrows) appear in the anterior triangle (AT; outlined by a white dashed line) above the anterior commissure (AC) at P0 before sprouting and penetrating the mitral annulus (MA) at P3. The asterisk indicates the left ventricular wall at the fibrosa side of the pL. Scale bars: 200 μm. (**C**) Lymphatic sprouting in the aL at P7. White and green arrows indicate lymphatic sprouts from the sides of the anterior (AC) and posterior commissure, respectively. Scale bars: 200 μm. (**D**) Lymphatic sprouting in aLs at P14. Arrows indicate the EdU^+^ LECs. Yellow lines outline the proximal edge of MV leaflets. White and yellow scale bars: 200 and 50 μm, respectively. (**E**–**H**) Quantification of MV leaflet area (**E**), LV area (**F**), LV density (**G**), and number of EdU^+^ LECs (**H**) based on **D** and Supplemental Figure 2B. Data are mean ± SEM, unpaired 2-tailed Student’s *t* test, each symbol represents 1 mouse. *N* = 6–7 in **E**–**G** (male/female = 3–4:3 per group), *N* = 4 in **H** (male/female = 3:1 and 1:3 in WT and mutant, respectively), *P < 0.05, **P < 0.01, ***P < 0.001.

Using whole-mount immunostaining with the LEC marker VEGFR3, we first examined LV development in the MVs of WT mice at prenatal and postnatal stages. No LVs were observed in the MV leaflets at both E12.5 (Figure 1A) and E17.5 (Figure 1B). At P0, newly formed VEGFR3^+^ lymphatics were first identified in an inverted triangular region above the anterior commissure (Figure 1B). We named this region the anterior triangle, located adjacent to the aLs and pLs and partially containing the mitral annulus that connects both leaflets (Figure 1B). The anterior triangle could be visualized when the heart was opened after being cut through the posterior commissure, interventricular septum, and right ventricular wall (Supplemental Figure 1, A and B; supplemental material available online with this article; https://doi.org/10.1172/JCI195507DS1). VEGFR3^+^ lymphatics in the anterior triangle formed sprouts at P3 (Figure 1B), which elongated and penetrated the aL by P7 (Figure 1C, inset a). The lymphatics in the aL originated not only from the anterior triangle but also from the posterior triangle, an opposite region we named, located at the posterior commissure. The posterior triangle was visible when the heart was opened after being cut through the anterior commissure and left ventricular lateral wall (Supplemental Figure 1, A and C). Lymphatics originating from the posterior triangle were first observed in the mitral annulus of the aL near the posterior commissure at P7 (Figure 1C, green arrow) and then formed sprouts penetrating the aL at P10 (Supplemental Figure 2A, inset a). By P14, lymphatics were well developed in the aL of WT mice (Figure 1D). In contrast, LV formation in the pL occurred later than in the aL. VEGFR3^+^ LVs were first detected in the mitral annulus of the pL at P10 and formed branches in the pL by P14 (Supplemental Figure 2B).

To further explore how the LVs infiltrated the MV leaflets from other parts of the heart, we conducted whole-mount immunostaining for VEGFR3 on P14 hearts from the *Prox1-eGFP* reporter mouse line (26) (Supplemental Figure 1D). 3D reconstruction was then performed to localize cardiac lymphatics. For the anterior MV leaflet, LV extension occurred from the LVs at both the anterior and posterior triangles at P14 (Supplemental Videos 1 and 2), as described above. In the anterior triangle, lymphatics sprouted from the LVs in the subepicardium of the left ventricular lateral wall (Supplemental Figure 1D, inset a, and Supplemental Video 1), while those in the posterior triangle originated from the LVs on the right side of the interventricular septum (Supplemental Figure 1D, inset b, and Supplemental Video 2). The posterior MV leaflet contained LVs sprouting from both the anterior and posterior triangles, as well as from the dorsal wall of the left ventricle and the right posterior side of the interventricular septum (Supplemental Figure 1D, VEGFR3 channel, white arrows, and Supplemental Videos 1 and 2). Although lymphatics were observed in the aortic annulus at P14 (Supplemental Figure 1D, inset a), no LVs were detected within the aortic valve leaflets even at P21 (Supplemental Figure 1E).

Together, these results suggest that the lymphatics in the MV leaflets develop after birth, sprouting from the preexisting LVs in the left ventricle and interventricular septum.

### Hypoplastic LV formation in the MVs of Fbn1 mutant mice.

Next, we examined LV development in the MVs of the MFS mouse model, *Fbn1* heterozygous mutant (*Fbn1^C1039G/+^*) mice, starting at P7, using whole-mount immunostaining for VEGFR3. Compared with WT MVs, lymphatic growth was reduced in *Fbn1* mutant MVs (Figure 1, C–H, and Supplemental Figure 2, A and B). In aLs of *Fbn1* mutant mice, the number of lymphatic sprouts from the anterior triangle was decreased at P7 (Figure 1C, inset b) and P10 (Supplemental Figure 2A), and no lymphatics had formed yet in the posterior triangle at P10 (Supplemental Figure 2A, inset b). LVs in *Fbn1* mutant pLs were first observed in the mitral annulus at P14, much later than in WT mice (Supplemental Figure 2B). At P21, lymphatics in the mitral annulus formed sprouts that penetrated the *Fbn1* mutant pLs, whereas in WT pLs, the lymphatics were well developed (Supplemental Figure 2C). Notably, although no significant difference in heart size was detected between *Fbn1* mutant and WT mice before P14 (Supplemental Figure 2D), the MV leaflets of *Fbn1* mutants were already larger than those of WT mice at P14 (Figure 1, D and E, and Supplemental Figure 2B), indicating early-stage MDMV. Most importantly, at P14, *Fbn1* mutant MVs showed a significant reduction in lymphatic area and density in aLs and a decreasing trend of these parameters in pLs (Figure 1, D, F, and G, and Supplemental Figure 2B). EdU (5-ethynyl-2′-deoxyuridine) staining further confirmed the lower number of proliferating LECs in *Fbn1* mutant MVs compared with WT MVs at this time point (Figure 1, D and H). Further characterization using the marker LYVE1 confirmed a consistent reduction in LV density within *Fbn1* mutant MVs (Supplemental Figure 3). Together, these data indicate that the *Fbn1* heterozygous mutation results in hypoplastic LV development in MVs.

### Disrupted lymphatic cell junctions and impaired lymphatic drainage in the MVs of Fbn1 mutant mice.

**Figure 2:**
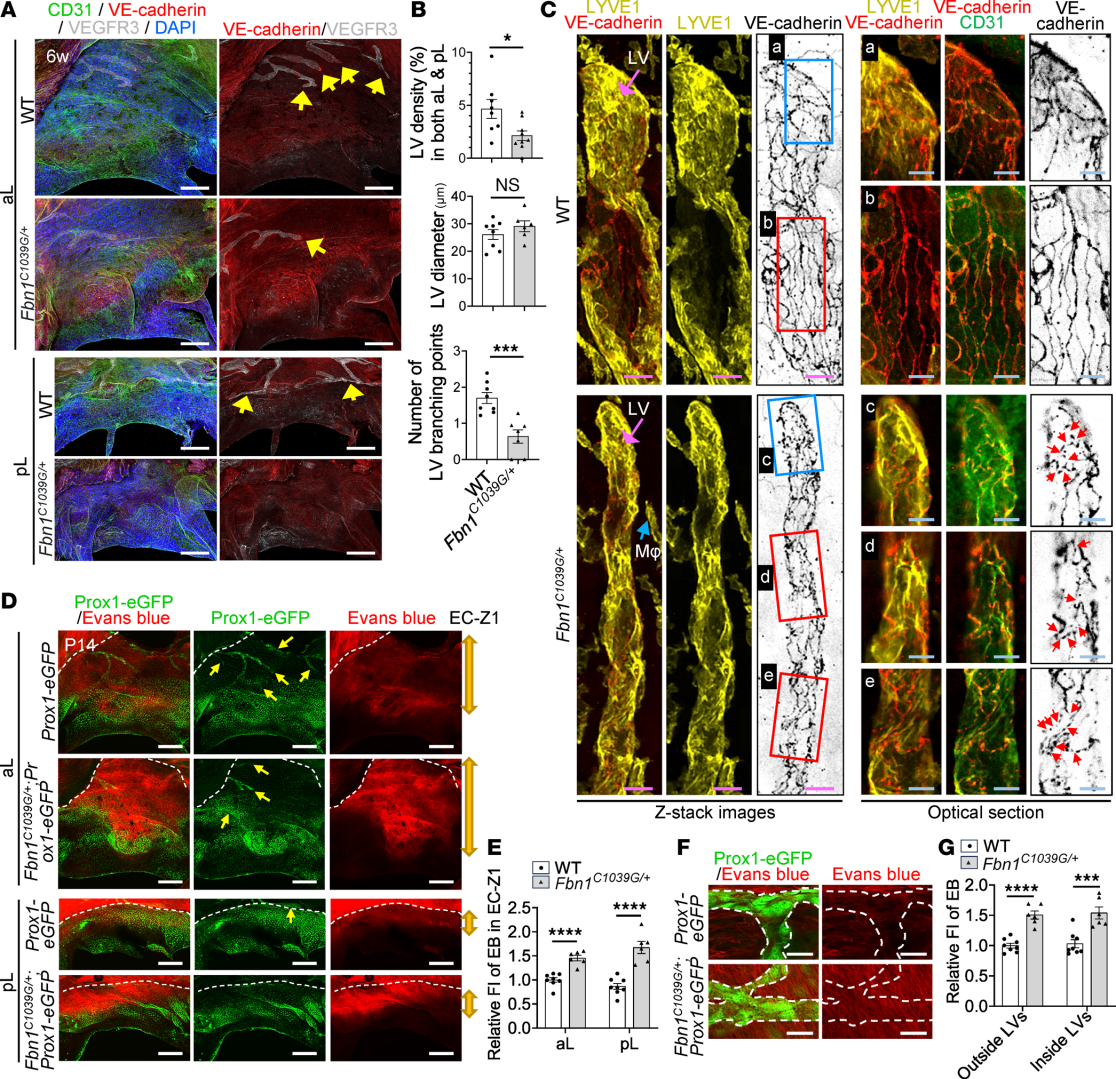
Lymphatic defects in *Fbn1* mutant MVs. (**A**) Representative confocal images of whole-mount leaflets at 6 weeks. Arrows indicate LVs. Scale bars: 200 μm. (**B**) Quantification of lymphatic density in the total leaflet area (both aL and pL) (*N* = 8-9 per group; 5 males, 3-4 females), LV diameter (*N* = 6-8; 3-5 males, 3 females), and number of lymphatic branching points/0.01 mm^2^ of LVs (*N* = 7-8; 3-5 males, 3–4 females). Data are mean ± SEM, unpaired 2-tailed Student’s *t* test, *P < 0.05, ***P < 0.001. (**C**) Representative confocal images of lymphatic capillaries in MVs showing impaired lymphatic cell-cell junctions in *Fbn1* mutant MVs at 6 weeks. Boxed regions are shown at higher magnification in insets a–e. Blue and red boxes represent the tip and proximal segment, respectively, of the LV. Arrows indicate disrupted cell-cell junctions in the mutant compared with the continuous junctions in WT. Pink and blue scale bars: 20 and 10 μm, respectively. (**D**–**G**) Evans blue permeability assay at P14 using mice with *Prox1-eGFP* reporter. *N* = 6–8; 4–5 males, 2–3 females. (**D**) Representative *Z*-stack images of MV leaflets. Prox1-eGFP is expressed not only in LECs but also in VECs in the MV leaflet. Arrows indicate LVs. White dashed lines show the proximal edge of the MV leaflets. EC-Z1, EC zone 1. Scale bars: 200 μm. (**F**) Representative optical section images showing Evans blue outside and inside LVs (outlined by dashed lines). Scale bars: 20 μm. Quantification was performed for the fluorescence intensity (FI) of Evans blue (EB) in the interstitium beneath EC zone 1 for both aLs and pLs (**E**), and outside/inside LVs (**G**). Data are mean ± SEM, unpaired 2-tailed Student’s *t* test, each symbol represents 1 mouse, ***P < 0.001, ****P < 0.0001.

As described at the postnatal time points in Figure 1, we further identified mature LVs in MVs of 6-week-old WT mice (Figure 2A). In contrast, *Fbn1* mutant MVs showed decreased LV density and fewer LV branching points, although LV diameter was comparable with that of WT mice (Figure 2B). Close examination of whole-mount staining with the cell-cell junction markers VE-cadherin and CD31 revealed that LEC junctions in lymphatic capillaries of WT MVs appeared continuous, and the cells were elongated, similar to LECs with zipper-like junctions in collecting lymphatics (Figure 2C, insets a and b; Supplemental Figure 4A). However, a small number of LECs with an oak leaf–like shape and discontinuous button-like junctions were found only at the tips of WT lymphatic capillaries (Supplemental Figure 4, B and C). This pattern of LEC junctions differed from the overall button-like junctions present in lymphatic capillaries of other tissues such as the trachea, diaphragm, dermis, and small intestine (27–30), but was similar to the pattern reported in initial lymphatic capillaries in the lung (31). In contrast, lymphatic capillaries in *Fbn1* mutant MVs exhibited disrupted LEC junctions along their entire length (Figure 2C, insets c–e; Supplemental Figure 4, A and C), suggesting that the *Fbn1* mutation disrupts LEC junctions in MVs. Interestingly, the junctional defects observed in the *Fbn1* mutant mice were MV specific, as no discernible differences in junctional morphology were found in the lymphatic capillaries of the small intestine, diaphragm, or skin compared with WT mice (Supplemental Figure 5, A–D).

To examine changes in lymphatic drainage function in *Fbn1* mutant MVs, we treated *Fbn1* mutant mice crossed with the *Prox1-eGFP* reporter line with Evans blue through retro-orbital injection at P14. As we recently reported, there are no blood vessels in the MVs, but Evans blue can penetrate the MVs via the permeable VEC layer and be drained as part of interstitial fluid through the LVs within the MVs (16). Sixty minutes after injection, Evans blue accumulated significantly more in both MV leaflets of *Fbn1* mutant mice than in WT MVs (Figure 2, D and E). It should be noted that *Prox1-eGFP*^+^ cells include MV LECs (Figure 2D, arrows) and VECs. Evans blue was mainly retained in the MV interstitium near EC zone 1, a VEC zone at the proximal atrial side of the MV leaflets (aL and pL), where the LVs are located (16) (Figure 2D and Supplemental Figure 6). In WT aLs, Evans blue was less abundant in the interstitium near the LVs in EC zone 1, whereas in *Fbn1* mutant aLs, high Evans blue fluorescence was observed in the interstitium near the LVs (Figure 2D and Supplemental Figure 6). Further examination revealed significantly greater accumulation of Evans blue both outside and inside LVs in *Fbn1* mutant MVs compared with WT MVs (Figure 2, F and G). These data suggest that lymphatic drainage function in *Fbn1* mutant MVs is impaired, leading to excess interstitial tissue fluid.

To determine whether the *Fbn1* mutation also affects the formation and function of cardiac LVs in the heart, beyond MVs, we next performed whole-mount immunostaining with the LEC markers VEGFR3 and LYVE1 on P14 hearts. *Fbn1* mutant hearts had fewer branched LVs in the subepicardium of the ventricular walls than WT hearts (Supplemental Figure 7, A–D). Consistent with this finding, we observed a significant increase in the wet/dry weight ratio of 3- to approximately 6-month-old *Fbn1* mutant hearts, indicating cardiac edema in adult *Fbn1* mutant mice (Supplemental Figure 7E).

### VEGF-C156S rescues lymphatic defects in MVs and MDMV phenotypes in Fbn1 mutant mice.

**Figure 3:**
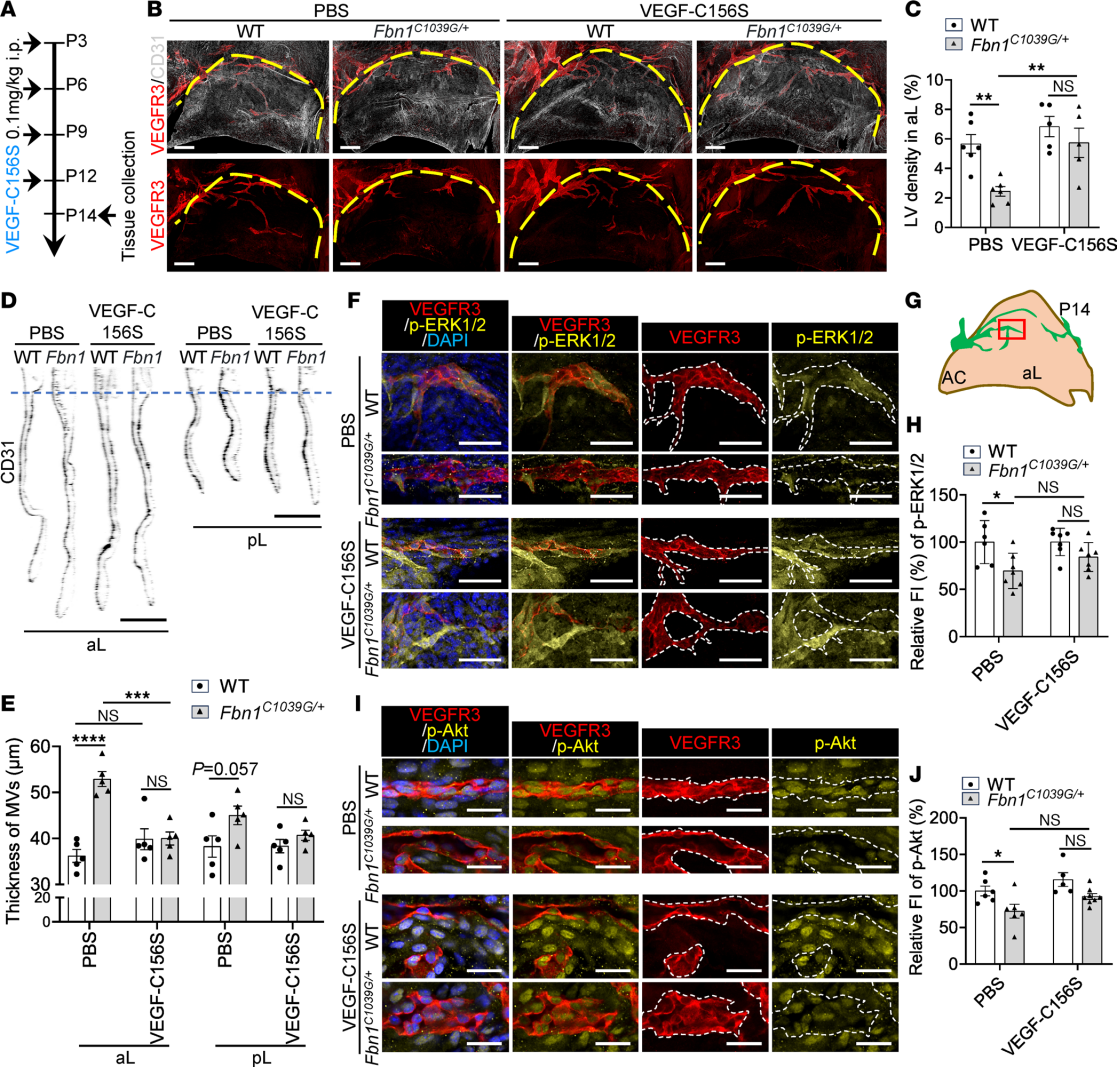
VEGF-C156S promotes lymphatic growth in the MVs and ameliorates MV thickening in *Fbn1* mutant mice by activating the ERK and Akt signaling pathways. (**A**) Timeline for the VEGF-C156S treatment in **B**–**J**. (**B**) Representative confocal images of whole-mount aLs at P14 under PBS (vehicle control) and VEGF-C156S treatment. Yellow dashed lines indicate the proximal edge of leaflets. Scale bars: 200 μm. (**C**) Quantification of LV density in aLs based on the data shown in **B**. Data are mean ± SEM, ordinary 1-way ANOVA, each symbol represents 1 mouse. *N* = 5-6, male/female = 2:4, 3:3, 3:2, and 3:2 in the WT-PBS, *Fbn1*-PBS, WT-VEGF-C156S, and *Fbn1*-VEGF-C156S groups, respectively. **P < 0.01. (**D**) Resliced 2D images from the 3D image stacks of whole-mount MV leaflets along the midline of each leaflet. Blue dashed line indicates the root of leaflets. Scale bars: 200 μm. (**E**) Quantification of MV leaflet thickness based on the data shown in **D**. Data are mean ± SEM, ordinary 1-way ANOVA test for aLs, unpaired 2-tailed Student’s *t* test for pLs, each symbol represents 1 mouse. *N* = 5, male/female = 2:3. ***P < 0.001, ****P < 0.0001. (**F**–**J**) Representative confocal images of LVs labeled with p-ERK1/2 (**F**) or p-Akt (**I**) in the boxed region of aLs at P14 (**G**) and quantification of fluorescence intensity (FI) of these markers in LECs (**H** and **J**). Scale bars: 50 μm (**F**), 20 μm (**I**). Data are mean ± SEM in **H** and **J**, ordinary 1-way ANOVA, each symbol represents 1 mouse. *N* = 6-7 in **H**, male/female = 2:4 in PBS-treated WT, 4:3 in other groups; *N* = 5-8 in **J**, 2-5 males and 3 females. *P < 0.05.

VEGF-C promotes angiogenesis and lymphangiogenesis by binding to its receptors, VEGFR2 (primarily expressed on blood endothelial cells) and VEGFR3 (expressed on LECs) (32). VEGF-C156S, an engineered variant of VEGF-C that specifically activates the VEGFR3 receptor, promotes lymphangiogenesis without significant angiogenesis (33). VEGF-C156S treatment enhances lymphangiogenesis and improves cardiac injury in sepsis-induced cardiomyopathy (34). To investigate whether VEGF-C156S treatment can restore lymphatic defects in *Fbn1* mutant MVs and thereby rescue the MDMV phenotypes, we intraperitoneally injected WT and *Fbn1* mutant mice with VEGF-C156S or PBS (vehicle control) at P3, P6, P9, and P12, then collected their MVs at P14 for whole-mount immunostaining (Figure 3A). The LV density in aLs was assessed because lymphatic development in the pL was incomplete at P14 (Figure 1). In PBS-treated groups, *Fbn1* mutant MVs exhibited lower LV density in aLs compared with WT mice, while LV density in aLs of VEGF-C156S–treated *Fbn1* mutant MVs was significantly higher than in PBS-treated *Fbn1* mutant MVs and was similar to that of aLs in WT mice treated with VEGF-C156S (Figure 3, B and C). Since lymphatic function is crucial for maintaining MV structure (16), we further analyzed MV leaflet thickness. In PBS-treated groups, *Fbn1* mutant mice showed significantly thicker aLs and a trend toward thicker pLs than WT mice at P14 (Figure 3, D and E), which is earlier than previously reported (P30) (20). Remarkably, no significant difference in MV leaflet thickness was observed between WT and *Fbn1* mutant mice treated with VEGF-C156S (Figure 3, D and E). These findings suggest that VEGF-C156S treatment not only stimulates MV lymphatic growth but also reduces abnormal MV thickening in *Fbn1* mutant mice.

Next, we examined ECM organization and inflammatory cell infiltration in *Fbn1* mutant MVs following VEGF-C156S treatment. We observed that VEGF-C156S significantly decreased hyaluronic acid–binding protein (HABP), a marker of proteoglycan deposition, and significantly diminished cell number without affecting cell density, while showing a trend toward increased collagen type I (Supplemental Figure 8, A–C). Additionally, VEGF-C156S treatment reduced CD45^+^ immune cell infiltration in *Fbn1* mutant MVs (Supplemental Figure 8, D and E). These findings indicate that VEGF-C156S ameliorates pathological matrix deposition and reduces inflammation in *Fbn1* mutant MVs.

To further evaluate the long-term efficacy of VEGF-C156S treatment, we examined MVs of *Fbn1* mutant mice at 6 weeks of age, following postnatal VEGF-C156S (or PBS, vehicle control) administration at P3, P6, P9, and P12 (Supplemental Figure 9A). VEGF-C156S–treated *Fbn1* mutant mice showed significantly higher LV density in both aLs and pLs and thinner MV leaflets than the PBS-treated group (Supplemental Figure 9, B–F), suggesting that the therapeutic benefits of early-life VEGF-C156S intervention persist throughout development into adulthood.

### VEGF-C156S restores impaired VEGF-C/VEGFR3 signaling in lymphatics of Fbn1 mutant MVs.

Although TGF-β signaling is enhanced by FBN1 mutations (11) and TGF-β1 overexpression suppresses lymphangiogenesis (12–14), the mechanisms that regulate lymphangiogenesis in *Fbn1* mutant MVs remain to be elucidated. We therefore confirmed hyperactivation of the TGF-β signaling pathway, as evidenced by increased phospho-SMAD2 (p-SMAD2) levels in lymphatics of *Fbn1* mutant MVs (Supplemental Figure 10, A and B). PROX1, which is suppressed by TGF-β signaling (13) and directly regulates *Vegfr3* expression (35), was decreased in the lymphatics of *Fbn1* mutant MVs compared with WT MVs at P14 (Supplemental Figure 10, C and D). VEGF-C binds to VEGFR3, triggering autophosphorylation of the intracellular VEGFR3 tyrosine kinase domains (36), which activates the MAPK/ERK and PI3K/Akt signaling cascades, thereby promoting LEC proliferation, survival, and migration (37). To determine whether VEGF-C/VEGFR3 signaling is disrupted in *Fbn1* mutant MVs and whether VEGF-C156S treatment can effectively stimulate this pathway, we evaluated *Fbn1* mutant MVs at P3 and P14, before and after VEGF-C156S administration, respectively. At P3, in lymphatics restricted to the anterior triangle (Figure 1B and Supplemental Figure 10E), we observed a trend toward reduced VEGFR3 phosphorylation (p-VEGFR3) in LECs of *Fbn1* mutant mice, whereas activation of the downstream effectors ERK1/2 (p-ERK1/2) and Akt (p-Akt) was significantly decreased compared with WT mice (Supplemental Figure 10, F–K). By P14, all 3 signaling indicators were significantly diminished in the lymphatics of MV leaflets in PBS-treated (vehicle control) *Fbn1* mutant mice. Notably, VEGF-C156S administration effectively upregulated these markers, restoring them to levels comparable with those of the WT (Figure 3, F–J, and Supplemental Figure 10, L and M). These data suggest that VEGF-C156S restores impaired VEGF-C/VEGFR3 signaling in the lymphatics of *Fbn1* mutant MVs.

### Proinflammatory VECs and increased immune cell accumulation in Fbn1 mutant MVs at an early age.

**Figure 4:**
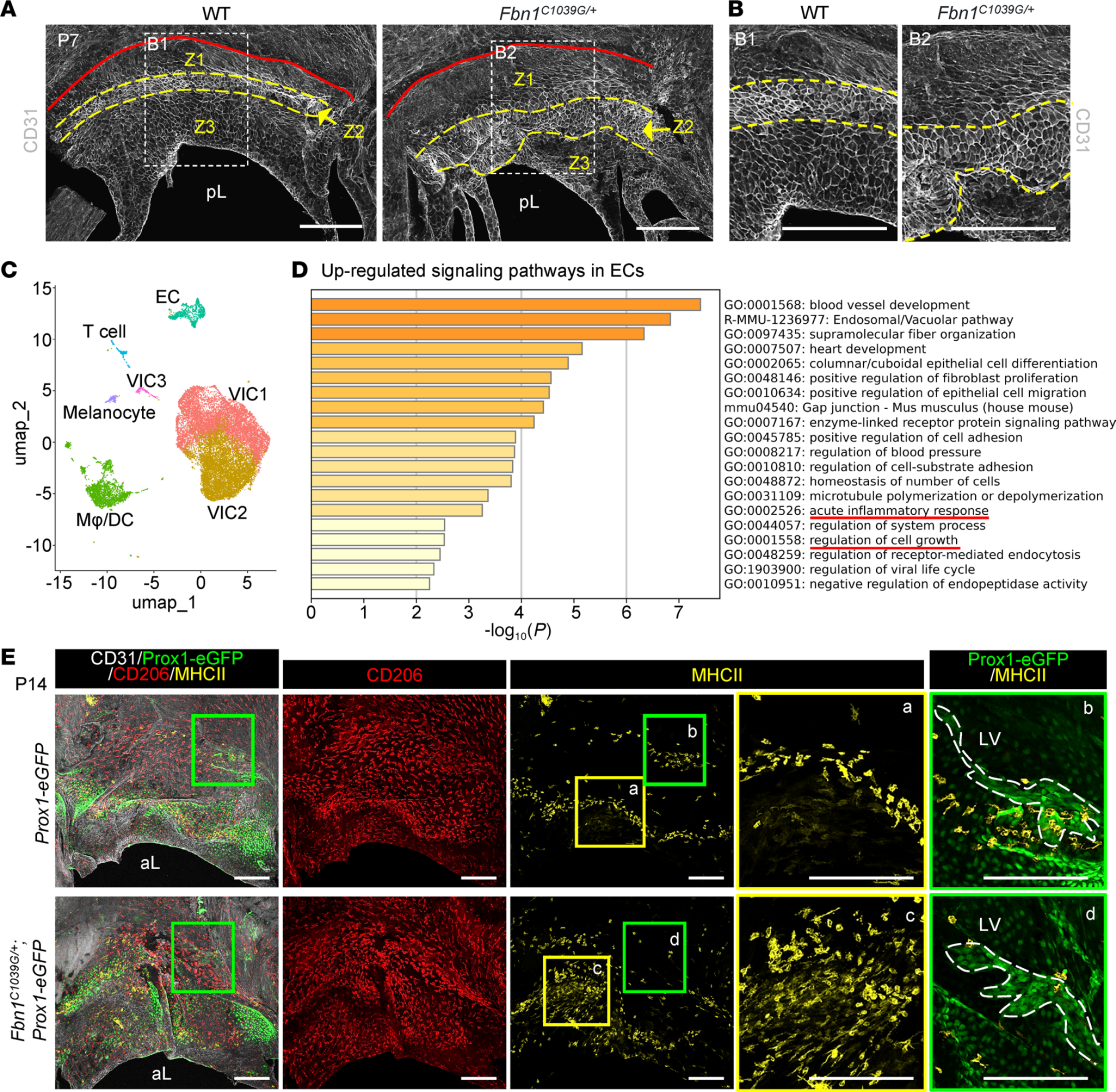
Inflammation is increased in MVs of *Fbn1* mutant mice. (**A** and **B**) Representative confocal images of whole-mount pLs showing larger leaflet and disorganized VECs (CD31^+^) in *Fbn1* mutant mouse at P7. The boxed regions in **A** are shown at higher magnification in **B**. Scale bars: 200 μm. (**C**) New analysis of published scRNA-seq data (GSE261874) shows a UMAP of cell clusters in MVs of both WT and *Fbn1* mutant mice at P30. (**D**) Gene Ontology analysis shows upregulated signaling pathways in the EC cluster of *Fbn1* mutant MVs. (**E**) Representative confocal images show the distribution of CD206^+^ tissue-resident macrophages and MHCII^+^ infiltrating immune cells in whole-mount aLs. Scale bars: 200 μm.

The lymphatic system is not only vital for maintaining fluid balance but also plays important roles in immune responses and in facilitating immune cell trafficking in various tissues (21). CCR2^+^, monocyte-derived MHCII^+^ infiltrating macrophages accumulate in MVs of MFS mice (*Fbn1* mutants) at 2 months of age (20). However, it remains unclear how monocytes are recruited to the MVs of *Fbn1* mutant mice and whether the accumulation of infiltrating macrophages is linked to lymphatic dysfunction in these mutant MVs. Therefore, we examined whether the *Fbn1* mutation causes abnormalities in VECs, which act as permeable EC barriers and are crucial for regulating ECM within MVs (16). VEC zones (16) were first analyzed by whole-mount immunostaining with CD31 in MVs. VEC zones were identified based on cell size, shape, and distribution on both sides of the MV leaflet, as recently reported (16). VEC zones 1–3 are located on the atrial side, while zones 4–6 are on the fibrosa side of the MV leaflet. VECs in zone 2 are notably smaller and exhibit stronger CD31 expression than those in zones 1 and 3. Results showed that by P7, the organization of VEC zones was significantly altered in *Fbn1* mutant MVs compared with WT mice, with enlargement of zones 1 and 2 on the atrial side (Figure 4, A and B). To analyze the molecular and cellular changes in *Fbn1* mutant MVs, including VECs, we performed a secondary analysis of published scRNA-seq data (38). The original datasets were derived from P30 WT and *Fbn1* mutant MVs. We identified cell clusters consistent with those previously reported, including endothelial cells (ECs), valve interstitial cells, macrophages/DCs, T cells, and melanocytes (Figure 4C). Within the EC cluster, we identified a small, uncharacterized population of LECs using markers such as *Reln*, *Lyve1*, *Flt4*, and *Pdpn* (Supplemental Figure 11, A–C). Most cells in the EC cluster were VECs. Gene Ontology analysis of the EC cluster revealed enrichment of pathways involved in cell growth and inflammatory responses, consistent with the focus of our study and paralleling the phenotypic defects observed in *Fbn1* mutant VECs (Figure 4D). Inflamed ECs attract proinflammatory classical monocytes from the blood, promoting their extravasation through the EC layer and subsequent differentiation into macrophages and DCs (39). Although immune cell accumulation has been observed in 2-month-old *Fbn1* mutant MVs (20), flow cytometry showed a significant increase in CD45^+^ immune cells in *Fbn1* mutant MVs at P14 (Supplemental Figure 12, A and B). At P14, immune cells were primarily T cells and CD11b^+^ myeloid cells, predominantly macrophages, monocytes, and DCs (Supplemental Figure 12, C and D). There was a trend toward more tissue-resident macrophages (CD206^+^ macrophages) (Supplemental Figure 12D), along with a significant increase in infiltrating CCR2^+^ cells (CCR2^+^ monocytes and macrophages) and MHCII^+^ cells (MHCII^+^ monocytes, macrophages, and DCs) (Supplemental Figure 12E) in *Fbn1* mutant MVs compared with WT MVs, indicating heightened inflammation in the MVs of *Fbn1* mutant mice even at an early age.

Whole-mount immunostaining of MVs isolated from P14 *Fbn1* mutant and WT mice carrying the *Prox1-eGFP* reporter also confirmed that MHCII^+^ infiltrating immune cells accumulated much more in *Fbn1* mutant MVs than in WT MVs (Figure 4E). Notably, MHCII^+^ immune cells in WT MVs were distributed as a narrow stripe beneath EC zone 2 (Figure 4E, inset a) and were more scattered near LVs in EC zone 1 (Figure 4E, inset b), indicating immune cell migration toward functional lymphatics. In contrast, *Fbn1* mutant MVs showed a disrupted distribution of MHCII^+^ immune cells, with increased numbers in the interstitium beneath EC zones 1–4 (Figure 4E, inset c) but very few surrounding the LVs (Figure 4E, inset d). This aligns with the impaired lymphatic drainage observed in *Fbn1* mutant MVs (Figure 2). CCR7, the receptor for the LEC-derived CCL21 and a mediator of immune cell guidance to the lymphatic system (40), was significantly downregulated in CD45^+^ immune cells within *Fbn1* mutant MVs compared with WT (Supplemental Figure 13, A and B). This decrease correlates with the observed accumulation of immune cells, further suggesting impaired lymphatic egress in *Fbn1* mutant MVs.

These data suggest that increased recruitment of monocytes through proinflammatory *Fbn*1 mutant VECs leads to abnormal accumulation of infiltrating immune cells in MVs, which is further exacerbated by LV dysfunction in immune cell trafficking.

### Reduced expression of Zfp36 across different cell types in Fbn1 mutant MVs.

**Figure 5:**
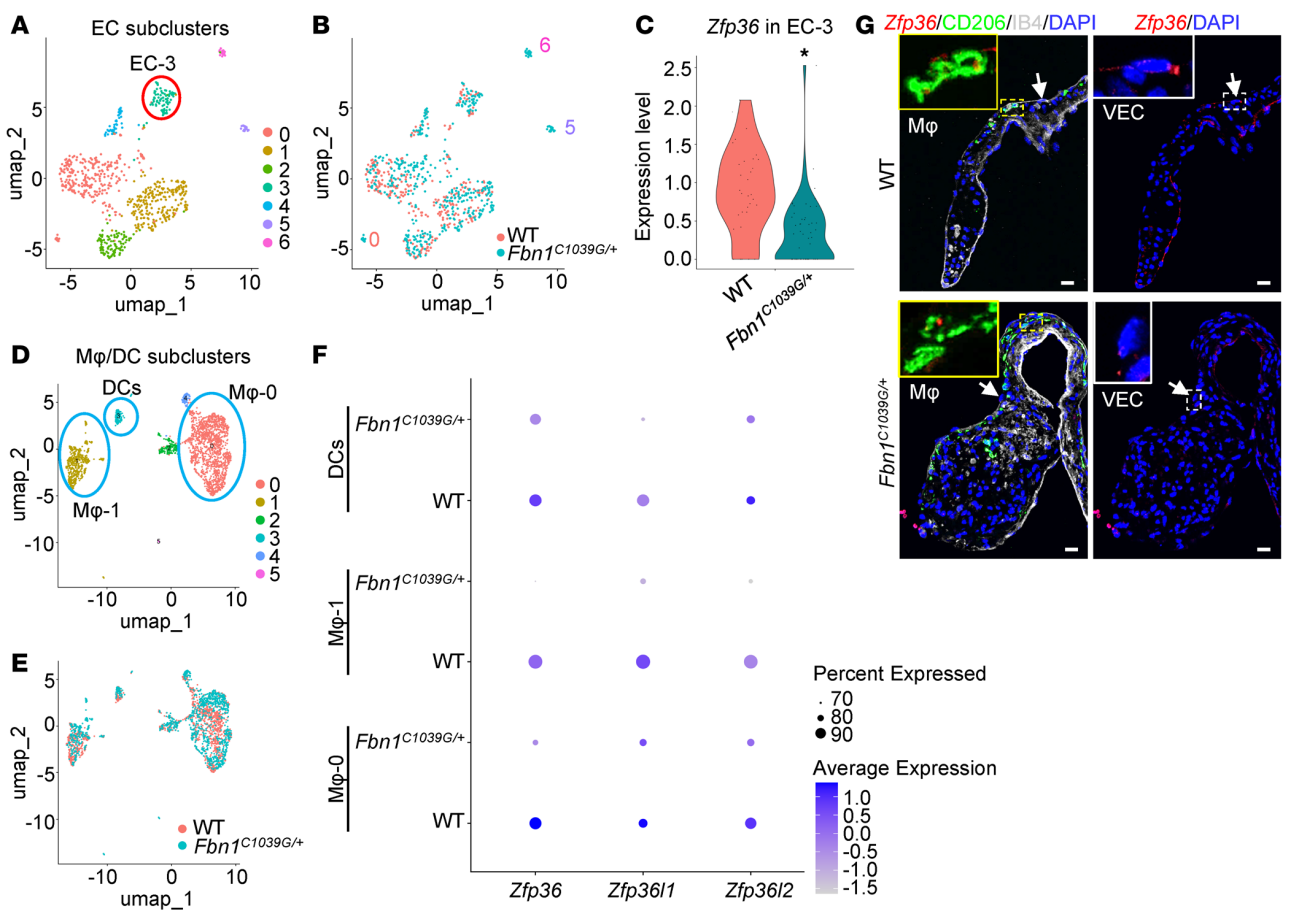
*Zfp36* levels are decreased in VECs, macrophages, and DCs in MVs of *Fbn1* mutant mice. (**A**) UMAP visualization of subclusters (0-6) of total ECs in MVs. (**B**) UMAP of EC subclusters from WT and *Fbn1* mutant MVs. Three new EC subclusters were identified (EC-5, EC-6, and a subcluster of EC-0) in *Fbn1* mutant MVs. (**C**) Violin plots of *Zfp36* expression in EC subcluster 3 (EC-3). *Adjusted *P* < 0.05. (**D**) UMAP visualization of subclusters within the macrophage/DC cluster in Figure 4C. Macrophage subclusters 0, 1, 2, 4, and 5 as well as DCs (subcluster 3) were identified based on their top-expressed markers shown in Supplemental Figure 14, B and C. (**E**) UMAP of macrophage/DC subclusters from WT and *Fbn1* mutant mice. (**F**) Dot plot showing gene expression of *Zfp36*, *Zfp36l1*, and *Zfp36l2* in 3 cell clusters in WT and *Fbn1* mutant MVs at P30. Fill colors represent normalized mean expression levels, and circle sizes represent the within-cluster frequency of positive gene detection. The adjusted *P* values for *Zfp36*, *Zfp36l1*, and *Zfp36l2*: in Mφ-0, adj. *P* = 3.72 × 10^–93^, 1, and 0.000231, respectively; in Mφ-1, adj. *P* = 2.12 × 10^–41^, 8.44 × 10^–9^, and 1.52 × 10^–7^, respectively; in DCs, adj. *P* = 0.0402, 1, and 1, respectively. (**G**) RNAscope combined with IHC staining shows the decreased expression of *Zfp36* in CD206^+^ macrophages and VECs (IB4^+^) in *Fbn1* mutant MVs at P30. Scale bars: 10 μm.

To investigate how VECs in *Fbn1* mutant MVs transition into a proinflammatory state, we performed subclustering of the EC cluster (mostly VECs) in the scRNA-seq datasets described above (Figure 4C). Combining WT and *Fbn1* mutant ECs at P30, we identified 7 EC subclusters (EC-0 to EC-6) (Figure 5A and Supplemental Figure 14A). Among these, EC-5, EC-6, and a subcluster of EC-0 were found only in *Fbn1* mutant MVs (Figure 5B), indicating the emergence of new VEC subtypes in the mutant mice. By analyzing differentially expressed genes (DEGs) in EC-0 to EC-4, we found a significant reduction in expression of the antiinflammatory modulator *Zfp36*, the only DEG identified in EC-3 (Figure 5C and Supplemental Table 1). ZFP36, also known as tristetraprolin, is an RNA-binding protein that promotes the degradation of proinflammatory cytokine mRNA transcripts, such as TNF-α and IL-6 (41). *Zfp36* global knockout mice develop early-onset, severe inflammation, including valvulitis (42, 43). Patients with MV prolapse have lower ZFP36 expression in peripheral blood mononuclear cells (44). Notably, ZFP36-deficient ECs increase angiogenic sprouting in vitro (45). Therefore, decreased *Zfp36* expression in VECs of *Fbn1* mutant MVs is likely to drive inflammation in VECs.

Next, we performed subclustering of the combined macrophage/DC cluster from WT and *Fbn1* mutant MVs. Six subclusters were identified (Figure 5, D and E, and Supplemental Figure 14B). Subclusters 0, 1, 2, 4, and 5 were macrophages, while subcluster 3 was identified as DCs based on cell type–specific markers (Figure 5D and Supplemental Figure 14, B and C). Among the macrophages, Mφ-0 and Mφ-1 were the major subclusters. Importantly, additional DEG analysis of all macrophage/DC subclusters showed that *Zfp36* was among the top 10 most significant DEGs in the Mφ-0, Mφ-1, and DC clusters (ranked 6th, 2nd, and 10th, respectively; Supplemental Tables 2–4). While *Zfp36* was significantly downregulated in Mφ-0, Mφ-1, and DCs, the other 2 ZFP36 family genes, *Zfp36l1* and *Zfp36l2*, were also significantly reduced in Mφ-1 (both genes) and in Mφ-0 (*Zfp36l2*) in *Fbn1* mutant MVs (Figure 5F). Using RNAscope (RNA in situ hybridization analysis) combined with IHC, we confirmed reduced expression of *Zfp36* in both CD206^+^ macrophages (in most macrophage subclusters; Supplemental Figure 14D) and VECs (Figure 5G). Similar to ZFP36, ZFP36L1 and ZFP36L2 promote the degradation of specific mRNA transcripts containing adenylate/uridylate-rich elements, despite their distinct functions in different immune cell types (41). Myeloid cell–specific deletion of all 3 ZFP36 family members in mice causes a synergistic development of an early-lethal inflammatory syndrome due to excess levels of proinflammatory cytokines and chemokines (46). Therefore, decreased levels of the 3 *Zfp36* family transcripts in macrophages and DCs may contribute to inflammation in *Fbn1* mutant MVs.

These data indicate that reduced expression of the antiinflammatory modulator *Zfp36* in VECs, together with reduced levels of ZFP36 family members (*Zfp36*, *Zfp36l1*, and *Zfp36l2*) in immune cells (macrophages and DCs), contributes to the inflammatory response in *Fbn1* mutant MVs.

### EC-specific deletion of Zfp36 causes inflammation of the MVs.

To investigate whether deleting the antiinflammatory modulator *Zfp36* in VECs can trigger inflammation in MVs, we examined MVs from tamoxifen-inducible, EC-specific *Zfp36* mutant (*Cdh5-Cre^ERT2^ Zfp36^fl/fl^*) mice, referred to as EC-*Zfp36*-KO mice, at P60 after inducing the mutation between P1 and P5 (Supplemental Figure 15A). More melanocytes were observed in the MVs of EC-*Zfp36*-KO mice (Supplemental Figure 15B), and their MVs were significantly thicker than those of control littermates (Supplemental Figure 15, C and D), resembling the MDMV phenotype (16). Whole-mount immunostaining revealed a trend toward increased local accumulation of HABP in the MV leaflets of EC-*Zfp36*-KO mice (Supplemental Figure 15, E–G; water-treated group). Moreover, the number of MHCII^+^ infiltrating immune cells significantly increased in the interstitium beneath EC zone 1 in the MVs of EC-*Zfp36*-KO mice compared with controls (Supplemental Figure 15, F and H; water-treated group). These data suggest that EC-specific deletion of *Zfp36* promotes inflammation in MVs, potentially leading to MDMV.

### FTY720 alleviates MDMV phenotypes in Fbn1 mutant mice.

CCR2^+^ immune cell infiltration promotes MV inflammation in *Fbn1* mutant mice (20). CCR2 genetic knockout (20) or pharmacological inhibition of CCR2^+^ cell infiltration (47) can rescue MDMV phenotypes in *Fbn1* mutant mice, suggesting that inflammation contributes to the progression of MDMV in individuals with MFS. Therefore, we next sought to rescue the MDMV phenotypes in *Fbn1* mutant mice by reducing MV inflammation through upregulating ZFP36 activity. This could be achieved without overexpressing *Zfp36* because ZFP36 autoregulates its expression via interaction with adenylate/uridylate-rich elements in the 3′ UTR of its own mRNA (48). Phosphorylated ZFP36 protein is more stable and accumulates as an inactive form in cells (41). Protein phosphatase 2A (PP2A), a ubiquitously expressed serine-threonine phosphatase (49), can dephosphorylate ZFP36, thereby promoting the degradation of inflammatory mRNAs (50). The best-known PP2A-activating drug is the sphingosine analog FTY720 (51) also called fingolimod (brand name Gilenya). FTY720 is an FDA-approved immunosuppressant widely used in patients with relapsing forms of multiple sclerosis (25). Both FTY720 and its primary metabolite, the phosphorylated form of FTY720, known as FTY720-phosphate (FTY720-P), can activate PP2A (51). Therefore, FTY720 can enhance ZFP36 activity by activating PP2A.

To investigate whether FTY720 induces ZFP36 dephosphorylation, thereby enhancing ZFP36 activity, we treated HUVECs with FTY720 and LPS, a strong inducer of ZFP36 expression (52, 53). ZFP36 has multiple phosphorylation sites that are critical for its regulation and function (54). Western blot results showed that ZFP36 was phosphorylated at multiple sites when treated with LPS alone (Supplemental Figure 16A, lane 2). When cotreated with LPS and FTY720, ZFP36 underwent sequential dephosphorylation, shifting from a multisite phosphorylated state to a monophosphorylated form and eventually to a nonphosphorylated state (Supplemental Figure 16, A–C). This suggests that FTY720 promotes ZFP36 dephosphorylation, thereby enhancing its activity.

**Figure 6:**
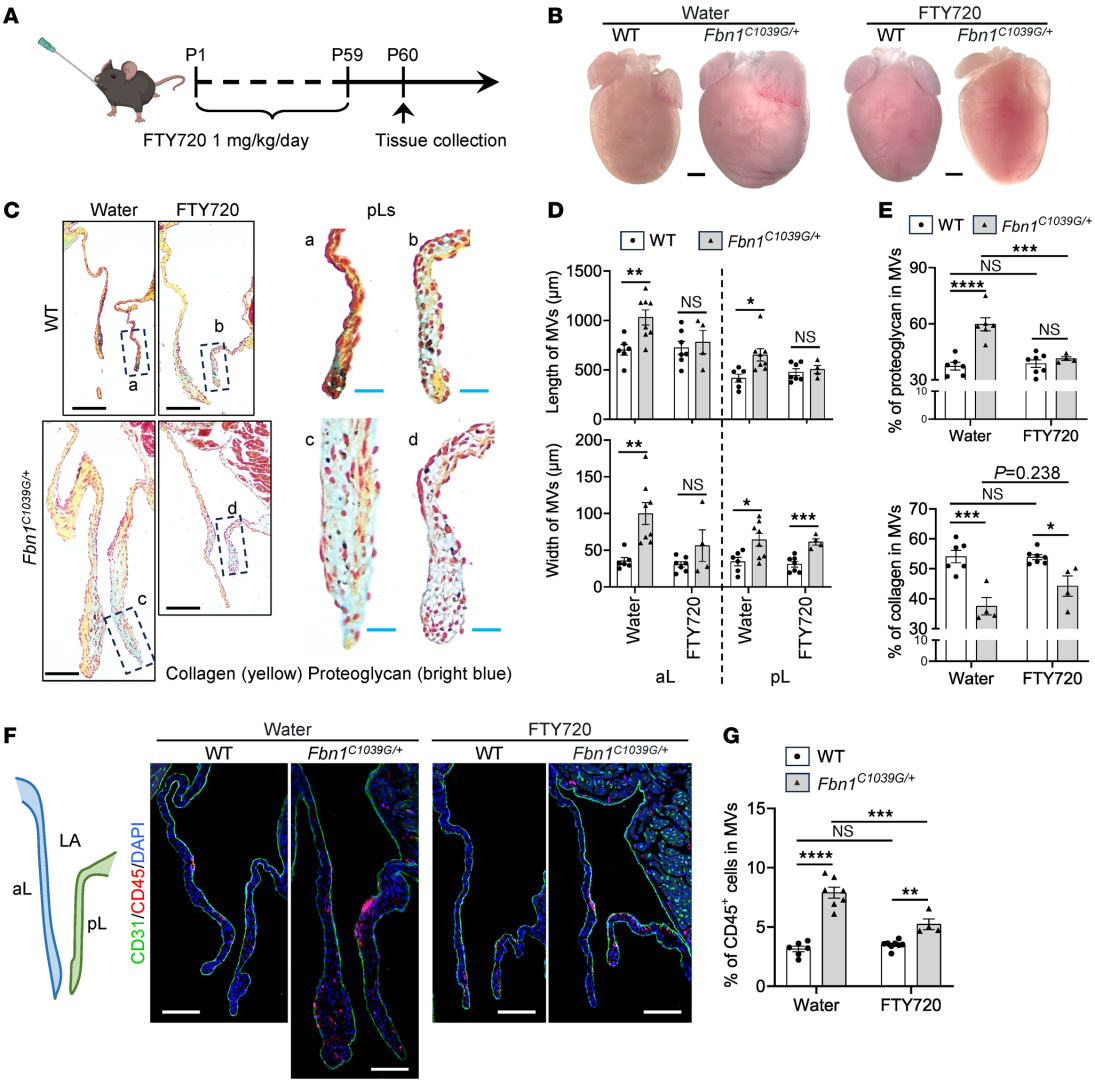
FTY720 rescues MDMV phenotypes in *Fbn1* mutant mice. (**A**) Timeline of FTY720 treatment. (**B**) Representative hearts collected at P60. Scale bars: 1 mm. (**C**) Representative images showing MVs by Movat pentachrome staining. Black and blue scale bars: 100 and 20 μm, respectively. (**D**) Quantification of length (upper panel) and width (lower panel) of the MV leaflets based on the data shown in **C**. Data are mean ± SEM, unpaired 2-tailed Student’s *t* test, each symbol represents 1 mouse. *N* = 4-8 per group, male/female = 3:3, 6:2, 4:3, and 2:2 in the WT-water, *Fbn1*-water, WT-FTY720, and *Fbn1*-FTY720 groups, respectively. *P < 0.05, **P < 0.01, ***P < 0.001. (**E**) Quantification of the average percentage of proteoglycan (upper panel) and collagen (lower panel) in both MV leaflets. Data are mean ± SEM, ordinary 1-way ANOVA test, each symbol represents 1 mouse. For percent proteoglycan, *N* = 4-7 per group, male/female = 3:3, 3:3, 4:3, and 2:2 in the 4 groups, respectively; for percent collagen, *N* = 4-7, male/female = 3:3, 2:2, 4:3, and 2:2 in these groups, respectively. *P < 0.05, ***P < 0.001, ****P < 0.0001. (**F**) Schematic graph and representative confocal images show CD45^+^ immune cells in MVs. Scale bar: 100 μm. LA, left atrium. (**G**) Quantification of the percentage of CD45^+^ cells in both MV leaflets. Data are mean ± SEM, ordinary 1-way ANOVA, each symbol represents 1 mouse. *N* = 4-8, male/female = 3:3, 5:2, 4:4, and 3:1 in the 4 groups, respectively. **P < 0.01, ***P < 0.001, ****P < 0.0001.

To examine whether FTY720 rescues MDMV phenotypes in *Fbn1* mutant mice, we treated WT and *Fbn1* mutant mice with 1 mg/kg FTY720 by daily oral gavage from P1 to P59 and collected their MVs at P60 for analysis (Figure 6A). A significant decline in body weight was observed in both WT and *Fbn1* mutant mice treated with FTY720 compared with water-treated groups, but there was no significant difference in body weight between WT and *Fbn1* mutant mice under FTY720 treatment at P60 (Supplemental Figure 17, A and B). Male and female mice exhibited comparable body weight changes after FTY720 administration (Supplemental Figure 17A). At P60, water-treated *Fbn1* mutant mice had larger hearts than their WT littermates, especially in the ventricles and left atrium (Figure 6B), indicating dilated cardiomyopathy, as previously reported in adult *Fbn1* mutant mice (55) and a patient with MFS (56). The size and morphology of the hearts were similar between FTY720-treated WT and *Fbn1* mutant mice (Figure 6B). Movat pentachrome staining showed that water-treated *Fbn1* mutant mice had significantly thickened and elongated MV leaflets (both aLs and pLs), accompanied by increased proteoglycans and decreased collagen in MVs (Figure 6, C–E), features characteristic of MDMV. WT MVs did not exhibit noticeable morphological changes between water and FTY720 treatment groups (Figure 6, C–E). FTY720 treatment significantly reduced MV length and aL width in *Fbn1* mutant mice, making them comparable with WT mice, although *Fbn1* mutant pLs remained thicker than WT pLs (Figure 6, C and D). Additionally, proteoglycan levels in *Fbn1* mutant MVs decreased to levels similar to those in WT MVs after FTY720 treatment (Figure 6, C and E). There was also a trend toward increased collagen in *Fbn1* mutant MVs after FTY720 treatment, but the increase was much smaller than in WT MVs (Figure 6, C and E). Echocardiography further revealed improved cardiac function in FTY720-treated *Fbn1* mutant hearts, with increased ejection fraction and fractional shortening comparable with those of WT (Supplemental Figure 17, C and D). These findings suggest that FTY720 prevents MDMV and helps preserve MV integrity, thereby improving cardiac function in *Fbn1* mutant mice.

Next, we quantified the number of CD45^+^ immune cells in MVs by IHC (Figure 6F). *Fbn1* mutant mice had more CD45^+^ cells in MVs than their WT littermates when treated with water at P60. No significant difference was observed in the number of CD45^+^ cells between WT mice treated with water and those treated with FTY720 (Figure 6, F and G). Although the number of CD45^+^ cells in *Fbn1* mutant MVs treated with FTY720 was higher than in MVs of FTY720-treated WT mice, CD45^+^ cells were significantly fewer in MVs of *Fbn1* mutant mice treated with FTY720 than in MVs of *Fbn1* mutant mice treated with water. These results indicate that FTY720 reduces inflammation in MVs of *Fbn1* mutant mice.

To further confirm that the rescue effect of FTY720 on MDMV phenotypes in *Fbn1* mutant mice depends on ZFP36, we examined EC-*Zfp36*-KO MVs following FTY720 treatment (Supplemental Figure 15E). The differences in proteoglycan deposition and inflammatory infiltration between control and EC-*Zfp36*-KO MVs remained unchanged with FTY720 treatment compared with the water-treated group (Supplemental Figure 15, F–H), indicating that the therapeutic effect of FTY720 was diminished in EC-specific *Zfp36* KO MVs. These data provide strong support for the mechanistic link between FTY720 treatment and *ZFP36* in vivo.

FTY720 treatment is known to modulate the trafficking of T and B lymphocytes between blood and secondary lymphoid organs (57), leading to a decrease in T cells in blood (58) and lymphoid organs (59) and an increase in B cells in lymph nodes (57) and spleen (58). This prevents lymphocyte migration to allogeneic graft tissue and other sites of inflammation (60). To test whether FTY720 inhibits inflammation in *Fbn1* mutant MVs by regulating lymphocyte trafficking, we examined its effect on T and B cells in the blood, spleen, and lymph nodes from WT and *Fbn1* mutant mice at P16 by flow cytometry (Supplemental Figure 18, A–F). Consistent with previous findings, we observed a significant reduction in T cell numbers in blood, lymph nodes, and spleen after FTY720 treatment in both WT and *Fbn1* mutant mice compared with water-treated WT mice (Supplemental Figure 18, A–C). B cell numbers exhibited a downward trend in the blood but were significantly increased in the lymph nodes and spleen in FTY720-treated mice compared with water-treated mice (Supplemental Figure 18, D–F). Notably, there was no significant difference in lymphocyte counts in blood, spleen, and lymph nodes between WT and *Fbn1* mutant mice treated with FTY720 (Supplemental Figure 18, A–F), indicating that the protective effect of FTY720 on *Fbn1* mutant MVs might not be due to its lymphocyte-modulating function. Overall, these data demonstrate that FTY720 rescues the MDMV phenotypes in *Fbn1* mutant mice by suppressing inflammation within MVs.

### FTY720 enhances lymphatic function and the VEC barrier in MVs of Fbn1 mutant mice.

**Figure 7:**
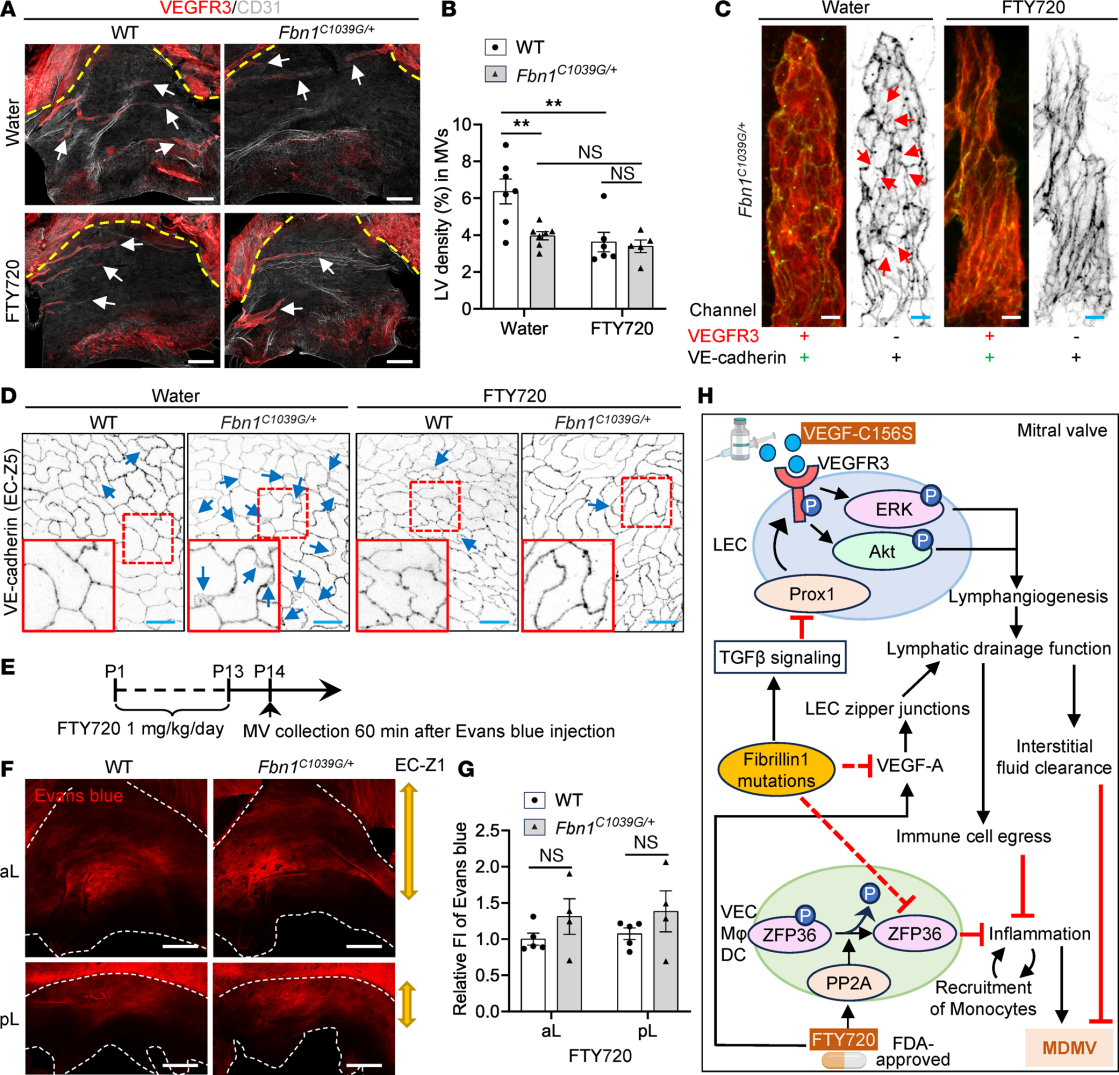
FTY720 promotes lymphatic function and the VEC barrier in MVs of *Fbn1* mutant mice. (**A**) Representative confocal images show LVs (arrows) in aLs at P60. Scale bars: 200 μm. (**B**) Quantification of LV density in both leaflets. Data are mean ± SEM, ordinary 1-way ANOVA test, each symbol represents 1 mouse. *N* = 5-7, male/female = 4:3, 3:4, 4:2, and 3:2 in the 4 groups, respectively. **P < 0.01. (**C**) Representative confocal images show LEC junctions in lymphatic capillaries of MVs. Arrows indicate disrupted junctions. Scale bars: 10 μm. (**D**) Representative confocal images show VEC junctions at EC zone 5. Arrows indicate reticular adherens junctions. Scale bars: 20 μm. (**E**) Timeline of treatments for **F** and **G**. (**F**) Evans blue permeability assay in MVs. White dashed lines outline the regions of MV leaflets. EC-Z1, EC zone 1. Scale bars: 200 μm. (**G**) Quantification of fluorescence intensity (FI) of Evans blue in the interstitium beneath EC zone 1. Data are mean ± SEM, unpaired 2-tailed Student’s *t* test, each symbol represents 1 mouse. *N* = 4-5, male/female = 2-3:2. (**H**) Schematic summary of VEGF-C156S and FTY720 rescuing the MDMV phenotypes in *Fbn1* mutant mice. In LECs of the *Fbn1* mutant MVs, enhanced TGF-β signaling suppresses Prox1 expression and downregulates the VEGFR3 pathway. VEGF-C156S promotes lymphangiogenesis and lymphatic drainage, facilitating immune cell egress and interstitial fluid clearance, thereby inhibiting inflammation and preventing MDMV. Additionally, the antiinflammatory modulator ZFP36 is downregulated in VECs, macrophages, and DCs. The FDA-approved compound FTY720 activates PP2A, which dephosphorylates and thereby activates ZFP36, suppressing inflammation and preventing MDMV. FTY720 also facilitates zippering of LEC junctions via VEGF-A upregulation, thereby promoting efficient lymphatic drainage.

We further analyzed lymphatics in WT and *Fbn1* mutant MVs at P60 after FTY720 treatment using whole-mount immunostaining for VEGFR3 (Figure 7A). As in earlier ages, *Fbn1* mutant mice had fewer LVs in MVs than their WT littermates at P60 when treated with water. FTY720 administration reduced LV density in WT MVs but did not affect *Fbn1* mutant LVs (Figure 7, A and B), indicating that FTY720 inhibits lymphatic growth in WT MVs. Since FTY720 is known to impede sphingosine 1-phosphate (S1P) signaling (61), which promotes lymphangiogenesis (62), FTY720 treatment likely blocks S1P signaling in the MVs. As a structural analog of sphingosine, FTY720-P — the active primary metabolite of FTY720 — binds with high affinity to 4 of the 5 S1P receptors, S1PR1, S1PR3, S1PR4, and S1PR5 (63), leading to their internalization and degradation and thereby inhibiting S1P signaling (64). Additional analysis of the scRNA-seq datasets used above (Figure 4C) identified *S1pr1* as the main receptor expressed in the EC cluster (Supplemental Figure 19A), with no significant difference in *S1pr1* levels between WT and *Fbn1* mutant ECs (Supplemental Figure 19B). We noted earlier that a small number of LECs were found in the EC cluster (Supplemental Figure 11), and LECs express S1PR1 (62). Therefore, FTY720 likely regulates lymphatic growth in WT MVs by downregulating S1PR1 on LECs. We therefore tested this hypothesis by performing whole-mount S1PR1 immunostaining on P60 mouse MVs after FTY720 treatment. FTY720 significantly reduced S1PR1 expression in LECs of WT MVs, whereas no such change was observed in LECs of *Fbn1* mutant MVs (Supplemental Figure 20, A and B). This aligns with the observation that FTY720 reduced LV density in WT MVs but not in *Fbn1* mutant MVs (Figure 7, A and B).

Although FTY720 treatment did not enhance LV growth in *Fbn1* mutant MVs, more continuous zipper LEC junctions were observed in their lymphatic capillaries compared with those treated with water (Figure 7C). This may be because FTY720 can upregulate VEGF-A (65), which promotes the zippering of lymphatic VE-cadherin junctions (66) and opposes junction opening (67) in initial LVs. Without FTY720 treatment, our analysis of published scRNA-seq datasets (38) showed that *Vegfa* was expressed in many cells in WT MVs (Supplemental Figure 21A), indicating a high level of VEGF-A that could cause zipper-like LEC junctions in lymphatic capillaries (Figure 2C). It was previously reported that knockdown of *fbn1* by FBN1 morpholino decreases *vegfa* mRNA expression in zebra fish (68), and our data also showed that *Vegfa* expression was significantly lower in the total cells of *Fbn1* mutant MVs at P30 (Supplemental Figure 21A), which could lead to disrupted LEC junctions in the lymphatic capillaries of *Fbn1* mutant MVs (Figure 2C). No significant difference was found in *Vegfc* levels in the total cells of MVs between WT and *Fbn1* mutants (Supplemental Figure 21B), suggesting that *Fbn1* mutation does not affect VEGF-C levels in MVs.

VEC junctions were also evaluated at P60 across different EC zones because *S1pr1* is enriched in ECs (Supplemental Figure 19A), and compromised VEC junctions can lead to MDMV (16). Under water treatment, *Fbn1* mutant MVs showed increased reticular adherens junctions in VECs, particularly in EC zones 1, 4, 5, and 6 (Figure 7D and Supplemental Figure 22), indicating impaired VEC barrier (16). FTY720 administration reduced the number of reticular adherens junctions in VECs of *Fbn1* mutant MVs (Figure 7D and Supplemental Figure 22), suggesting that FTY720’s protective effect on the VEC barrier lowers immune cell accumulation in *Fbn1* mutant MVs.

To determine whether FTY720 treatment can influence VEC permeability and lymphatic drainage function in *Fbn1* mutant MVs, Evans blue was administered at P14 after FTY720 treatment from P1 to P13 (Figure 7E). In both aLs and pLs of *Fbn1* mutant MVs, lymphatic drainage function was significantly restored and comparable with WT MVs (Figure 7, F and G), indicating that FTY720 protects against the accumulation of excess interstitial fluid in *Fbn1* mutant MVs. Together, these findings demonstrate that FTY720 restores the VEC barrier and lymphatic drainage in *Fbn1* mutant MVs.

## Discussion

Lymphatic dysfunction, such as defective LV growth and disrupted LEC junctions, contributes to the development of various cardiovascular diseases (69). Although MDMV is a hallmark of MFS, the pathological processes underlying MDMV in individuals with MFS, including LV abnormalities and inflammatory macrophages, remain unclear. Here, we demonstrate that postnatal LV development, LEC junction formation, and lymphatic drainage in MVs are severely impaired in *Fbn1* mutant mice, a mouse model of MFS. By treating *Fbn1* mutant mice with VEGF-C156S to promote lymphatic growth, we demonstrate that MDMV can be rescued. Our findings also reveal that VECs of *Fbn1* mutant MVs are in a proinflammatory state, with decreased expression of the antiinflammatory factor *Zfp36*, and that the MDMV phenotype, along with lymphatic dysfunction, can be restored by treatment with the immunosuppressant and ZFP36 agonist FTY720. Together, these results suggest that enhancing lymphatic function with VEGF-C156S and FTY720 may be a promising therapeutic approach for treating MFS-associated MDMV.

### LV development in the murine MV.

Cardiac LV development in the mouse embryo begins with lymphatic sprouting from extracardiac ventral regions near the outflow tract at E12.5 (70). Expansion and network formation then proceed from the base of the heart toward the apex until birth, followed by postnatal maturation with fully developed lymphatic networks by P15 (70). However, LV development in the murine MV, as well as in other species including humans, has not been thoroughly studied, although our recent research identified the precise location of LVs in the adult murine MV (16). In this study, whole-mount immunostaining of WT MVs showed that VEGFR3^+^ LVs expanded postnatally into the mitral annulus at P3, sprouting from the triangular region above the anterior commissure of the MV leaflets (Figure 1). LV sprouting in both the anterior and posterior MV leaflets continued until P14, with significant LEC proliferation observed during this period (Figure 1D). This study offers direct insights into postnatal lymphangiogenesis in the murine MV.

### Suppression of lymphangiogenesis in Fbn1 mutant MVs.

While examining LV formation in WT MVs, we also observed the inhibition of lymphatic proliferation and outgrowth in *Fbn1* mutant MVs at P14, along with abnormal MV enlargements (Figure 1). Our findings align with evidence that the *Fbn1* mutation causes excessive TGF-β signaling (15), which is known to suppress lymphangiogenesis (12–14). Impaired LV formation was also detected in the ventricles of *Fbn1* mutant hearts (Supplemental Figure 7). Since inflammatory macrophages contribute to the progression of MDMV in MFS through pathological ECM remodeling (20, 47), the widespread infiltration of macrophages in *Fbn1* mutant MVs compared with WT MVs could, at least in part, be due to impaired lymphatic drainage, thereby increasing chronic inflammation and disease progression. Disrupted cardiac lymphatics similarly lead to defective clearance of the acute inflammatory response after myocardial infarction (71). Recently, we demonstrated that LEC-specific deletion of *Foxc1* and *Foxc2* causes similar structural and ECM abnormalities in adult murine MVs (16). Importantly, in this study, we demonstrate that the expansion of LVs in *Fbn1* mutant MVs, achieved through VEGF-C156S treatment, rescues the MDMV phenotype in these mice. These findings support the notion that lymphatic function is crucial for maintaining MV integrity, including ECM organization, thereby preventing MDMV. Our scRNA-seq reanalysis showed that the EC cluster is the primary source of *Flt4* (VEGFR3) expression in MVs. Very few VEGFR3^lo^ cells were observed in other MV cell types (Supplemental Figure 23, A and B). Because the EC cluster includes VECs and LECs (Supplemental Figure 11), further investigation of the effects of VEGF-C156S on VECs is warranted.

### Downregulation of Zfp36 in proinflammatory VECs of Fbn1 mutant MVs.

Our secondary analysis of published scRNA-seq data from P30 WT and *Fbn1* mutant MVs (38) identified *Zfp36* as a critical regulator of MDMV progression in MFS. ZFP36 is an mRNA-binding protein that acts as an antiinflammatory factor in vascular inflammation and atherosclerosis by promoting the decay of proinflammatory cytokine mRNAs in inflamed ECs and macrophages (24). Global *Zfp36*-knockout mice show thickening of the MV, along with inflammatory cell infiltration (43), while ZFP36 levels in the circulatory system are linked to MV prolapse pathogenesis (44). FTY720 is an FDA-approved immunosuppressive drug for multiple sclerosis and functions as an antagonist targeting S1P receptors (72). It also stimulates ZFP36 through PPA2 activation (41, 73). Therefore, treatment with FTY720 likely exerts its effects by both inhibiting S1P signaling and activating ZFP36 in the MVs, thereby restoring lymphatic cell junctions and drainage function and reducing immune cell infiltration.

In conclusion, this work provides direct evidence of the lymphatic system’s role in the pathophysiology of MDMV in *Fbn1* mutant MFS, along with critical mechanistic insights into the complex interactions among proinflammatory VECs, immune cell infiltration, and lymphatic drainage under excessive TGF-β signaling (Figure 7H). Future research should explore how lymphatic function and immune modulation can be targeted therapeutically to prevent MDMV.

## Methods

### Sex as a biological variable.

Our study examined male and female animals, and similar findings are reported for both sexes.

### Animal husbandry and treatment.

*Fbn1^C1039G/+^* (74) and *Prox1-eGFP* (26) mice were used. *Fbn1^C1039G/+^* mice, harboring a Cys1041Gly missense mutation (previously identified as Cys1039Gly) in the *Fbn1* gene similar to the mutation that causes classic manifestations of MFS in humans (Cys1039Tyr), were purchased from The Jackson Laboratory (strain 012885). *Prox1-eGFP* mice were provided by Young-Kwon Hong (University of Southern California). *Fbn1^C1039G/+^/Prox1-eGFP* mice were generated by crossing *Fbn1^C1039G/+^* female mice with *Prox1-eGFP* male mice. For embryonic tissue collection, timed pregnant mice were set up, and E0.5 was defined as the day when a vaginal plug was detected after mating. EC-specific *Zfp36* mutant mice (*Cdh5-Cre^ERT2^ Zfp36^fl/fl^*, referred to as EC-*Zfp36*-KO) were generated previously (45) by crossing *Cdh5-Cre^ERT2^* male mice (75) with *Zfp36*-floxed female mice (*Zfp36^fl/fl^*) (42). Their littermates, *Zfp36^fl/fl^* mice, were used as controls. Genotyping of mice was performed by Transnetyx Inc. Detailed information about drug treatments is described in Supplemental Methods.

### Tissue collection and whole-mount immunostaining.

Mouse heart collection and whole-mount immunostaining of MVs and embryonic hearts were performed as previously described (16). For hearts collected postnatally, whole-mount staining was performed using the iDISCO (76) and Adipo-Clear (77) methods, as previously outlined, with modifications. WM staining of the small intestine was performed as previously described (78). Detailed information regarding tissue collection, whole-mount staining of MVs, hearts, small intestine, diaphragm, and dorsal ear skin is described in Supplemental Methods. The antibodies used are listed in Supplemental Table 5.

### Wet/dry weight ratio in hearts.

Detailed information regarding wet/dry weight ratio in hearts is described in Supplemental Methods.

### Movat pentachrome staining and Masson’s trichrome staining.

Detailed information about Movat pentachrome/Masson’s trichrome staining is included in Supplemental Methods.

### IHC staining.

IHC on 4 μm paraffin sections or 10 μm frozen sections was performed as previously described (78). Sections incubated in blocking buffer without primary antibodies served as negative controls. The antibodies used are listed in Supplemental Table 5.

### RNAscope costained with IHC.

RNAscope (RNA in situ hybridization analysis) followed by IHC staining on freshly cut paraffin sections (4 μm) was performed as previously reported (16). Detailed information is listed in Supplemental Methods.

### Evans blue permeability assay.

The Evans blue permeability assay was performed as previously reported (16). Detailed information is provided in Supplemental Methods.

### Imaging and quantification.

Detailed information about imaging and quantification is listed in Supplemental Methods.

### scRNA-seq data analysis.

A secondary analysis of the published scRNA-seq data (38) was performed. Raw scRNA-seq data were accessed through the Gene Expression Omnibus (GEO) website (GSE261874). Detailed information about scRNA-seq analysis is provided in Supplemental Methods.

### Flow cytometry analysis.

Flow cytometry was performed on MV samples (79), as well as blood, spleen, and lymph node samples (58, 59, 79) as previously described. Detailed information is provided in Supplemental Methods. Antibodies used are listed in Supplemental Table 6.

### FTY720 and LPS treatment of HUVECs.

HUVECs, isolated from a human donor (80), were used between passages 7 and 10. Detailed information about cell culture, treatment, Western blotting, and analysis is provided in Supplemental Methods.

### Echocardiography.

Echocardiography was performed as previously described (81) to evaluate cardiac function. Detailed information is provided in Supplemental Methods.

### Statistics.

For quantification, statistical analysis was performed using GraphPad Prism 8.0 (GraphPad Software). In vivo data were analyzed using an unpaired 2-tailed Student’s *t* test or an ordinary 1-way ANOVA following normality testing. For the in vitro study, a paired 2-tailed Student’s *t* test was used. Data are presented as mean ± SEM of representative experiments from at least 3 biological replicates unless indicated otherwise. *P* values < 0.05 or adjusted *P* values < 0.05 (for scRNA-seq data) were considered statistically significant.

### Study approval.

All experimental protocols and procedures in this study were approved by the IACUC at Northwestern University.

### Data availability.

The scRNA-seq data reanalyzed in this study were acquired from a published study (38) via GEO under the accession code GSE261874. Values for all data points in graphs are reported in the Supporting Data Values file.

## Author contributions

C Tan contributed to conceptualization, formal analysis, investigation, methodology, project administration, supervision, validation, visualization, and writing (original draft, review, and editing). ZR contributed to software development, formal analysis, data curation, resources, and visualization. XL contributed to methodology, validation, formal analysis, investigation, resources, and visualization. ZDG contributed to methodology, investigation, and validation. SS contributed to methodology, validation, formal analysis, investigation, and visualization. SK, PJ, and C Tang contributed to investigation. MLIA contributed to conceptualization, investigation, funding acquisition, and resources. TK contributed to conceptualization, funding acquisition, project administration, supervision, and writing (original draft, review, and editing).

## Conflict of interest

The authors have declared that no conflict of interest exists. Patent information: “Methods and Compositions for the Treatment of Marfan Syndrome,” 63/872,860, filed 8/29/2025. Inventors: TK and C Tan. Applicant: Northwestern University.

## Funding support

This work is the result of NIH funding, in whole or in part, and is subject to the NIH Public Access Policy. Through acceptance of this federal funding, the NIH has been given a right to make the work publicly available in PubMed Central.

- NIH (R01HL159976 and R01EY034740 to TK).
- National Cancer Institute, Cancer Center support grant (P30 CA060553) awarded to the Robert H. Lurie Comprehensive Cancer Center (NIH 1R01HL178787).
- Leducq 21CVD03 (to MLIA).

## Supplementary Material

Supplemental data

Unedited blot and gel images

Supplemental video 1

Supplemental video 2

Supporting data values

## Version 1 06/11/2026

In-Press Preview

## Version 2 08/03/2026

Electronic publication
