# Supplementary material for: Lymphatic dysfunction and ZFP36 deficiency contribute to myxomatous valve degeneration in Marfan syndrome mice
Source: J Clin Invest. 2026 Jun 2;136(15):e195507. doi: 10.1172/JCI195507 (PMC13430018; doi:10.1172/JCI195507)
Supplement: Supplemental data [file jci-136-195507-s035.pdf]

SUPPLEMENTAL MATERIAL

Lymphatic dysfunction and ZFP36 deficiency contribute to myxomatous valve degeneration in Marfan Syndrome mice

Can Tan[1], Ziyou Ren[2], Shreya Kurup[1], Xianpeng Liu[3], Zhi-Dong Ge[4], Shodai Suzuki[5], Pritika Jakka[1], Cheryl Tang[1], M. Luisa Iruela-Arispe[4], and Tsutomu Kume[1]

[1]Feinberg Cardiovascular and Renal Research Institute, Department of Medicine, Feinberg School of Medicine, Northwestern University, Chicago, Illinois, USA

[2]Department of Dermatology, Feinberg School of Medicine, Northwestern University, Chicago, Illinois, USA

[3]Division of Thoracic Surgery/Canning Thoracic Institute, Feinberg School of Medicine, Northwestern University, Chicago, Illinois, USA

[4]Cardiovascular-Thoracic Surgery and the Heart Center, Stanley Manne Children's Research Institute, Ann & Robert H. Lurie Children's Hospital of Chicago, Departments of Pediatrics, Surgery, and Pathology, Feinberg School of Medicine, Northwestern University, Chicago, Illinois, USA

[5]Department of Cell and Development Biology, Feinberg School of Medicine, Northwestern University, Chicago, Illinois, USA

Correspondence to:

Tsutomu Kume, PhD, Feinberg Cardiovascular and Renal Research Institute, Department of Medicine, Feinberg School of Medicine, Northwestern University, 303 E. Superior Street, Chicago, IL, 60611, USA

E-mail: t-kume@northwestern.edu

Contents

Supplemental Methods 4
Supplemental Figures 17
Supplemental Figure 1 17
Supplemental Figure 2 19
Supplemental Figure 3 20
Supplemental Figure 4 21
Supplemental Figure 5 23
Supplemental Figure 6 24
Supplemental Figure 7 25
Supplemental Figure 8 26
Supplemental Figure 9 27
Supplemental Figure 10 28
Supplemental Figure 11 30
Supplemental Figure 12 31
Supplemental Figure 13 32
Supplemental Figure 14 33
Supplemental Figure 15 34
Supplemental Figure 16 36
Supplemental Figure 17 37
Supplemental Figure 18 38
Supplemental Figure 19 39
Supplemental Figure 20 40
Supplemental Figure 21 41
Supplemental Figure 22 42
Supplemental Figure 23 43
Video Legends 44
Video 1 44
Video 2 44
Supplemental Tables 46
Supplemental Table 1 46
Supplemental Table 2 47

Supplemental Table 3 48
Supplemental Table 4 49
Supplemental Tabel 5 50
Supplemental Table 6 51

Supplemental Methods

Drug treatments

For EdU staining, mice were treated with 50 mg/kg of 5-Ethynyl-2′-deoxyuridine (EdU, Santa Cruz Biotechnology, sc-284628) in PBS at a concentration of 10 mg/mL by intraperitoneal (i.p.) injection at postnatal day 13 (P13), followed by heart collection 24 h after the treatment (P14). For VEGF-C156S treatment, neonatal mice were treated with 0.1 mg/kg of recombinant human VEGF-C (Cys156Ser) protein (R&D Systems, 752VC025CF, dissolved in PBS at 10 μg/mL) by intraperitoneal (i.p.) injection at P3, P6, P9 and P12. Hearts were collected at P14 or at 6 weeks of age for further analysis. Mice treated with PBS at the same time points were used as controls. For FTY720 treatment, mice were treated with 1 mg/kg of FTY720 (Sigma, SML0700-25MG, dissolved in water) or water (as control) by oral gavage once daily from P1 to P59. Hearts were collected at P60. To induce *Zfp36* deletion in ECs of EC-*Zfp36*-KO mice, tamoxifen was administered to the neonates at P1, P3, and P5 by gavage (5 μL) at a dose of 20 mg/mL. Mitral valves were collected at P60.

Tissue collection

Postnatal and adult mouse hearts were collected at specific time points for histological analysis as previously described (16). Briefly, transcardiac perfusion was performed on mice with cold PBS (plus 10 U/mL heparin) followed by 4% paraformaldehyde (PFA) after anesthesia. The hearts were then dissected and post-fixed in 2% PFA (for frozen or whole-mount samples) or 4% PFA (for paraffin-embedded samples) at 4°C for overnight (O/N). The fixed hearts were then processed to OCT- or paraffin-embedded samples. For embryonic heart collection, the pregnant dams were euthanized by CO_2. The embryos

were collected, tails were cut for genotyping, and the embryonic hearts were dissected and fixed in 2% PFA at 4°C for O/N, followed by whole-mount (WM) immunostaining.

Whole-mount immunostaining for mitral valves

WM immunostaining of mitral valves (MVs) was performed as previously described (16). Briefly, after the fixation of the heart, the anterior and posterior MV leaflets together with their connected mitral annulus and papillary muscles were dissected and processed to the staining. Tissues were permeabilized in PBST (0.3% Triton X-100 in PBS) for 1h at 4°C (or alternatively in cold acetone for 20 min at -20°C), blocked in blocking buffer (5% donkey serum, 0.5% BSA, 0.3% Triton X-100, 0.1% NaN_3 in PBS) for 2 h at 4°C, incubated with primary antibodies (Supplemental Table 5) diluted in the blocking buffer for 2~3d at 4°C. Samples were washed with PBST several times, followed by incubation with fluorochrome-conjugated secondary antibodies (Supplemental Table 5) diluted in the blocking buffer for 1~2 days at 4°C. The samples were washed again with PBST several times, post-fixed with 4% PFA, cleared with FocusClear (CelExplorer Labs, FC-101) and flat-mounted on slides with imaging spacer (Electron Microscopy Sciences, 70327-9S) in the mounting medium (Epredia, TA-030-FM). Similar protocol was applied to the negative control samples, but they were incubated in the blocking buffer in the step of primary antibody treatment. For EdU staining, MVs from the mice treated with EdU were first stained with primary and secondary antibodies. After post-fixation with 4% PFA and before tissue clearing, MVs were washed with 3% BSA in PBS, incubated in Click-iT™ reaction cocktail prepared using Click-iT™ EdU Imaging Kit (Thermo Fisher Scientific, C10086) for 1.5 h at room temperature (RT), followed by wash with 3% BSA in PBS.

Whole-mount immunostaining for hearts

For hearts collected at E12.5 (Figure 1A) and left ventricular tissue collected at P14 (Supplemental Figure 7B), WM staining was performed using the same protocol as mentioned above for the MV samples. At the step of mounting, several layers of imaging spacers (Grace Bio-Labs, GBL654006) were used. For whole hears collected postnatally, WM staining was performed using iDISCO (76) and Adipo-Clear (77) methods as previously described with modifications. Briefly, after fixation, whole hearts (for Supplemental Figure 7A) or half hearts (for Supplemental Figure 1D) were dehydrated in methanol/B1n buffer (Glycine 0.3 M, Triton X-100 0.1%, 0.01% NaN_3 in H_2O, pH 7.0) series (20%, 40%, 60% supernatant, 80% supernatant, 30 min each and 100% methanol, 1h) at 4°C, delipidated with 66% dichloromethane (DCM, Sigma, 270997) in methanol for O/N at 4°C, washed with 100% methanol for 30 min at 4°C, bleached with 5% hydrogen peroxide in methanol for 5 h or O/N at 4°C, rehydrated in methanol/B1n buffer series (100%, 80%, 60%, 40%, 20%, B1n buffer) for 30 min each at 4°C, washed with PtxwH solution (0.1% Triton X-100, 0.05% Tween 20, 2 μg/mL heparin, 0.01% NaN_3 in PBS) for 2 h at RT, blocked in blocking solution (PtxwH/10% donkey serum solution) for O/N at RT, incubated in primary antibodies (Supplemental Table 5) in PtxwH/3% donkey serum solution for 2 days at RT, washed with PtxwH solution several times, incubated in secondary antibodies (Supplemental Table 5) in PtxwH/3% donkey serum solution for 1~2 days at RT, washed with PtxwH solution several times, post-fixed in 4% PFA for 30 min at RT, washed with PBS, dehydrated in methanol/H_2O series (25%, 50%, 75%, 20 min each and 100% methanol, 20 min by 3 times) at RT, cleared in ethyl cinnamate (Eci, Sigma, 8002380250) for 1 h at RT, and stored in Eci at RT for further imaging.

Whole-mount staining for small intestine, diaphragm and dorsal ear skin

Small intestine, diaphragm, and dorsal ear skin were harvested at 6 weeks of age. WM staining of small intestine was performed as previously described (78). Briefly, distal jejunum was dissected and fixed in 2% PFA for O/N. A small piece of intestine tissue (about 5 mm in width) was used for immunostaining. WM staining of the diaphragm and dorsal ear skin was performed using the same methods as described above for mitral valves and postnatal hearts, respectively.

Wet/dry ratio in hearts

Mice at 3~6 months of age were euthanized by CO_2. The chests were opened, the hearts were dissected, and the attached fat, thymus, arteries, and veins were removed. The walls of the left and right atria and ventricles were opened, and the remaining blood in each heart chamber was removed using Kimwipes. The hearts were weighed before and after drying in a 37 °C incubator for 4 days to obtain wet and dry weights. The heart's wet/dry ratio was used to evaluate the severity of cardiac edema by dividing the wet weight by its dry weight.

Movat Pentachrome staining and Masson's Trichrome staining

Movat Pentachrome and Masson's Trichrome staining (blue color only for quantifying the collagen percentage in MVs) were performed on 4-µm paraffin sections of the hearts according to the manufacturers' instructions (Scytek Laboratories, MPS2; and Thermo Scientific, #87019).

RNAscope co-stained with immunohistochemistry staining

RNAscope (RNA *in situ* hybridization analysis) followed by immunohistochemistry (IHC)

staining on freshly cut paraffin sections (4 μm) was performed as previously described (16). RNAscope^{TM} Multiplex Fluorescent Reagent Kit v2 (#323100), RNAscope^{TM} mRNA probes (mouse *Zfp36*: # 888831; mouse positive control probe: #320881, *Ubc*-C3 was selected; negative control probe: bacteria *dapB*, #320871) from Advanced Cell Diagnostics, Inc., and Alexa Fluor™ 568 Tyramide conjugates (ThermoFisher, B40956) were used according to the manufacturers' instructions. After RNAscope staining, IHC staining was performed using goat anti-mouse CD206 antibody (R&D Systems, AF2535SP) and donkey anti-goat 488 antibody (ThermoFisher, A-11055), followed by staining with 10 μg/mL Alexa Fluor™ 647-conjugated Isolectin GS-IB4 (IB4) in PBST for 1h at RT, and counterstain with DAPI. The sections were then mounted and ready for imaging.

Evans blue permeability assay

Evans blue permeability assay was performed as previously described (16). Briefly, mice at P14 were treated with 50 mg/kg of Evans blue (Sigma, 206334-10G, 10 mg/mL in PBS) by retro-orbital injection. Sixty minutes after the treatment, cardiac perfusion with cold PBS followed by cold 4% PFA was performed. The MVs were dissected immediately and fixed in 4% PFA at RT for 10 min. After washing with PBS, the MV leaflets were mounted and imaged under a confocal microscope (Evans blue: farRED channel, excitation wavelength 640).

Imaging

The whole heart bright field images were taken under a stereo microscope (Nikon SMZ 745) connected to a camera from Nikon DS-Fi2 (for hearts from E12.5 to P7) or the

camera of Apple iPhone 12 Pro Max (for hearts at P14 and older). Movat Pentachrome and Masson's Trichrome staining images were acquired using a Nikon ECLIPSE T2 Widefield inverted microscope. WM, RNAscope and IHC staining images were acquired using a Nikon A1 Confocal Laser Microscope with the NIS-Elements Viewer software. WM whole heart (P14) immunostaining images were taken using the AxL Cleared Tissue Light Sheet Microscopy System (3i-Intelligent Imaging Innovations). WM half heart (P14) immunostaining images were taken using a LaVision Light Sheet Microscope (Miltenyi Biotec). Images were processed and analyzed with Adobe Photoshop, Fiji (ImageJ) and Imaris Workstation (for 3-D reconstruction).

Quantification

Fiji (ImageJ) software was used for the measurement of length, area, number of cells and fluorescent intensity (FI) of specific markers.

For quantification of lymphatic vessel (LV) density and lymphatic branching points in MV leaflets, Z-stack images acquired using a 10x objective for both whole anterior (aL) and posterior (pL) MV leaflets were analyzed. The area of the LVs (VEGFR3^{+}) and the MV leaflet were measured using ImageJ software, respectively. LV density percentage was determined as the total LV area / total MV leaflet area x 100%. The number of lymphatic branching points per LV area was then counted and calculated.

To quantify the LV density and lymphatic branching points in the left ventricle, four Z-stack images for each whole-mount left ventricle sample were acquired using a 10x objective. The LVs (Prox1-eGFP^{+}VEGFR3^{+}) and ventricular tissue were measured for area using ImageJ software, respectively. For each image, LV density percentage was determined

as the LV area / left ventricular tissue area x 100%. The number of lymphatic branching points per LV area was then counted and calculated. The mean values were calculated based on the data from 4 images for each mouse.

To quantify the thickness of whole-mount stained MV leaflets (Figure 3E, Supplemental Figure 9F, and Supplemental Figure 15D), confocal images for the MVs were acquired using a 10x objective. Three 2-D resliced images for each MV leaflet were obtained by ImageJ via drawing straight lines along the proximal-to-distal direction at the ¼, ½, and ¾ locations on the leaflet. The resliced image showed the cross section of the leaflet (as shown in Figure 3D, Supplemental Figure 9E, and Supplemental Figure 15C). Thickness of the leaflet was measured on the resliced leaflet at 5 points which divided the whole length of leaflet into 6 equal parts. Therefore, average thickness of the leaflet for each sample was calculated based on the data from 15 points (3 resliced images x 5 points).

For quantifying the length and thickness of MV leaflets on paraffin sections (Figure 6D), the lengths of aL and pL were determined by examination of serial sections and by measuring the distance along the center of the leaflet from the valvular root to its tip. The thickness of leaflets was measured at the thickest point perpendicular to the length axis of the leaflet.

For the quantification of percentages of proteoglycan and collagen in aL and pL (Figure 6E), only one color was applied to the heart paraffin sections: bright blue (Alcian Blue solution, pH 2.5) from Movat Pentachrome staining for proteoglycan, or blue (Anline Blue solution) from Masson's Trichrome staining for collagen, respectively. Images were taken for serial sections focusing on the leaflet area. The area of the single color for

proteoglycan (bright blue) or collagen (blue) was measured by thresholding in ImageJ. The area of aL or pL was also measured by selecting the region of aL or pL. The percentage of proteoglycan or collagen in aL or pL was then calculated: % = area of proteoglycan or collagen / area of aL or pL x 100%. Figure 6E displays the average data in both leaflets.

For quantifying the FI of Evans blue in MV leaflets, (Figure 2E and Figure 7G), confocal images of whole-mount MV leaflets were taken under a 10x objective. The EC zone 1 of MV leaflet was selected from the Z-stack confocal image for the measurement of FI of Evans blue. Quantification of FI of Evans blue inside and outside LVs in MVs (Figure 2G) was performed as previously described (16).

To quantify the $CD45^+$ immune cells in MV leaflets (Figure 6G and Supplemental Figure 8E), the area percentage of $CD45^+$ cells in MVs instead of the number of $CD45^+$ cells was measured and calculated because many $CD45^+$ immune cells in MVs had extensions, which showed only a small part in the paraffin sections.

To quantify the FI of p-SMAD2, PROX1, p-VEGFR3, p-ERK1/2, p-Akt, and S1PR1 in LECs of MVs, confocal images of whole-mount immunostaining were taken under a 20x objective. The analysis was performed on 4~5 optical section images per sample, involving about 50~80 LECs. LECs were selected by thresholding $VEGFR3^+$ signals in ImageJ, the FI of these markers was then measured in selected LECs. To quantify the FI of CCR7 in $CD45^+$ immune cells in MVs, confocal images of frozen section immunostaining were obtained, and measurement was performed using the same method.

For the quantification of matrix deposition and cellularity in MV frozen sections (Supplemental Figure 8, B and C), area of HABP, Collagen I and pL were measured. Total cell number per pL section was counted based on the DAPI staining. % of HABP or Collagen I = area of HABP$^+$ or Collagen I$^+$ regions / area of pL x 100%. Cell number per 0.01 mm^2 of pL section (cell density) = Total cell number / area of pL x 10^4.

To quantify the percentages of HABP in aLs (Supplemental Figure 15G) and MHCII$^+$ cells in EC Zone 1 (Supplemental Figure 15H), the area of HABP$^+$ regions, MHCII$^+$ cells, aL, and EC Zone 1 were measured, respectively. % of HABP in aL = area of HABP$^+$ regions / area of aL x 100%. % of MHCII$^+$ cells in EC Zone 1 = area of MHCII$^+$ cells / area of EC Zone 1 x 100%.

Body weight monitoring

Mouse body weights were monitored daily from P1 to P60 before drug treatment. Body weight data were analyzed for the total cohort as well as stratified by sex (male and female).

Single cell RNA sequencing data analysis

A secondary analysis of the published scRNAseq data (38) was performed. The original data sets were obtained from WT and *Fbn1*$^{C1039G/+}$ MV samples at P30. Briefly, raw scRNA-seq data were accessed through GEO website (GSE261874). The data were analyzed using the Seurat package (version 5.1.0) in R. Raw gene expression matrices were first filtered to exclude low-quality cells and genes with low expression, based on thresholds for minimum gene count per cell and mitochondrial gene content. Data were then normalized using Seurat's LogNormalize method, and the top variable genes were

identified for downstream analysis. The WT and *Fbn1*$^{C1039G/+}$ MV samples were integrated using Canonical Correlation Analysis (CCA) to account for batch effects and Principal component analysis (PCA) was performed on the scaled expression matrix, and the top 40 principal components were used to construct a shared nearest neighbor (SNN) graph for cell clustering using the Louvain algorithm. Clusters were visualized using Uniform Manifold Approximation and Projection (UMAP). Differentially expressed genes (DEGs) for each cluster were identified using the Wilcoxon rank-sum test implemented in Seurat and known marker genes were used to annotate cell types. Major cell types such as VECs/LECs and macrophages/DCs were subsetted and re-clusters to discover potential subgroups. Gene ontology (GO) analysis results were reported based on the DEG data by using Metascape (https://metascape.org).

Flow cytometry analysis

For the MV samples, hearts from mice at P14 were perfused with cold PBS (4°C) after mouse anesthesia. Hearts were collected and MV leaflets were dissected in cold MACS® Tissue Storage Solution (Miltenyi Biotec, 130-100-008). The leaflets were processed for flow cytometry analysis as previously described (79). Briefly, MV leaflets were collected from 3 WT or *Fbn1* mutant mice and mixed in each repeat, total 3 repeats in each group. The tissue was chopped into small pieces, incubated and digested with 5 mg/mL collagenase (Roche, #11088866001) and 0.5 mg/mL DNase (Roche, #10104159001) in HBSS [Ca^+/Mn^+] for 30 min at 37 °C. After digestion, the tissue was gently mashed and filtered through 40 μm filter, lysed with RBC lysis buffer, stained with live/dead dye, incubated with FcBlock and antibody cocktails (Supplemental Table 6), fixed with 2% PFA, and used for flow cytometry analysis.

For blood, spleen, and lymph node samples, mice were euthanized at P16 after being treated with FTY720 or water (as control) daily from P1 to P15. Blood, spleen and lymph nodes (LNs, including inguinal, axillary, brachial, cervical and mesentery LNs) were collected and processed for flow cytometry analysis as previously described (58, 59, 79). Antibodies used are listed in Supplemental Table 6.

FTY720 and LPS treatment of HUVECs

Human Umbilical Vein Endothelial Cells (HUVECs) were isolated from a human donor (80) by Dr. M. Luisa Iruela-Arispe's laboratory and utilized between passages 7 and 10. The donor provided informed consent for the research. HUVECs were cultured in EGM-2 BulletKit (Lonza, #CC-3162, including EBM-2 basal medium and EGM-2 SingleQuots Supplements), containing 10% fetal bovine serum (FBS; Omega Scientific) with 1% penicillin/streptomycin (Gibco, #15-140-122).

For treatment of FTY720 (Sigma, SML0700-25MG) and lipopolysaccharide (LPS; Sigma, #L2630-25MG), HUVECs were seeded in 6-well plates at a density of 250,000 per well and cultured until 100% confluency was reached. Cells were pre-treated with FTY720 at 1 µM for 24 hours in EGM-2 BulletKit containing 10% FBS. Subsequently, the culture medium was replaced with EBM-2 basal medium supplemented with 1% FBS while maintaining FTY720 treatment to enhance responsiveness to LPS. After a 30-min equilibration period in the basal medium, cells were stimulated with LPS 1 µg/mL for 2 hours.

Cells were washed with cold DPBS with 2 mM Sodium Orthovanadate (Thermo Fisher Scientific, J60191.AE) and lysed using a Tris-based lysis buffer (20 mM Tris-HCl pH 7.5,

150 mM NaCl, 1 mM Na_2EDTA, 1 mM EGTA, 1% Triton X-100) supplemented with cOmplete EDTA-free Protease Inhibitors Cocktail (Sigma, 11873580001) for 10 min on ice. Lysates were clarified by centrifugation, and supernatants were collected for protein quantification using Pierce™ Detergent Compatible Bradford Assay Kit (Thermo Fisher Scientific, #23246), according to the manufacturer's instructions. As a control for the nonphosphorylated form of ZFP36, cell lysates were treated with Lambda Protein Phosphatase (APExBIO, K1102) prior to Western blotting.

Western blotting

For Western blot analysis, equal amounts of protein lysates were resolved on 8% SDS-polyacrylamide gels under reducing conditions and transferred onto nitrocellulose membranes (Bio-Rad Laboratories). Total proteins were confirmed by Ponceau S staining solution (Thermo Fisher Scientific, #A40000278). Membranes were blocked for 1 h at room temperature with 2% non-fat dry milk in 1x TBS buffer containing 0.1% Tween-20 (TBST) and incubated overnight at 4C with a rabbit monoclonal anti-Tristetraprolin (ZFP36) antibody (Cell Signaling Technology, #71632, 1:500). After washing with TBST, membranes were incubated with horseradish peroxidase-conjugated anti-rabbit (Fisher Scientific, 45-000-682, 1:10000). Protein bands were visualized using enhanced chemiluminescence (SuperSignal West Pico Chemiluminescent Substrate, Fisher Scientific, #PI34580), under ChemiDoc MP Imaging system (Bio-Rad). Band intensities were quantified by densitometric analysis using Image Lab software (Bio-Rad).

Echocardiography

Non-invasive transthoracic echocardiography was used to evaluate cardiac function in

Fbn1 mutant mice and their WT littermates at P60 with water or FTY720 treatment. Animals were sedated by the inhalation of 1.5% isoflurane in oxygen. After chest hair was removed with Nair, echocardiography was performed with a Visualsonics Vevo 3100 imaging system equipped with a high-frequency transducer MX550S (FUJIFILM VisualSonics, Toronto, Canada), as previously described (81). High-resolution B-mode and M-mode images were acquired from the parasternal short-axis view at the level of the papillary muscles. Cardiac parameters, including left ventricular ejection fraction (EF) and fractional shortening (FS), were calculated using the LV Trace tool within the Cardiac Package of Vevo LAB analysis software (version 5.6.1; FUJIFILM VisualSonics).

Supplemental Figures

Supplemental Figure 1

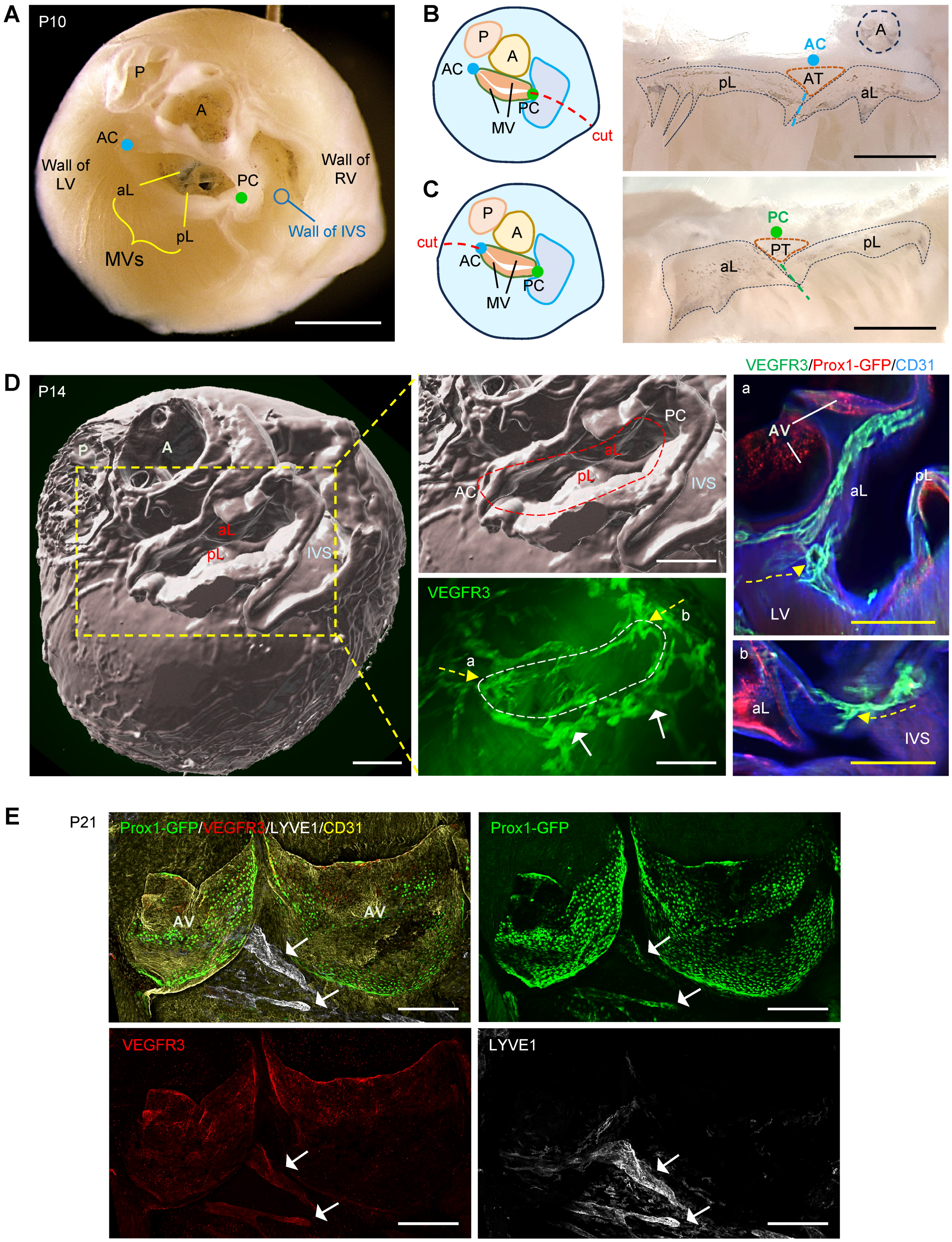

Supplemental Figure 1. Lymphatic development in mouse mitral valves.
(A) A view from the perspective of the cardiac top in a WT mouse shows distinct structures. MV: mitral valves, aL/pL: anterior/posterior leaflet, AC/PC: anterior/posterior commissure, P: pulmonary artery, A: aorta, LV/RV: left/right ventricle, IVS: interventricular septum. Scale bars = 1mm. **(B, C)** Two cutting methods for the upper half of the heart can reveal the anterior **(B)** or posterior **(C)** triangle. aL/pL: anterior/posterior leaflet, AT/PT: anterior/posterior triangle, AC/PC: anterior/posterior commissure, A: aorta. Scale bars = 1mm. **(D)** 3-D reconstruction of the upper half of a WT heart with *Prox1-eGFP* reporter (red), stained with VEGFR3 (green) and CD31 (blue) shows the cardiac structure (black-and-white 3-D heart) and the distribution of lymphatic vessels (VEGFR3, green) in or near the mitral valves. Prox1-eGFP (red channel) is detected not only in LECs but also in VECs on cardiac valve leaflets. aL/pL: anterior/posterior leaflet, AC/PC: anterior/posterior commissure, LV: left ventricle, IVS: interventricular septum, AV: aortic valves. Circles indicate the proximal edge of the MVs. Yellow dotted arrows show the orientation and location of lymphatic vessel penetration. White arrows indicate lymphatics derived from the left ventricular dorsal wall and the right posterior side of the interventricular septum. White/yellow Scale bars = 400/300 µm. **(E)** Representative confocal images of the aortic valves (AVs) of a WT mouse at P21. Arrows indicate lymphatic vessels (LYVE1$^+$VEGFR3$^+$Prox1-eGFP$^+$) outside the AV region. Note that Prox1-eGFP is expressed in both VECs and LECs. Scale bars = 200 µm.

Supplemental Figure 2

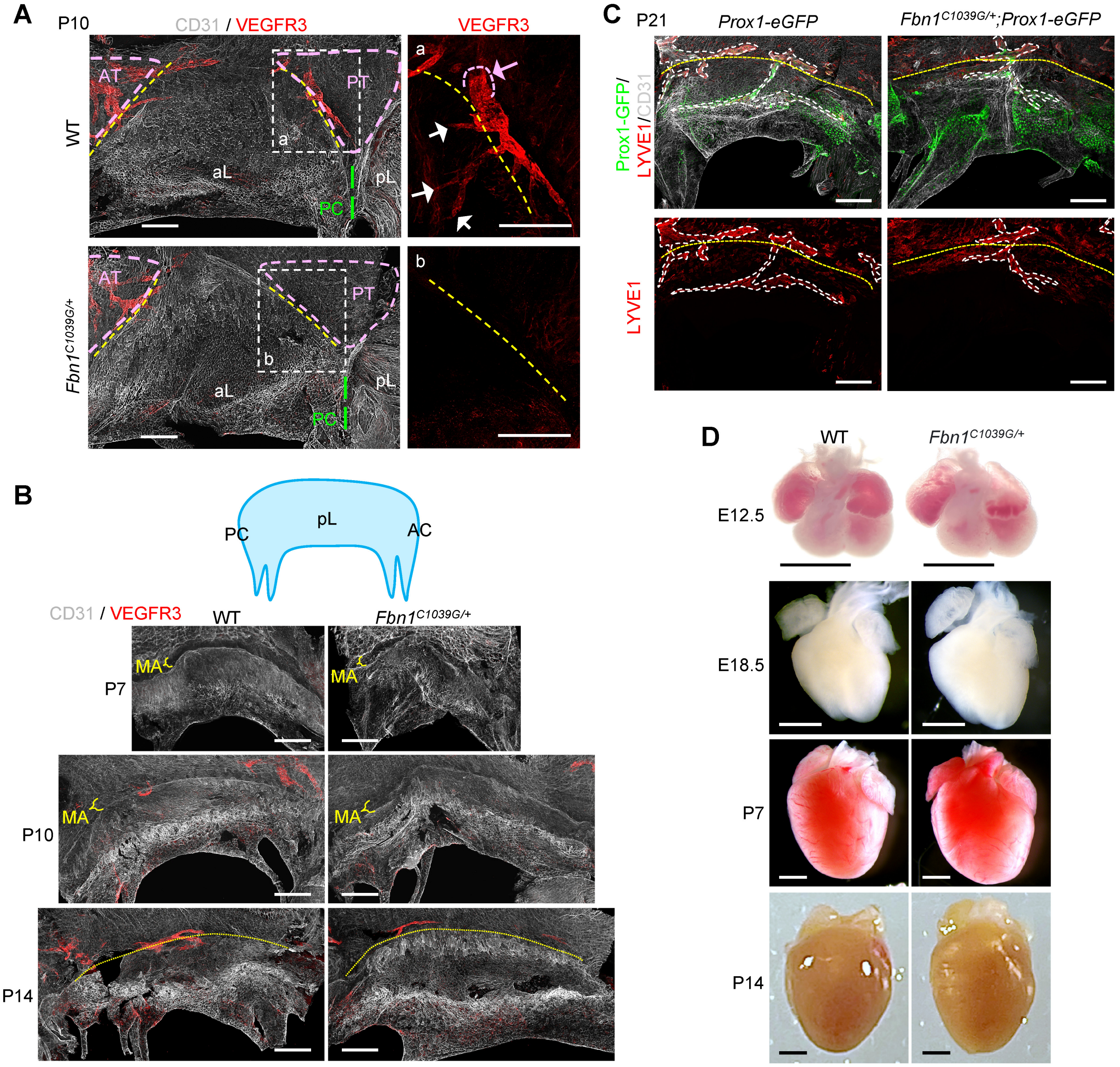

Supplemental Figure 2. Lymphatic growth is inhibited in the mitral valves of *Fbn1* mutant mice.
(A) Representative confocal images show lymphatic vessels in the posterior triangle (PT) at P10. AT/PT circled by pink dotted lines: anterior/posterior triangle, aL/pL: anterior/posterior leaflet, PC: posterior commissure. White arrows indicate lymphatic sprouts. Yellow lines indicate the proximal edge of the leaflets. The pink arrow shows the site where the lymphatic vessel trunk passes out from below in the posterior triangle. Scale bars = 200 μm. **(B)** Development of lymphatics in posterior leaflets (pL) at early ages. AC/PC: anterior/posterior commissure, MA: mitral annulus. Yellow lines indicate the proximal edge of the leaflets. Scale bars = 200 μm. **(C)** Representative confocal images show lymphatic vessels (Prox1-eGFP^{+}LYVE1^{+}, outlined by white dotted lines) in the posterior leaflets of mice with the *Prox1-eGFP* reporter at P21. Yellowed dotted lines indicate the proximal edge of leaflets. Scale bars = 200 μm. **(D)** WT and *Fbn1* mutant hearts at different stages. Scale bars = 1 mm.

Supplemental Figure 3

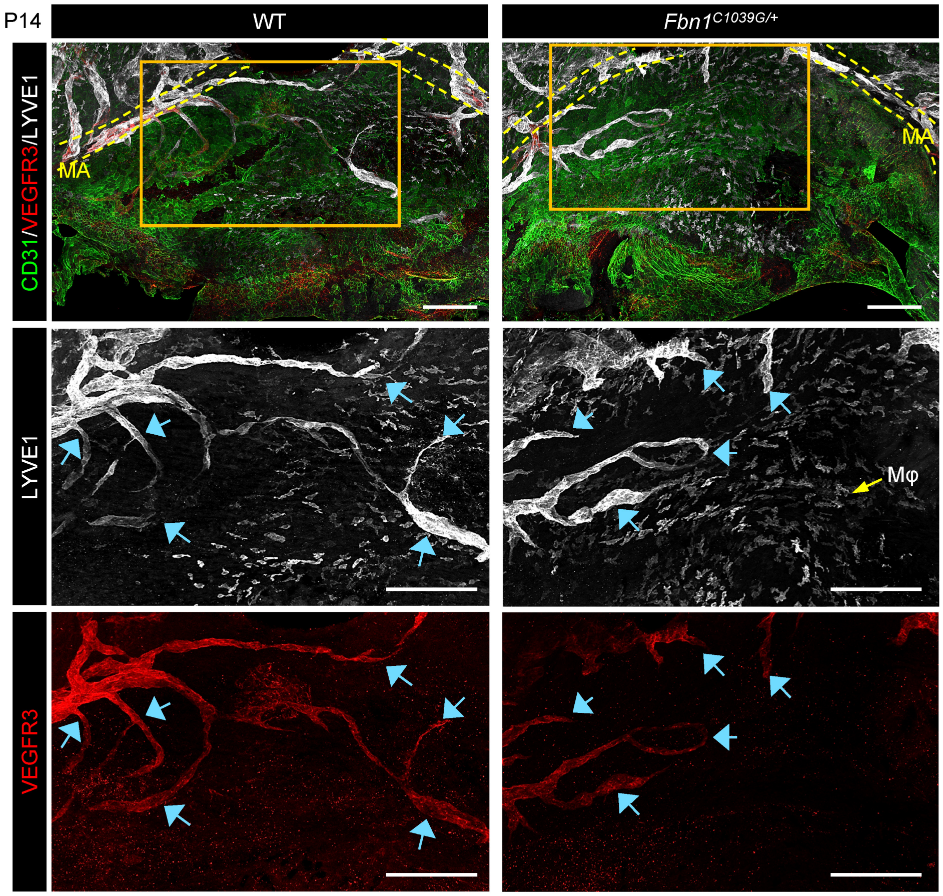

Supplemental Figure 3. Lymphatic growth is suppressed in *Fbn1* mutant mitral valves.
Representative confocal images of whole-mount MVs at P14, labeled with the pan-endothelial marker CD31 (green) and the LEC markers VEGFR3 (red) and LYVE1 (white). Blue arrows indicate lymphatic branches. LYVE1 stains lymphatics and labels macrophages (Mφ). MA: mitral anulus. Scale bars = 200 μm.

Supplemental Figure 4

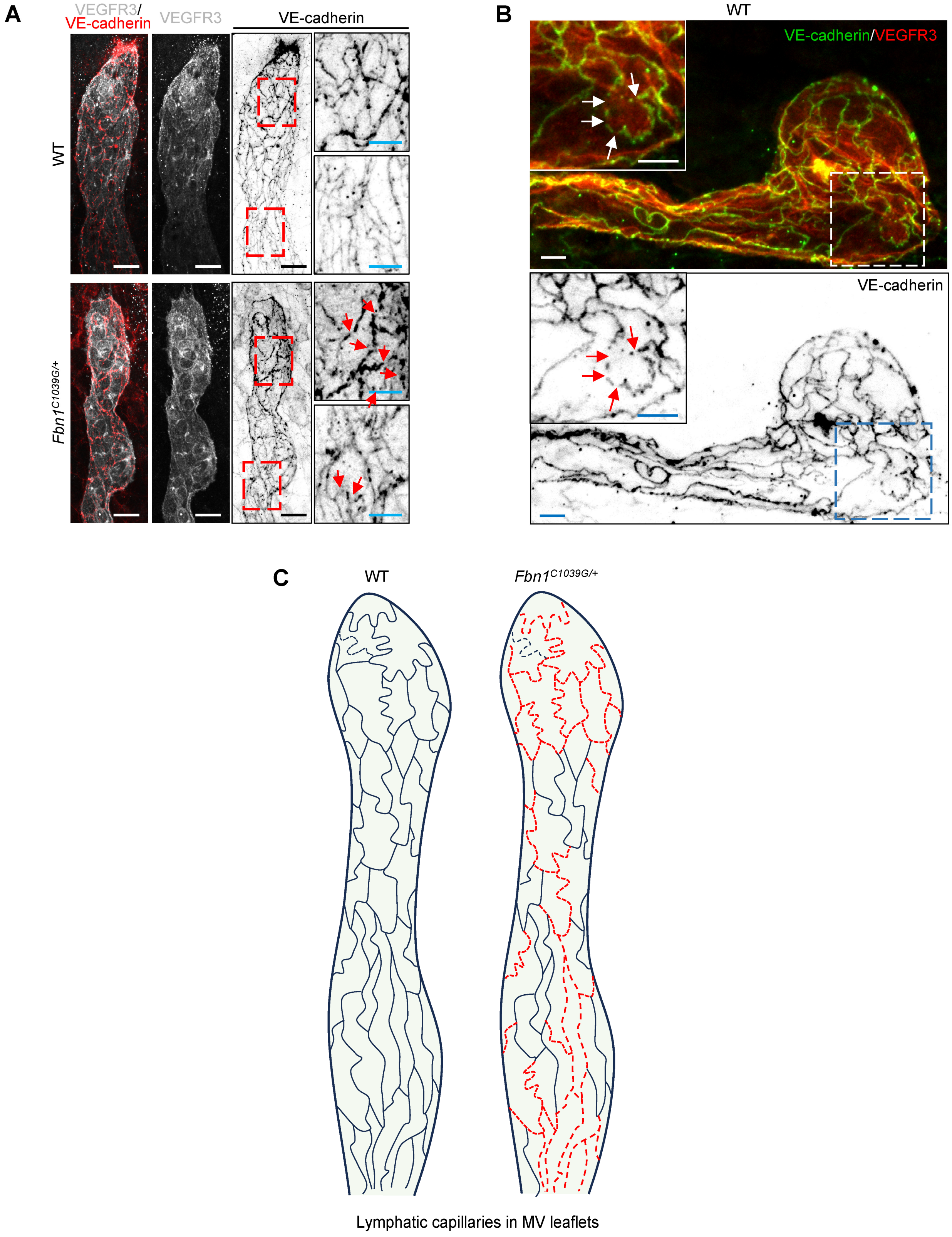

Supplemental Figure 4. Disrupted cell-cell junctions in lymphatic capillaries of *Fbn1* mutant mitral valves.
(A) Representative confocal images of whole-mount MVs at 6 weeks of age show the cell-cell junctions of lymphatic capillaries. Arrows indicate disrupted junctions in the mutant, compared with the continuous junctions in WT. White and black scale bars = 20 μm; blue scale bars = 10 μm. **(B)** Representative confocal images show discontinuous, button-like LEC junctions (arrows) at the tip of initial lymphatic capillary in the WT anterior leaflet. Most LEC junctions in the WT lymphatic capillaries are continuous zipper junctions. Scale bars = 10 μm. **(C)** Schematic diagram shows cell-cell junctions in lymphatic capillaries of MVs. Red dotted lines indicate disrupted lymphatic cell junctions in *Fbn1* mutant MVs.

Supplemental Figure 5

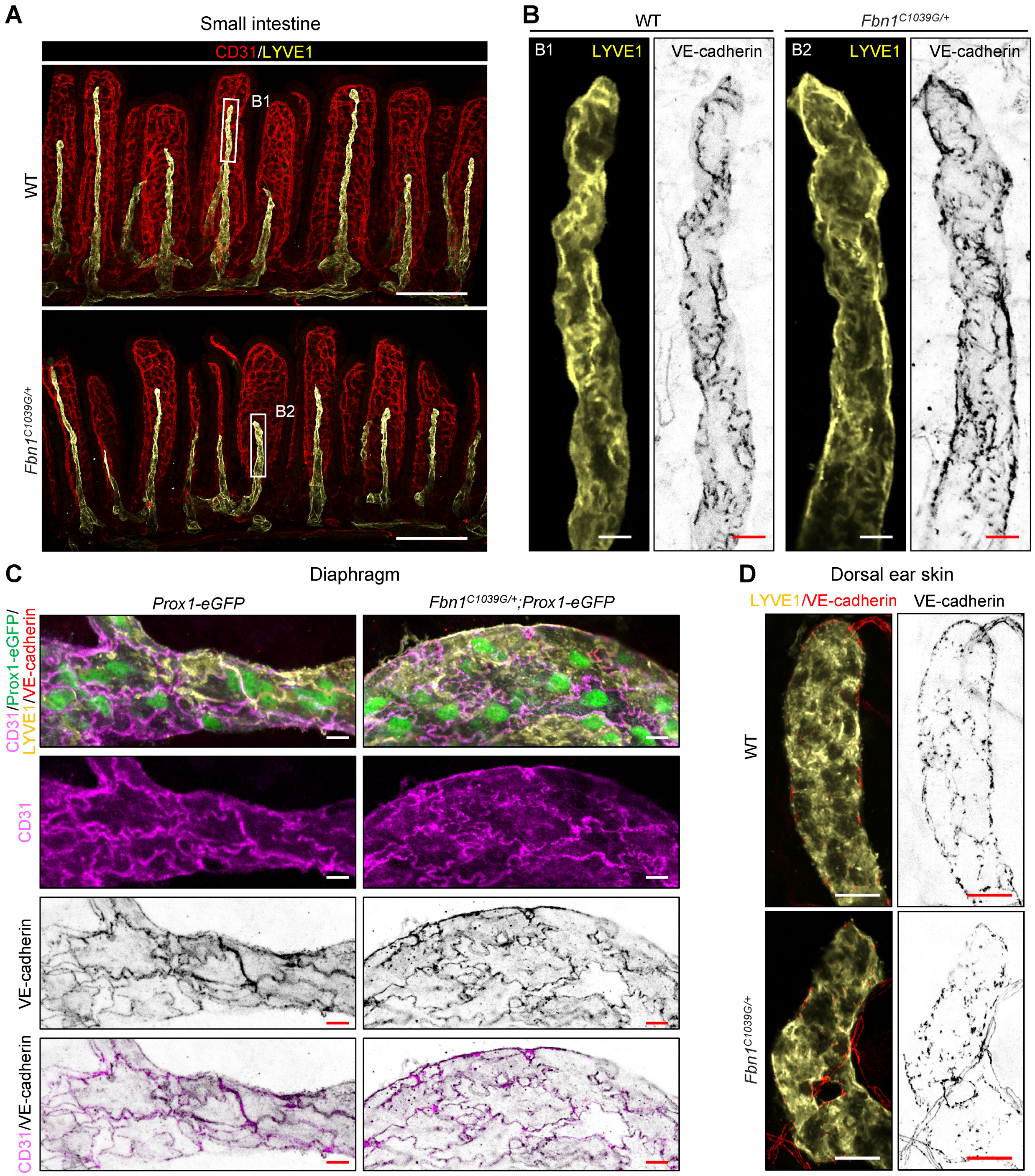

Supplemental Figure 5. LEC junctions in lymphatic capillaries in different organs.
Representative confocal images of immunostaining in whole-mount tissues show LEC button-like junctions (labeled with VE-cadherin) in lymphatic capillaries (labeled with LYVE1) of lacteal **(A, B)**, diaphragm **(C)**, and dorsal ear skin **(D)** in both WT and *Fbn1* mutant mice at 6 weeks of age. Scale bars = 200 µm in **A**, 10 µm in **B** and **C**, 20 µm in **D**.

Supplemental Figure 6

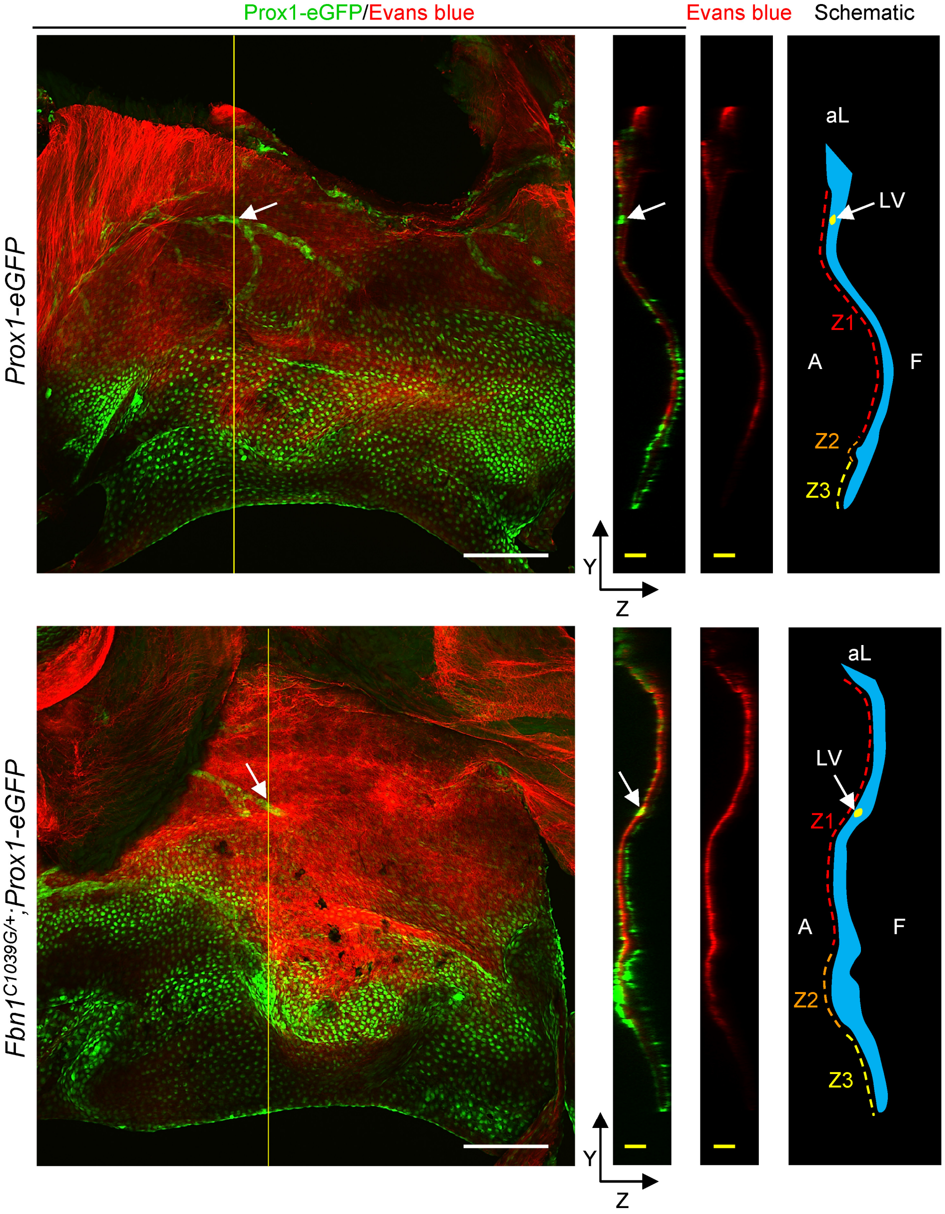

Supplemental Figure 6. Evans blue accumulates in *Fbn1* mutant mitral valves.
Resliced 2-D images (orthogonal view along the YZ axis) from the 3-D image stacks (shown in Figure 2D) of whole-mount MV anterior leaflets (aL) along the yellow lines crossing the lymphatic vessels show the distribution of retained Evans blue in the interstitium beneath different endothelial cell zones (Z1~3) at the atrial side of the MV leaflet. A/F: atrial/fibrosa side. Arrows indicate the lymphatic vessels (LV). White/yellow scale bars = 200/50 μm. In the schematic diagram, the blue region represents the leaflet interstitium, while the green dots indicate cross sections of lymphatic vessels.

Supplemental Figure 7

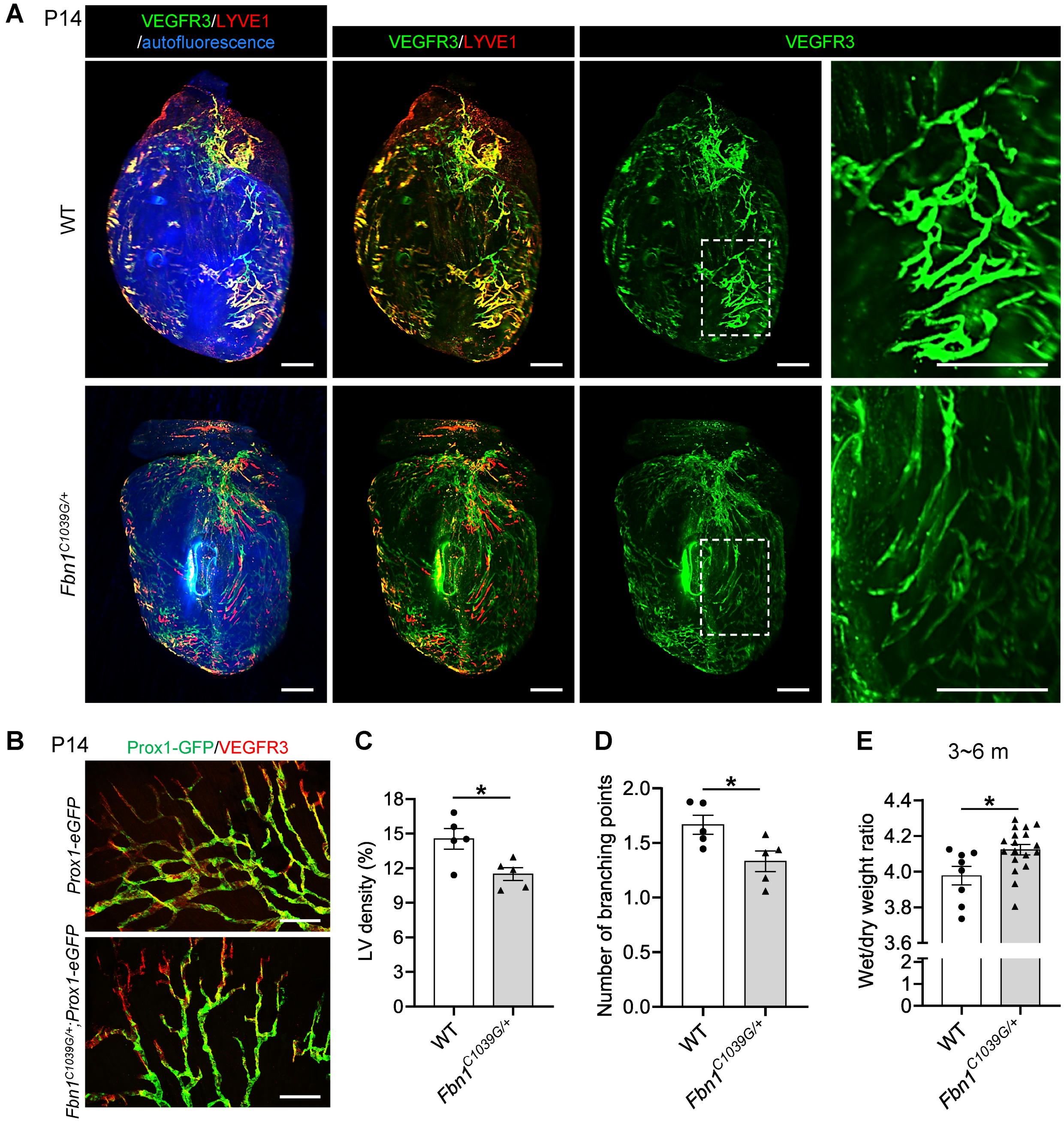

Supplemental Figure 7. Hypogenesis of lymphatics in *Fbn1* mutant heart.
(A) Representative light sheet microscope images of whole-mount hearts at P14, stained with VEGFR3 and LYVE1, show lymphatics in the sub-epicardium of ventricles. Scale bars = 150 μm. **(B)** Representative confocal images of whole-mount left ventricles at P14 show cardiac lymphatic vasculatures stained with VEGFR3 (red) in mice with a *Prox1-eGFP* reporter (green). Scale bars = 200 μm. **(C, D)** Quantification of lymphatic vessel (LV) density (% of left ventricular surface area) **(C)** and the number of lymphatic branching points per 0.01 mm^2 of lymphatic vessel area **(D)** was performed based on confocal images of left ventricles as shown in **B**. Data are Mean ± SEM, Student's unpaired *t*-test, each symbol represents one mouse, N=5, 3 males and 2 females per group, **P*<0.05. **(E)** Quantification of the wet/dry weight ratio in hearts collected at ages 3~6 months. Data are Mean ± SEM, Student's unpaired *t*-test, each symbol represents one mouse, N = 8~18, male: female = 3:5 and 7:11 in WT and *Fbn1* mutant groups, respectively. **P*<0.05.

Supplemental Figure 8

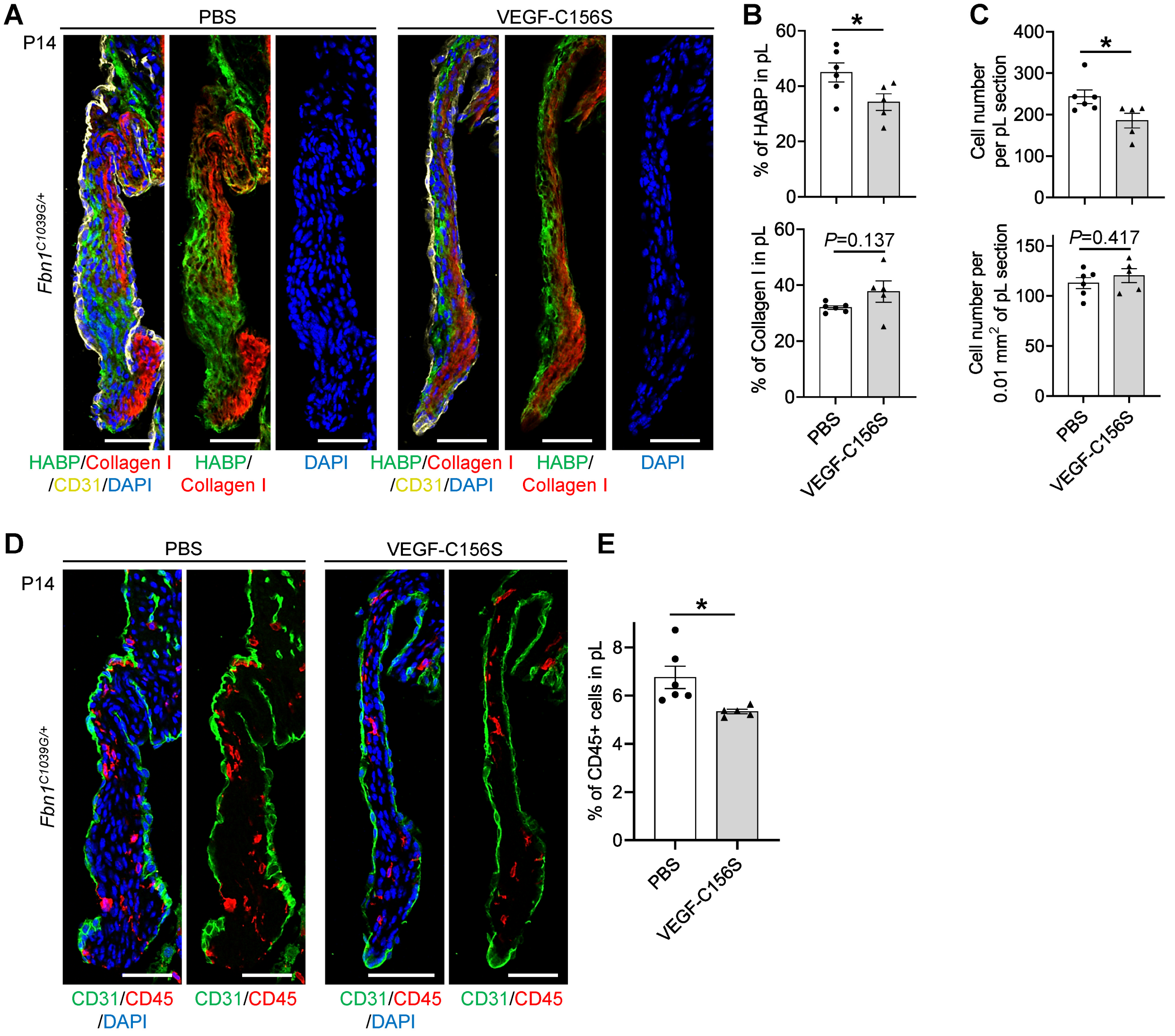

Supplemental Figure 8. VEGF-C156S ameliorates pathological matrix deposition and mitigates inflammatory infiltration in *Fbn1* mutant MVs.
(A) Representative confocal images of immunostaining on frozen sections (10 μm) of MVs from *Fbn1* mutant mice at P14, after PBS (vehicle control) or VEGF-C156S treatment, show the distribution of hyaluronic acid-binding protein (HABP) and Collagen I in posterior leaflets (pL). Scale bars = 50 μm. Quantification of the percentages of HABP and Collagen I in pL **(B)**, cell number per pL section, and cell number per 0.01 mm^2 of pL section (cell density in pL) **(C)** was performed based on the image data in **A**. Representative confocal images of CD45 immunostaining **(D)** and quantification of the percentage of $CD45^+$ immune cells in pL **(E)** for the same samples mentioned above demonstrate a reduction in $CD45^+$ cells in *Fbn1* mutant MV after VEGF-C156S treatment. Data in **B**, **C**, and **E** are Mean ± SEM, Student's unpaired *t*-test, each symbol represents one mouse, N=5~6, 2~3 males and 3 females per group, *$P<0.05$.

Supplemental Figure 9

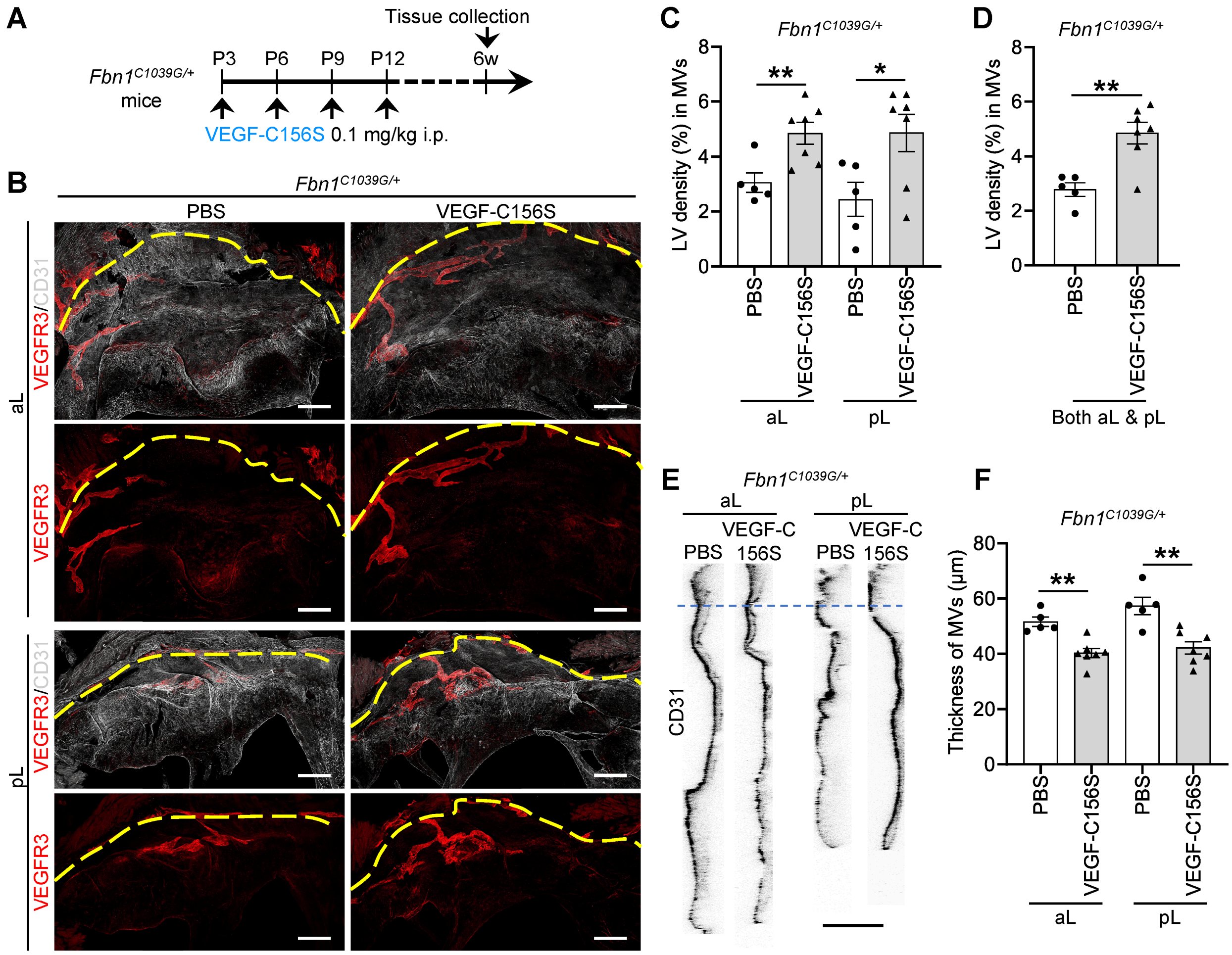

Supplemental Figure 9. Therapeutic benefits of early-life VEGF-C156S intervention persist throughout development and into adulthood.

(A) Timeline of VEGF-C156S treatment and tissue collection for B-F. **(B)** Representative confocal images of whole-mount anterior (aL) and posterior (pL) leaflets from *Fbn1* mutant mice at 6 weeks of age under PBS (vehicle control) or VEGF-C156S treatment. Yellow dotted lines indicate the proximal edge of leaflets. Scale bars = 200 μm. Quantification of lymphatic vessel (LV) density in aL **(C)**, or pL **(C)** or both leaflets (mean values from aL and pL) **(D)** was performed based on the image data shown in **B**. **(E)** Resliced 2-D images from the 3-D image stacks of whole-mount MV leaflets along the midline of each leaflet. aL/pL: anterior/posterior leaflet. Scale bars = 200 μm. **(F)** Quantification of MV leaflet thickness based on the data shown in **E**. Data in **C**, **D** and **F** are Mean ± SEM, Student's unpaired *t*-test, each symbol represents one mouse. N = 5~7, male: female = 4:1 and 4:3 in PBS and VEGF-C treated groups, respectively. **P* < 0.05, ***P* < 0.01, *n.s.*, not significant.

Supplemental Figure 10

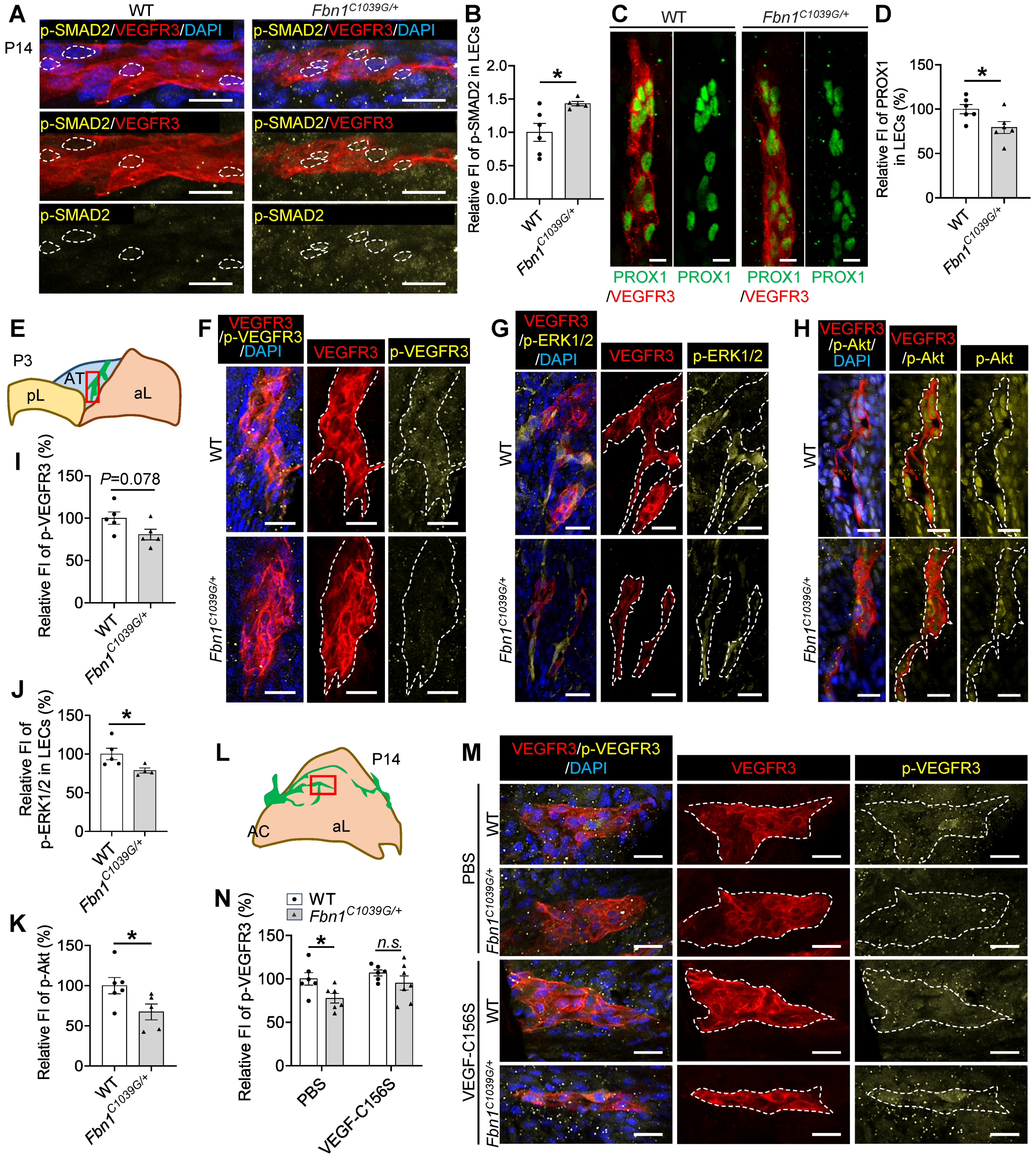

Supplemental Figure 10. Mechanisms of inhibited lymphangiogenesis in *Fbn1* mutant mitral valves.
(A-D) Representative confocal images **(A, C)** and quantification of fluorescent intensity (FI) **(B, D)** show expression of p-SMAD2 **(A, B)** and PROX1 **(C, D)** in lymphatics of whole-mount anterior MV leaflets at P14. Scale bars = 20 μm and 10 μm in **A** and **C**, respectively. Data in **B** and **D** are Mean ± SEM, Student's unpaired *t*-test, each symbol represents one mouse. N = 5~6, male: female = 2~3:3 per group in **B** and 3:3 per group in **D**. $^*P < 0.05$.

(E-K) Representative confocal images **(F-H)** and quantification of fluorescent intensity (FI) **(I-K)** show expression of p-VEGFR3 **(F, I)**, p-ERK1/2 **(G, J)**, and p-Akt **(H, K)** in lymphatics within the anterior triangle (AT) at P3 **(E)** (before VEGF-C156S treatment). Scale bars = 25 μm in **F** and **G**, 20 μm in **H**. Data in **I-K** are Mean ± SEM, Student's unpaired *t*-test,

each symbol represents one mouse. N = 5 (male: female = 3:2) in **I**; N=4~5 (male: female = 2:2~3) in **J**; N=5~6 (male: female = 3~4:2) in **K**. *$P < 0.05$.

(L-N) At P14, after treatment with PBS (vehicle control) or VEGF-C156S, LVs in the anterior leaflets (aL) were analyzed **(L)** for p-VEGFR3 expression by whole-mount immunostaining **(M)** and for the fluorescent intensity (FI) of p-VEGFR3 in LECs **(N)**. Scale bars = 25 μm in **M**. Data in **N** are Mean ± SEM, Student's unpaired *t*-test, each symbol represents one mouse. N = 6~7, male: female = 2:4, 3:3, 3:3, and 4:3 in the groups of WT-PBS, *Fbn1*-PBS, WT-VEGF-C156S, *Fbn1*-VEGF-C156S, respectively. *$P < 0.05$. *n.s.*, not significant.

Supplemental Figure 11

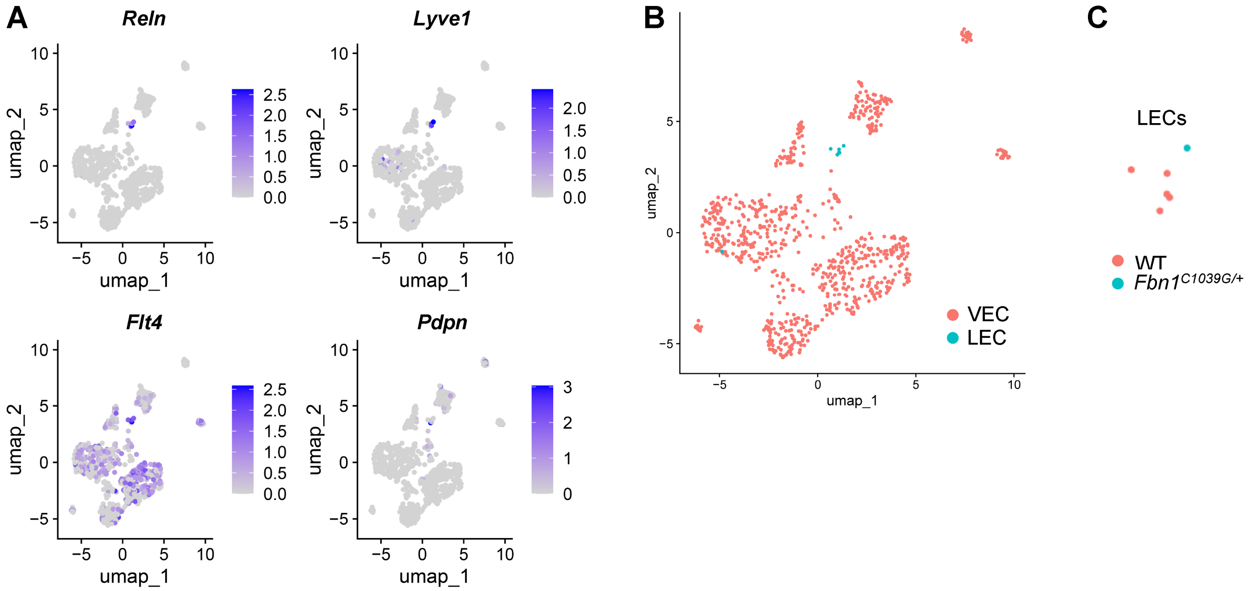

Supplemental Figure 11. LECs are identified within the EC cluster of MVs.
(A) UMAPs show expression of 4 LEC markers in the EC cluster. **(B)** LECs are identified within the EC cluster shown in Figure 5A based on the LEC markers in A. Other cells in the EC cluster are VECs. **(C)** LEC cluster from WT and *Fbn1* mutant MVs.

Supplemental Figure 12

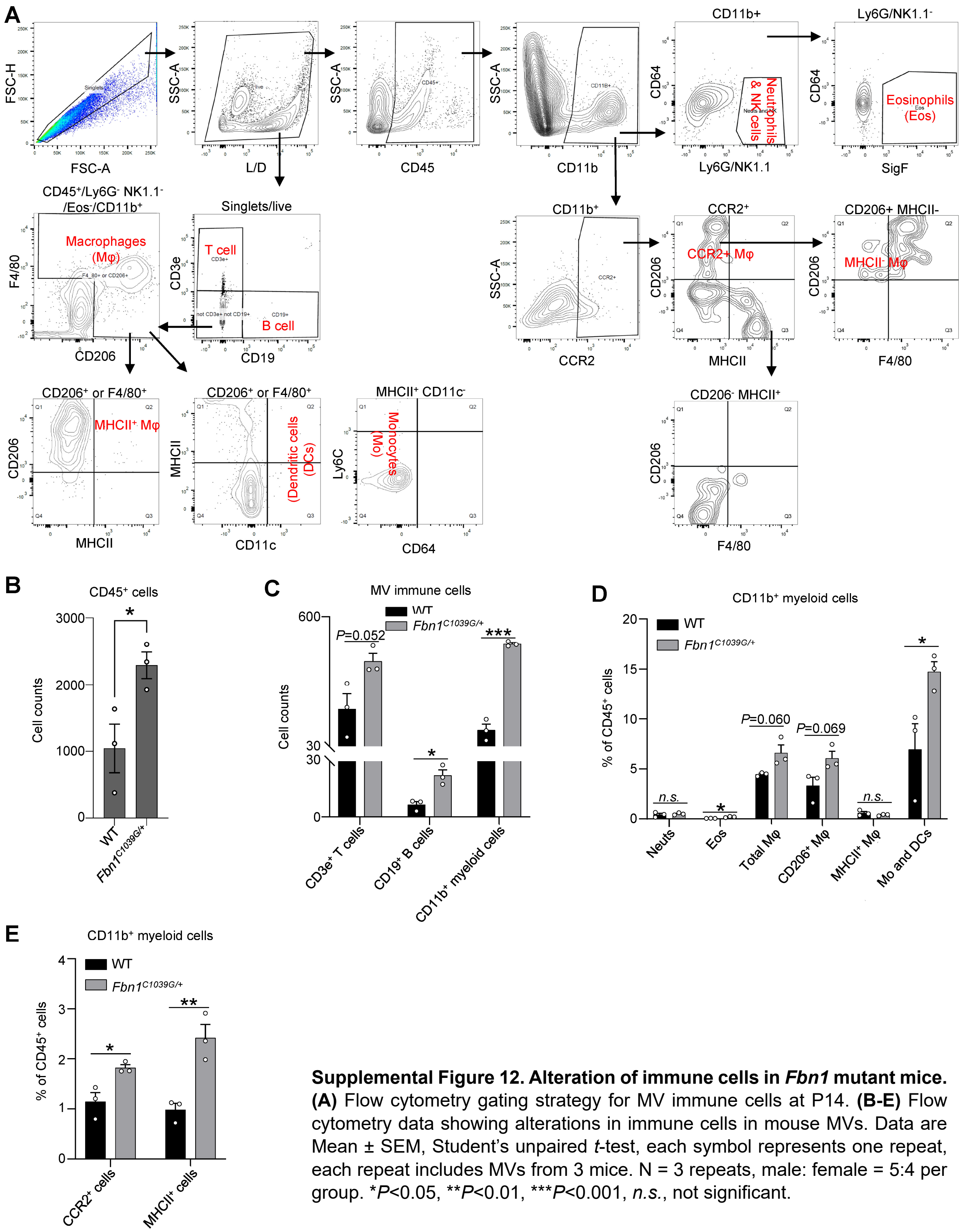

Supplemental Figure 12. Alteration of immune cells in *Fbn1* mutant mice. (A) Flow cytometry gating strategy for MV immune cells at P14. **(B-E)** Flow cytometry data showing alterations in immune cells in mouse MVs. Data are Mean ± SEM, Student's unpaired *t*-test, each symbol represents one repeat, each repeat includes MVs from 3 mice. N = 3 repeats, male: female = 5:4 per group. *P<0.05, **P<0.01, ***P<0.001, *n.s.*, not significant.

Supplemental Figure 13

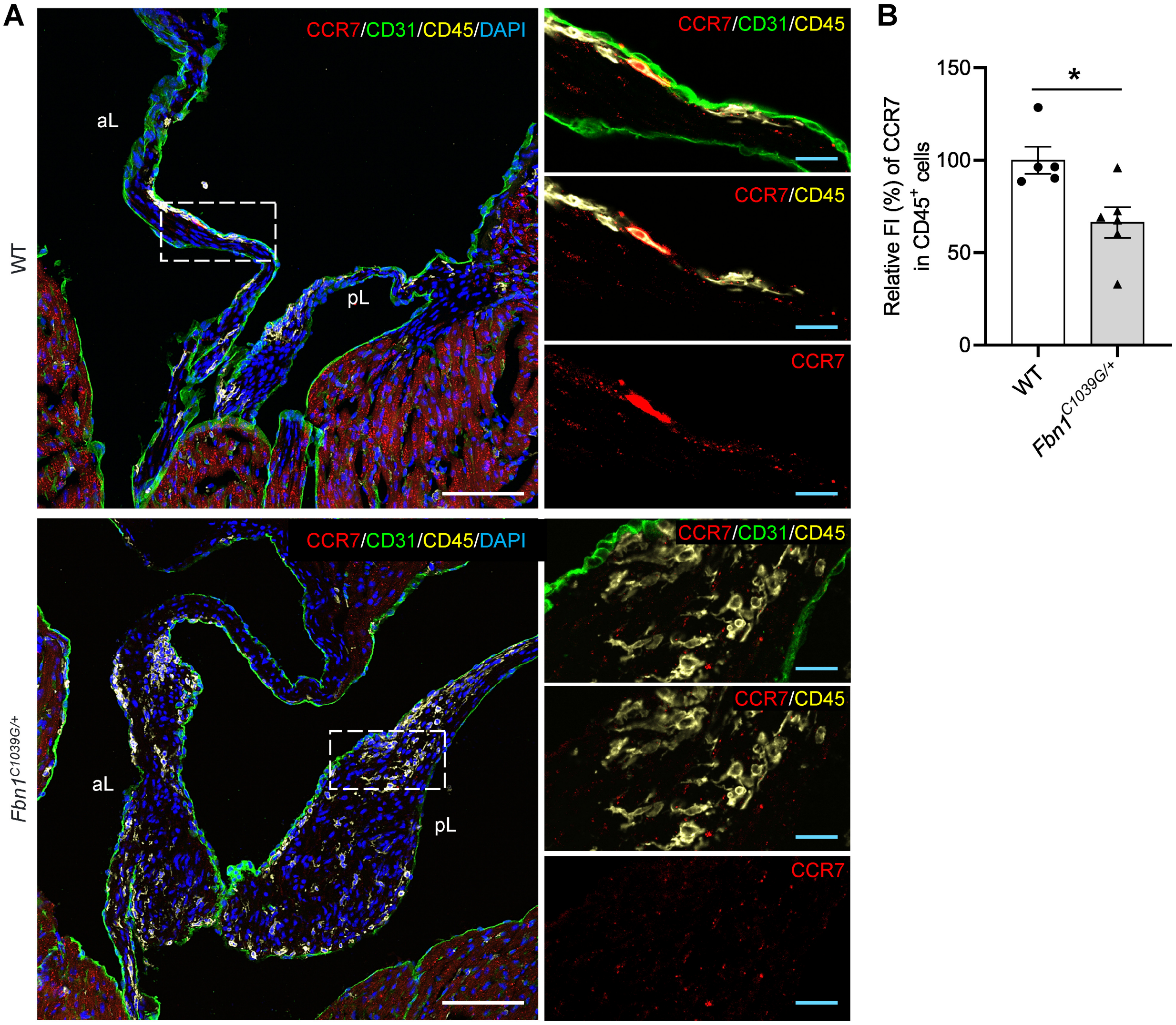

Supplemental Figure 13. CCR7 is downregulated in CD45^{+} immune cells within *Fbn1* mutant mitral valves.
(A) Representative confocal images of immunostaining on frozen MV sections (10 μm) from mice at P30. aL/pL: anterior/posterior leaflet. White/blue cale bars = 100/20 μm. **(B)** Quantification of CCR7 fluorescent intensity (FI) in CD45^{+} cells. Data are Mean ± SEM, Student's unpaired t-test, each symbol represents one mouse. N = 5~6, male: female = 3:2~3 per group. *P < 0.05.

Supplemental Figure 14

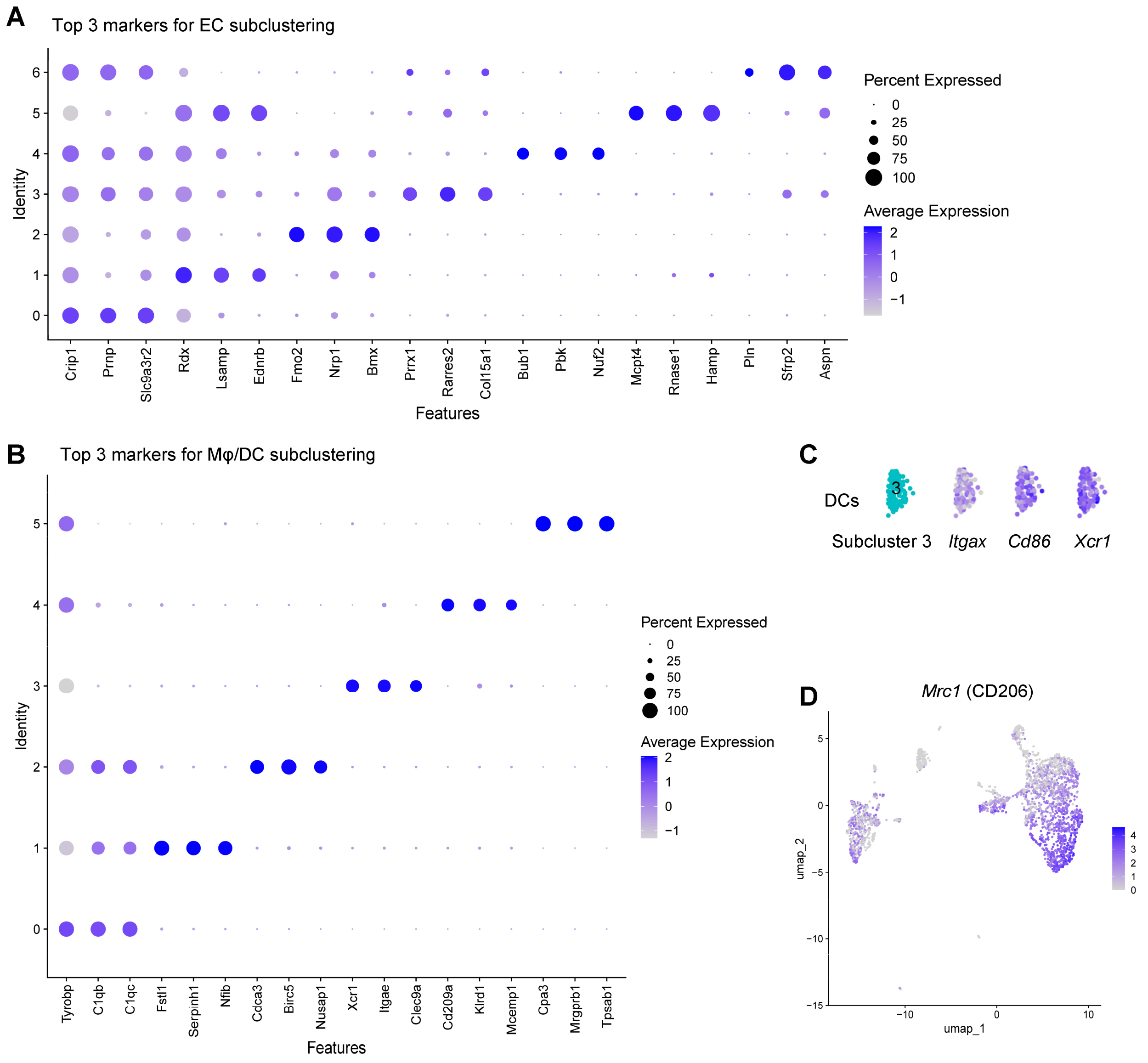

Supplemental Figure 14. Subclustering of ECs and macrophages/dendritic cells.
(A, B) Dot plots show the top 3 markers for subclusters of the endothelial cell (EC) cluster **(A)** and the macrophage/dendritic cell (Mφ/DC) cluster **(B)**. **(C)** UMAP shows identification of dendritic cells (DCs), the subcluster 3 of Mφ/DC, using DC markers. **(D)** UMAP shows gene expression of *Mrc1* (CD206) in Mφ/DC subclusters.

Supplemental Figure 15

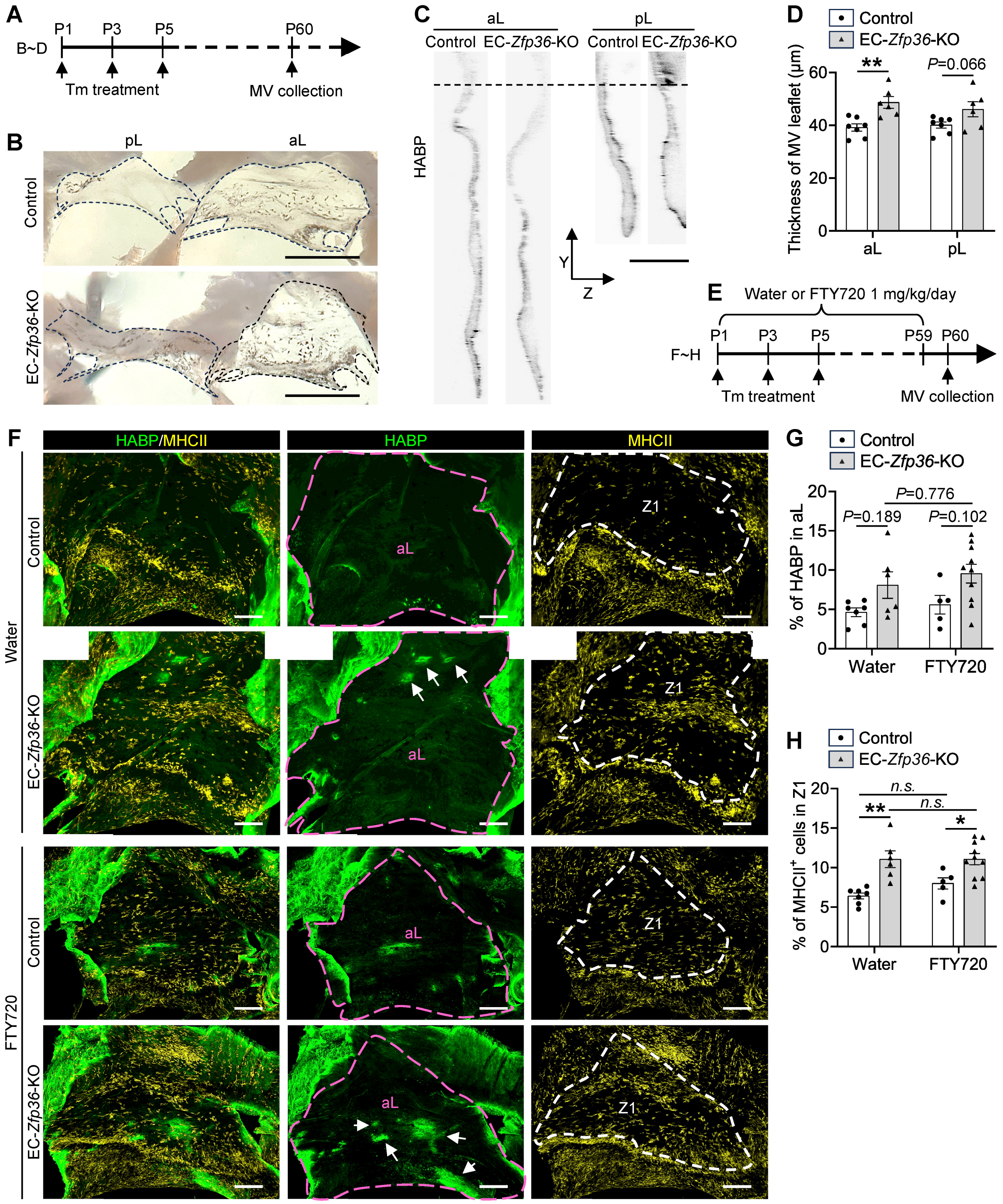

Supplemental Figure 15. Endothelial cell-specific deletion of *Zfp36* induces inflammation in the mitral valves. (A) Timeline of tamoxifen (Tm) treatment and tissue collection for B~D. **(B)** Representative images of flat-mount mitral valves from the atrial perspective under a stereo microscope. Broken lines outline the anterior (aL) and posterior (pL) leaflets. The darker background surrounding the mitral valves is the cardiac muscle. Black dots or areas on the MVs are melanocytes. Scale bars = 1 mm. **(C)** Representative resliced 2-D images (orthogonal view for YZ axis) from the

3-D image stacks of whole-mount MV leaflets (as shown in **F**) stained with hyaluronic acid binding protein (HABP), show MV morphology and thickness. aL/pL: anterior/posterior leaflet. Scale bars = 200 μm. **(D)** Quantification of MV thickness. aL/pL: anterior/posterior leaflet. Data are Mean ± SEM, Student's unpaired *t*-test, each symbol represents one mouse, N = 6~7, 2 males and 4~5 females. **P<0.01. **(E)** Timeline of drug treatments and tissue collection for **F~H**. **(F)** Whole-mount immunostaining of anterior MV leaflets (aL) shows local accumulation of HABP (arrows) in MVs and an increased number of $MHCII^+$ immune cells in the interstitium beneath EC Zone 1 (Z1) in EC-*Zfp36*-KO mice treated with water or FTY720. The boundaries of aL and EC zone 1 are indicated by pink and white dotted lines, respectively. Scale bars = 200 μm. **(G and H)** Quantification of the percentages of HABP (%= $HABP^+$ region area/aL area x 100%) **(G)** and $MHCII^+$ cells (%=$MHCII^+$ cell area/EC-Z1 area x 100%) **(H)** in the anterior leaflet and EC Zone 1, respectively. Data are Mean ± SEM, Ordinary one-way ANOVA test, each symbol represents one mouse, N = 5~10, male: female = 2:5, 2:4, 2:3, and 5:5 in the control-water, mutant-water, control-FTY720, and mutant-FTY720 groups, respectively. *P<0.05, **P<0.01, *n.s.*, not significant.

Supplemental Figure 16

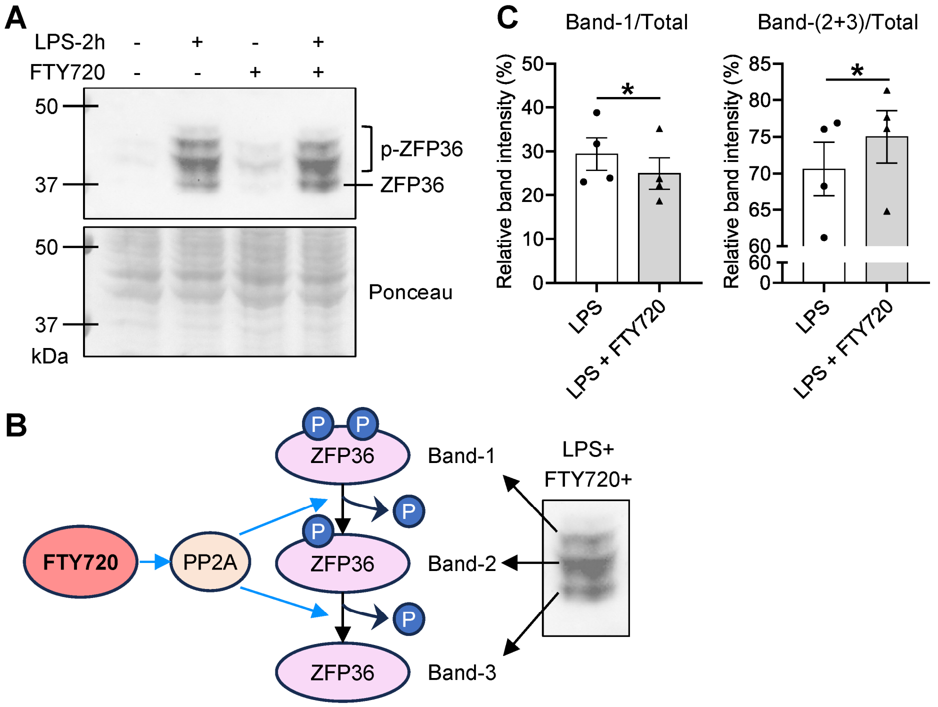

Supplemental Figure 16. FTY720 induces dephosphorylation of ZFP36.
HUVECs were pre-treated with 1 μM FTY270 for 24 h, followed by co-treatment with 1 μg/mL LPS and 1 μM FTY720 for an additional 2 h. **(A)** Representative blots show ZFP36 expression with/without LPS/FTY720 treatment, including the unphosphorylated form (ZFP36, bottom band) and the phosphorylated form (p-ZFP36, upper bands). Lambda Protein Phosphatase-treated control lysates were used as a reference to identify the unphosphorylated form of ZFP36 (See the file for Uncut blot images). A Ponceau S-stained membrane demonstrates similar protein loading across all lanes. **(B)** Schematic diagram of FTY720-induced dephosphorylation of ZFP36. Bands 1~3 indicate various phosphorylation states of ZFP36. **(C)** Quantification of relative band intensities (%). Bands 1 and 2 represent phosphorylated forms, while Band 3 represents the unphosphorylated form of ZFP36, as indicated in **B**. “Total” refers to the combined intensity of all three bands. Data are Mean ± SEM, Student’s paired *t*-test, each symbol represents one experiment, N = 4, *P<0.05.

Supplemental Figure 17

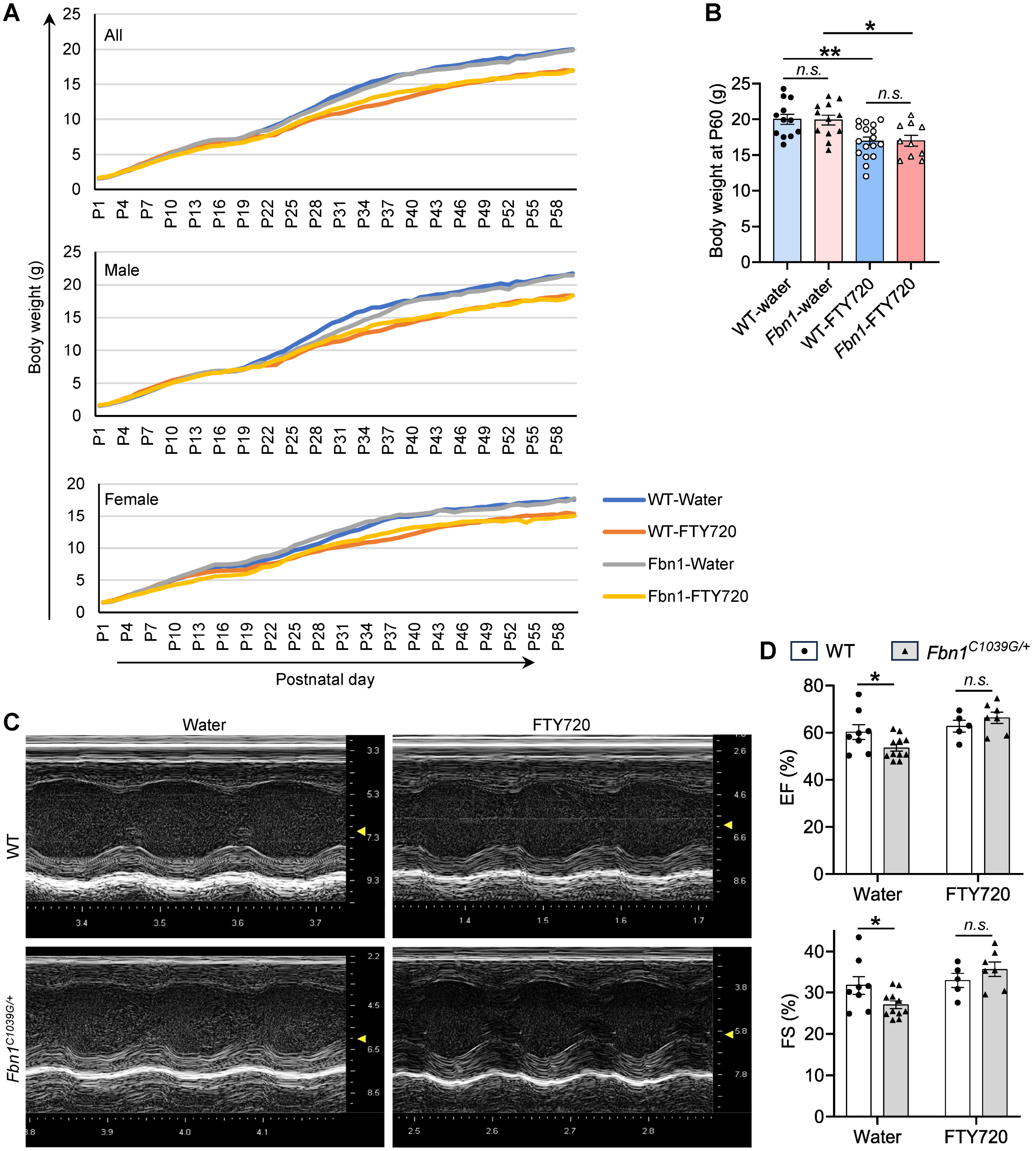

Supplemental Figure 17. Effect of FTY720 on mouse body weight and cardiac function.
(A, B) Body weight of mice treated with water or FTY720 was monitored daily from P1 to P60. **(A)** Body weight curves from P1 to P60 in all mice, male and female. Male: female = 7:5, 7:5, 9:8, and 6:4 in the WT-water, *Fbn1*-water, WT-FTY720, and *Fbn1*-FTY720 groups, respectively. **(B)** Comparation of mouse body weight at P60 among 4 groups. Data are Mean ± SEM, Ordinary one-way ANOVA test, each symbol represents one mouse, N = 10~17, **P* < 0.05, ***P* < 0.01, *n.s.*, not significant. **(C)** Representative M-mode echocardiograms obtained from the parasternal short-axis view of mouse hearts at P60 after water or FTY720 treatment. **(D)** Quantification of ejection fraction (EF) and fractional shortening (FS). Data are Mean ± SEM, Student's unpaired *t*-test, each symbol represents one mouse, N = 5~11, male: female ratios were 4:4, 7:4, 3:2, and 3:4 in the WT-water, *Fbn1*-water, WT-FTY720, and *Fbn1*-FTY720 groups, respectively. **P* < 0.05, *n.s.*, not significant.

Supplemental Figure 18

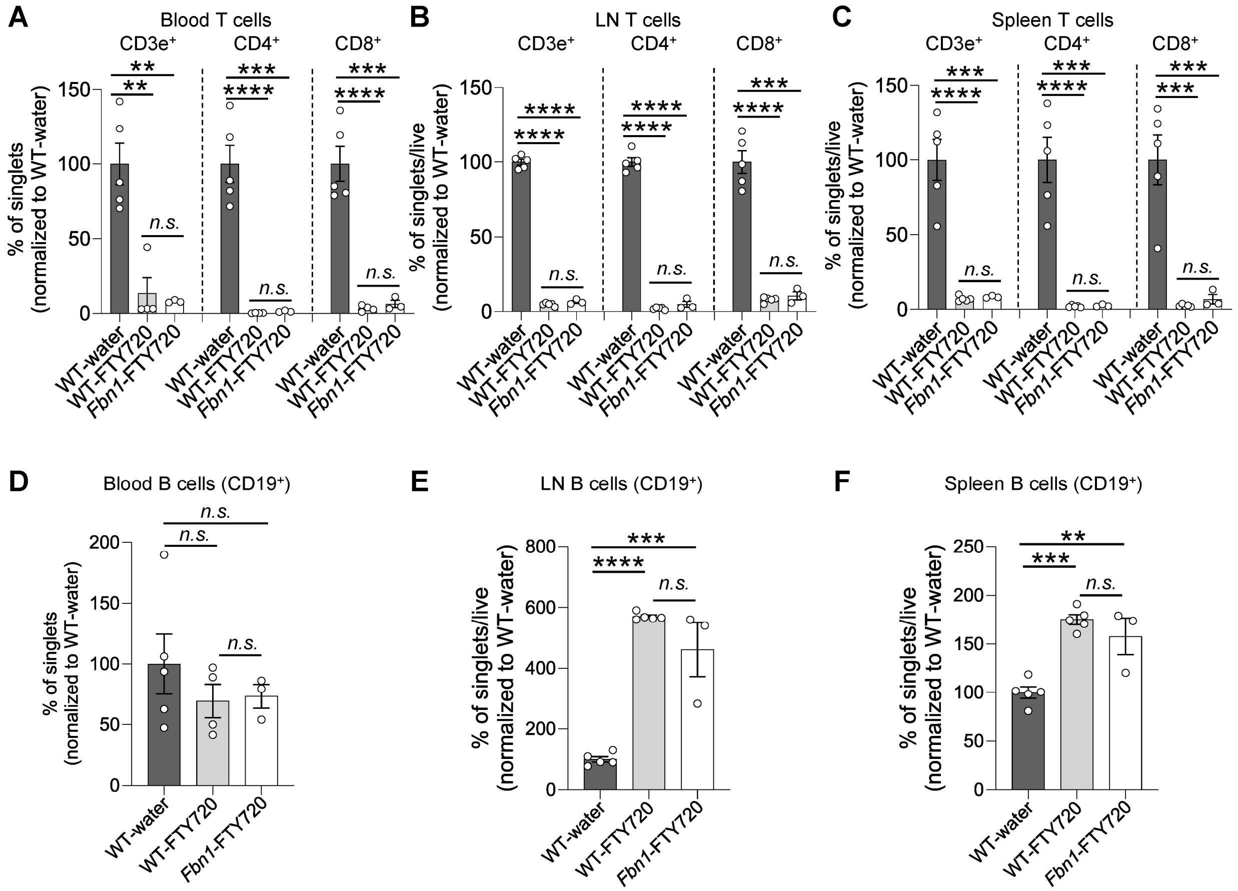

Supplemental Figure 18. Effect of FTY720 on mouse lymphocytes.
(A-F) Mice were treated with water or FTY720 (1mg/kg/day) from P1 to P15. At P16, blood, lymph nodes (LN), and spleens were collected, and T **(A-C)** and B **(D-F)** cell counts were quantified by flow cytometry. Data are Mean ± SEM, Ordinary one-way ANOVA test, each symbol represents one mouse, N=3~5, 2 males and 1~3 females. $^{**}P < 0.01$, $^{***}P < 0.001$, $^{****}P < 0.0001$, *n.s.*, not significant.

Supplemental Figure 19

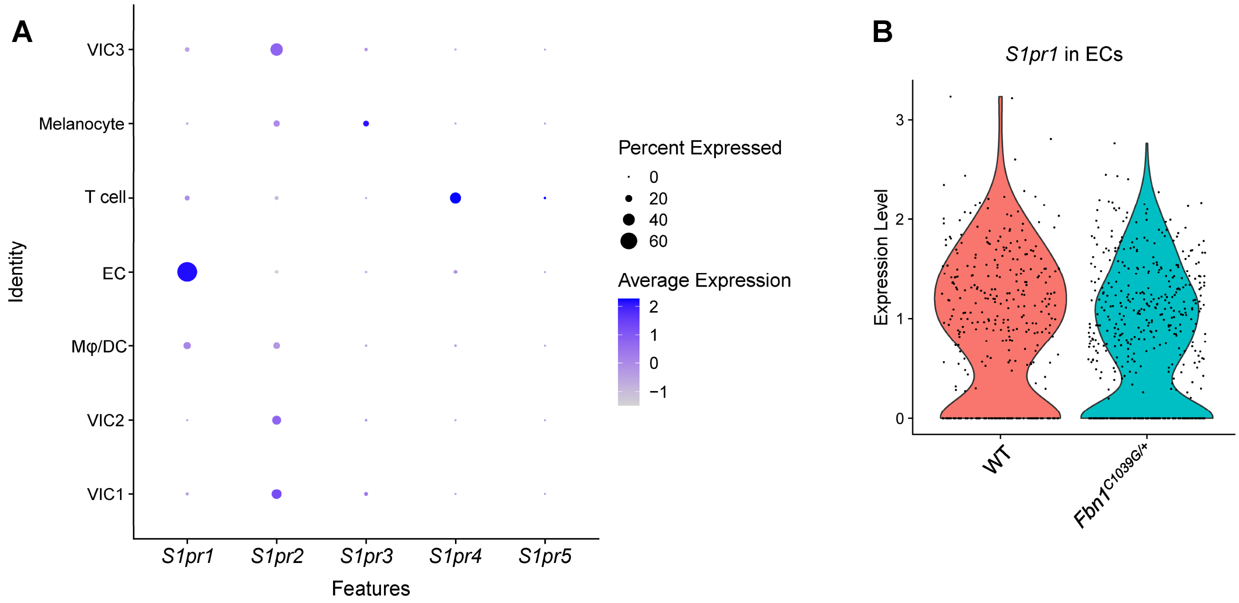

Supplemental Figure 19. Gene expression of S1P receptors in mouse MVs.
(A) The dot plot shows the expression of five S1P receptors across cell clusters from both WT and *Fbn1* mutant mouse MVs at P30, based on secondary analysis of the published scRNAseq data (GSE261874). **(B)** The violin plot shows the expression of *S1pr1* in ECs from WT and *Fbn1* mutant MVs. The difference is not significant.

Supplemental Figure 20

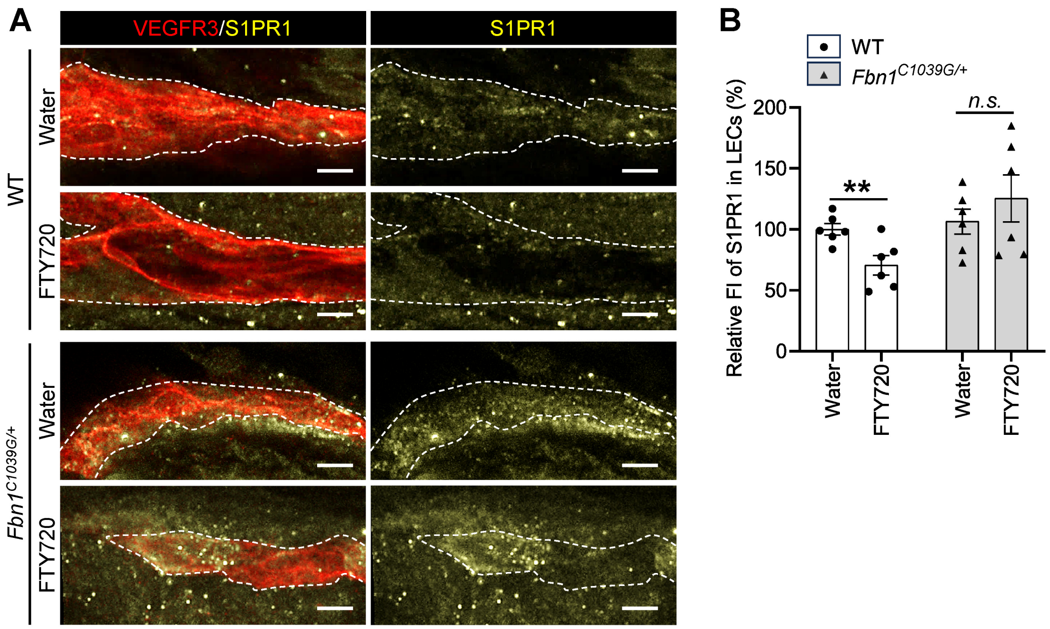

Supplemental Figure 20. FTY720 treatment suppresses S1PR1 in lymphatics of WT mitral valves.
(A) Representative confocal images of immunostaining in whole-mount MV show S1PR1 expression in lymphatics (outlined by dotted lines) of MVs at P60 after water/FTY720 treatment. Scale bars = 10 μm. **(B)** Quantification of S1PR1 fluorescent intensity (FI) in LECs of MVs. Data are Mean ± SEM, Student's unpaired *t*-test, each symbol represents one mouse, N = 6, male: female = 3:3 per group. ***P* < 0.01, *n.s.*, not significant.

Supplemental Figure 21

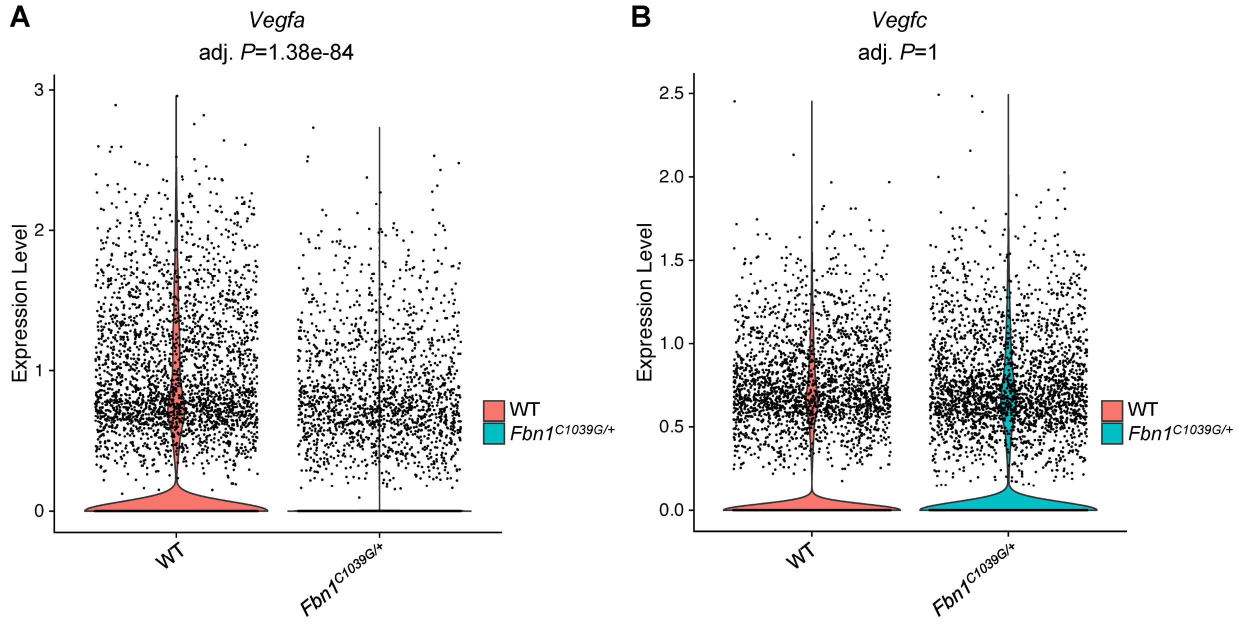

Supplemental Figure 21. Gene expression levels of *Vegfa* and *Vegfc* in MVs.
Dot plots show the expression levels of *Vegfa* **(A)** and *Vegfc* **(B)** in total MV cells from WT and *Fbn1* mutant mice at P30, based on secondary analysis of the published scRNAseq data (GSE261874).

Supplemental Figure 22

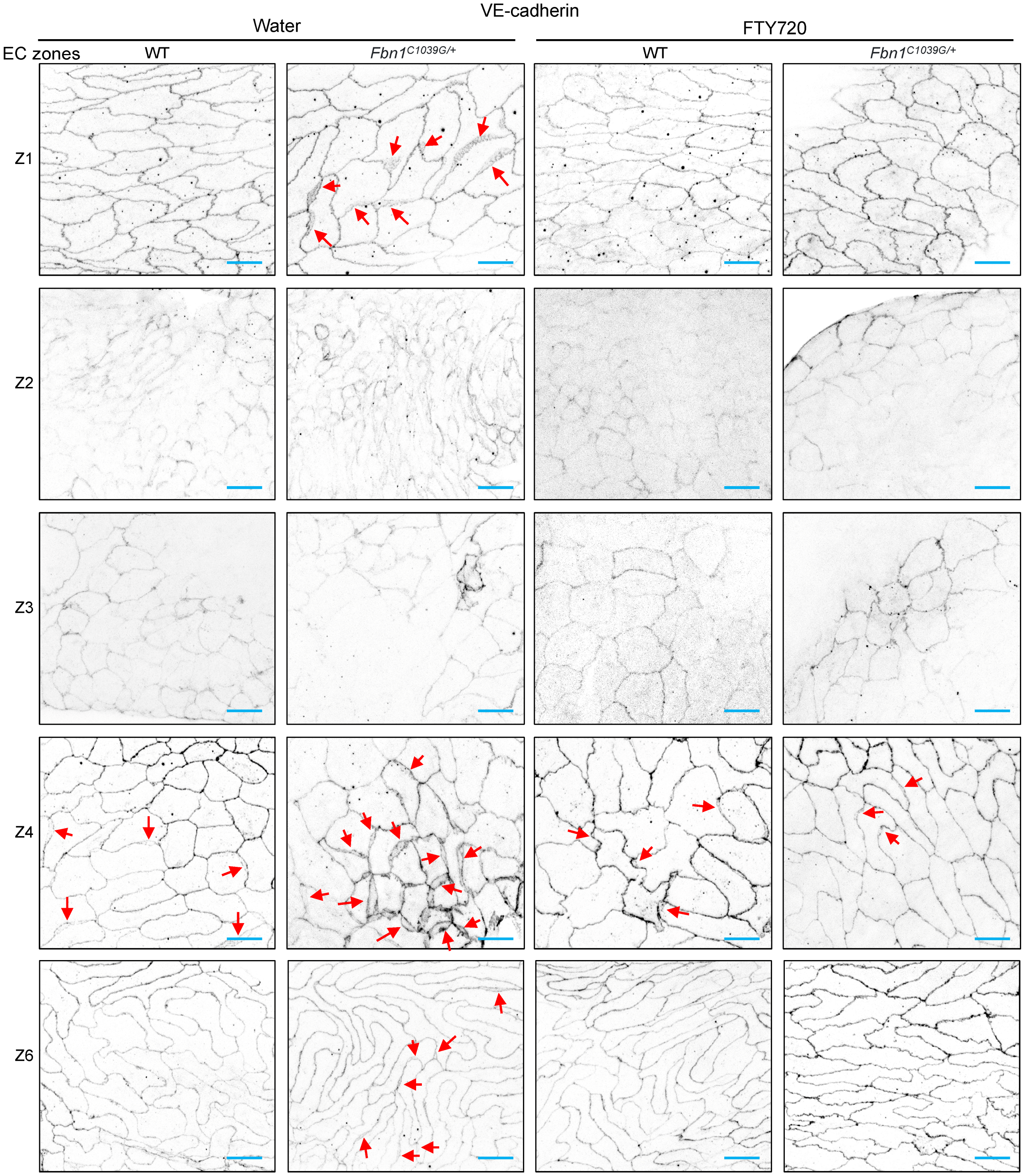

Supplemental Figure 22. FTY720 rescues disrupted cell junctions in VECs of *Fbn1* mutant mice. Representative confocal images of whole-mount MVs stained with VE-cadherin show the cell-cell junctions of VECs across different endothelial cell zones (1, 2, 3, 4, and 6). LEC junctions in EC zone 5 are shown in Figure 7D. Arrows indicate reticular adherens junctions. Scale bars = 20 μm.

Supplemental Figure 23

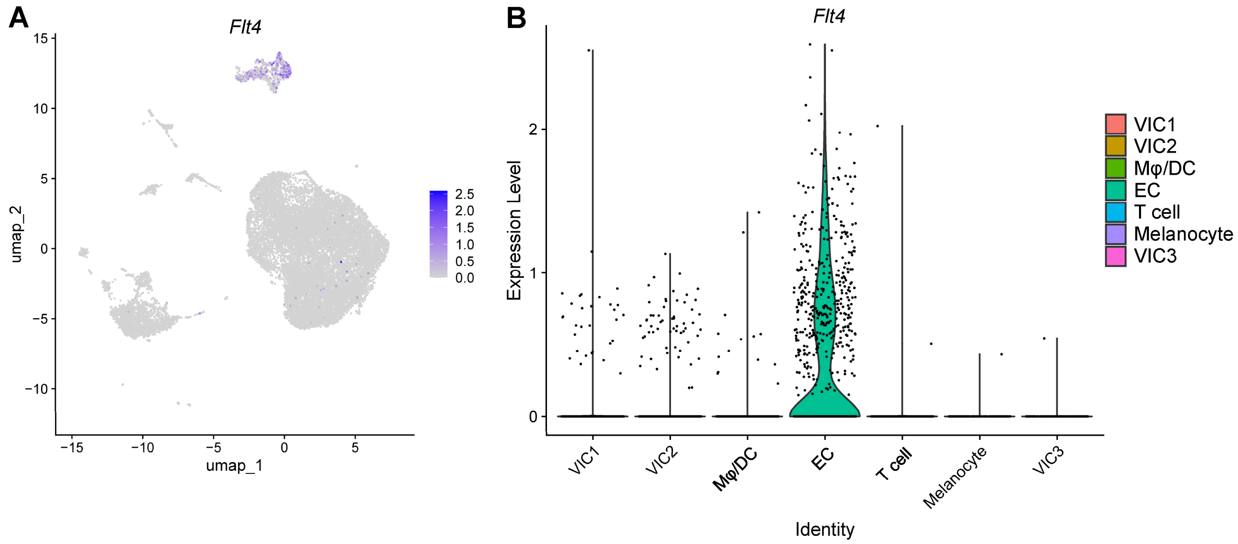

Supplemental Figure 23. Endothelial cells are the primary source of *Flt4* expression in the mitral valves. UMAPs **(A)** and violin plots **(B)** show the expression levels of *Flt4* (VEGFR3) across cell types in MVs of WT and *Fbn1* mutant mice at P30, based on secondary analysis of the published scRNAseq data (GSE261874).

Video Legends

Video 1

Lymphatic development in mitral valves. A representative 3-D reconstruction video shows the distribution of lymphatic vasculature in or near the mitral valve structure in a WT mouse at P14. This video was created using IMARIS software from the confocal image set of a whole-mount half-heart stained with VEGFR3 (green), as shown in Supplemental Figure 1D. The surface of the cardiac structures (white) was reconstructed, and the anatomy is shown as indicated in the video. Lymphatic vessels (arrows) from the anterior and posterior triangles, the dorsal wall of the left ventricle, and the right posterior side of the interventricular septum contribute to the lymphatics in the mitral annulus, where lymphatic vessels sprout and penetrate the mitral valve leaflets. IVS: interventricular septum; aL/pL: anterior/posterior leaflet. Scale bars are shown in the video. The display rate is 4 frames/second.

Video 2

Lymphatic development in the posterior triangle. A representative 3-D reconstruction video created using IMARIS software shows the distribution of lymphatic vasculature ($VEGFR3^+$, green) in the posterior triangle of the heart of a WT mouse at P14. The half-heart was opened to expose the posterior triangle after a cut through the anterior commissure and the left ventricular lateral wall, as shown in Supplemental Figure 1C. The surfaces of the cardiac structures (white or transparent) were constructed, and the anatomy was demonstrated in the video. Lymphatics in the posterior triangle originate from lymphatic vessels on the right side of the interventricular septum (IVS) and form

branches that penetrate the anterior and posterior mitral annulus. aL/pL: anterior/posterior leaflet. Scale bars are shown in the video. The display rate is 4 frames/second.

Supplemental Tables

Supplemental Table 1

Top 10 most significant DEGs in EC-3 cluster

Gene	p_val	avg_log2FC	pct.1	pct.2	p_val_adj
Zfp36	2.25337520661331e-06	-1.55631	0.566	0.844	0.044829
Elovl1	1.79559924801755e-05	1.365886	0.849	0.5	0.357217
Htra1	3.04180218961406e-05	-0.99921	0.868	0.938	0.605136
Gm47283	3.78089989905801e-05	1.632268	0.792	0.344	0.752172
Fst	4.62818964936023e-05	4.520777	0.604	0.219	0.920732
Hes1	5.16378216970845e-05	-1.39498	0.792	0.938	1
Ndufs4	5.43349594616715e-05	0.896923	0.962	0.719	1
Siva1	6.54679218402568e-05	1.820272	0.736	0.344	1
Nosip	8.3566834894534e-05	1.60825	0.698	0.25	1
1110038B12Rik	8.52540572817496e-05	1.24487	0.83	0.375	1

p-val: *P* value

avg_log2FC: Average Log2 Fold Change

pct.1: The percentage of cells in Group 1 (*Fbn1* mutant) that express the gene

pct.2: The percentage of cells in Group 2 (WT) that express the gene

p_val_adj: adjusted *P* value

Supplemental Table 2

Top 10 most significant DEGs in Mφ-0 cluster

Gene	p_val	avg_log2FC	pct.1	pct.2	p_val_adj
Junb	1.81788979920515e-121	-1.69482	0.852	0.964	3.61650996653872e-117
Nfkbia	2.11189918734224e-120	-2.00195	0.637	0.933	4.20141224329866e-116
Fos	4.03987189664437e-116	-1.69728	0.893	0.947	8.03692115118431e-112
Atf3	1.45108981056906e-114	-2.28936	0.515	0.896	2.88679806914608e-110
Egr1	2.25734055801733e-99	-2.33385	0.631	0.855	4.49075330611968e-95
Zfp36	1.86919901085251e-97	-1.47618	0.782	0.936	3.71858451218999e-93
Nfkbiz	1.40687575448811e-86	-1.93053	0.477	0.845	2.79883862597865e-82
Cxcl2	5.07635763496028e-86	-2.66555	0.393	0.782	1.009890587899e-81
Ppp1r15a	2.09181005689561e-79	-2.05313	0.246	0.705	4.16144692718813e-75
Ier3	3.10872117311434e-75	-2.43338	0.35	0.739	6.18448990179367e-71

p-val: *P* value

avg_log2FC: Average Log2 Fold Change

pct.1: The percentage of cells in Group 1 (*Fbn1* mutant) that express the gene

pct.2: The percentage of cells in Group 2 (WT) that express the gene

p_val_adj: adjusted *P* value

Supplemental Table 3

Top 10 most significant DEGs in Mφ-1 cluster

Gene	p_val	avg_log2FC	pct.1	pct.2	p_val_adj
Fos	3.91555798993753e-61	-1.62988	0.909	0.996	7.78961106518173e-57
Zfp36	1.06719676817577e-45	-1.46841	0.68	0.978	2.12308125060887e-41
Junb	2.86217510889978e-44	-1.19565	0.899	0.996	5.69401116164521e-40
Dusp1	8.75526110400215e-43	-1.2886	0.798	0.989	1.74177164403019e-38
Atf3	1.34615514789905e-42	-1.87967	0.498	0.932	2.67804105123038e-38
Fosb	7.32515727104186e-42	-1.74319	0.532	0.953	1.45726678750107e-37
Ppp1r15a	4.42207286633678e-40	-1.90897	0.411	0.863	8.7972717602904e-36
Gadd45b	9.78942878865293e-39	-2.27825	0.296	0.777	1.94750896321461e-34
Nfkbia	1.70253434794036e-36	-1.40491	0.724	0.964	3.38702183179254e-32
Btg2	7.05323763719077e-35	-1.29015	0.667	0.982	1.40317109554273e-30

p-val: *P* value

avg_log2FC: Average Log2 Fold Change

pct.1: The percentage of cells in Group 1 (*Fbn1* mutant) that express the gene

pct.2: The percentage of cells in Group 2 (WT) that express the gene

p_val_adj: adjusted *P* value

Supplemental Table 4

Top 10 most significant DEGs in DC cluster

Gene	p_val	avg_log2FC	pct.1	pct.2	p_val_adj
Cd83	7.18546731131997e-10	-1.53381	0.483	0.941	1.42947686691399e-05
H2-Eb1	1.59650718991841e-08	0.61808	1	0.985	0.000318
Pim1	2.02341174328937e-08	-1.30624	0.707	0.941	0.000403
Txnrd1	2.28026526513178e-07	-3.16258	0.069	0.5	0.004536
Csrnp1	2.57406711328046e-07	-2.32057	0.155	0.603	0.005121
Nfkbid	3.24230337163813e-07	-2.93481	0.086	0.515	0.00645
Junb	9.72555730527768e-07	-0.73312	0.966	0.985	0.019348
D930030I03Rik	1.41956004126263e-06	-3.2551	0.034	0.397	0.028241
Cd74	1.98311023807168e-06	0.47966	1	1	0.039452
Zfp36	2.02314630107744e-06	-0.96829	0.914	0.941	0.040248

p-val: *P* value

avg_log2FC: Average Log2 Fold Change

pct.1: The percentage of cells in Group 1 (*Fbn1* mutant) that express the gene

pct.2: The percentage of cells in Group 2 (WT) that express the gene

p_val_adj: adjusted *P* value

Supplemental Tabel 5

Antibodies used for WM and IHC staining

Target antigen	Vendor	Catalog #	Application
CCR7	Abcam	ab253187	IHC-F
CD206	R&D	AF2535SP	WM, IHC-P
CD31	BD Biosciences	553370	WM, IHC-F
CD31	Abcam	ab222783	WM, IHC-P
CD31	R&D	AF3628	WM, IHC-P
CD45	R&D	AF114	IHC-F, IHC-P
Collagen I	SouthernBiotech	1310-01	IHC-F
HABP-biotinylated	Sigma	385911	WM, IHC-F
LYVE1	Abcam	ab14917	WM
MHCII	eBioscience	14-5321-82	WM
p-Akt	Proteintech	28731-1-AP	WM
p-ERK1/2	Cell Signaling	4370	WM
PROX1	Abcam	ab199359	WM
p-SMAD2	Cell Signaling	3108T	WM
p-VEGFR3	Affinity Biosciences	AF3676	WM
S1PR1	Invitrogen	PA1-1040	WM
VE-cadherin	BD	555289	WM
VEGFR3	R&D	AF743	WM
Donkey anti-Goat IgG-488	Invitrogen	A-11055	WM, IHC-P
Donkey anti-Goat IgG-568	Invitrogen	A-11057	WM, IHC-P
Donkey anti-Goat IgG-647	Invitrogen	A-21447	WM, IHC-F
Donkey anti-Rabbit IgG-488	Invitrogen	A-21206	WM, IHC-P
Donkey anti-Rabbit IgG-568	Invitrogen	A10042	IHC-F
Donkey anti-Rabbit IgG-647	Invitrogen	A-31573	WM
Donkey anti-Rat IgG-488	Invitrogen	A-21208	WM, IHC-F
Donkey anti-Rat IgG-568	Abcam	ab175475	WM
Donkey anti-Rat IgG-647	Invitrogen	A48272	WM
Streptavidin-Alexa 488	Jackson ImmunoResearch Labs	016-540-084	WM, IHC-F

WM: Whole-mount immunostaining.

IHC-F: Immunohistochemistry staining on frozen sections.

IHC-P: Immunohistochemistry staining on paraffin sections.

Supplemental Table 6

Antibodies used for flow cytometry

Fluorophores & antibody	Vendor	Catalog #	Clone #
Alexa Fluor® 700 Hamster anti-mouse CD11c	BD Biosciences	#560583	HL3
APC Rat anti-mouse CD206	Biolegend	#141707	C068C2
APC-Cy™7 Rat anti-mouse CD45	BD Biosciences	#557659	30-F11
BUV395 Rat anti-mouse CD192 (CCR2)	BD Biosciences	#747972	475301
BUV395 Rat Anti-Mouse I-A/I-E	BD Biosciences	#569244	2G9
BUV395 Rat Anti-Mouse CD4	BD Biosciences	#563790	GK1.5 (RUO)
BUV737 Rat anti-mouse CD11b	BD Biosciences	#612800	M1/70
BV421 Rat Anti-Mouse CD169	BD Biosciences	#566604	3D6/CD169
eFluor™ 450 Rat anti-mouse Ly6C	Invitrogen/ThermoFisher Scientific	#48-5932-82	HK1.4
FITC Hamster anti-mouse CD3e	Invitrogen/ThermoFisher Scientific	#11-0031-82	145-2C11
FITC Rat anti-mouse CD8a	Biolegend	#100706	53-6.7
FITC Rat anti-mouse I-A/I-E	Invitrogen/ThermoFisher Scientific	#11-5321-82	M5/114.15.2
PerCP/Cyanine5.5 Mouse anti-mouse CD64	Biolegend	#139307	X54-5/7.1
PE-CF594 Rat anti-mouse CD19	BD Biosciences	#562291	1D3
PE-CF594 Rat anti-mouse Siglec-F	BD Biosciences	#562757	E50-2440
PE-Cy™7 Rat Anti-Mouse Ly-6G	BD Biosciences	#560601	1A8
PE-Cy™7 Mouse Anti-Mouse NK1.1	Invitrogen/ThermoFisher Scientific	#A15418	PK136
PE Hamster Anti-Mouse CD3e	BD Biosciences	#553064	145-2C11 (RUO)
PE anti-mouse F4/80	Biolegend	#123110	BM8
