# Supplementary material for: Lymphatic dysfunction and ZFP36 deficiency contribute to myxomatous valve degeneration in Marfan syndrome mice
Source: J Clin Invest. 2026 Jun 2;136(15):e195507. doi: 10.1172/JCI195507 (PMC13430018; doi:10.1172/JCI195507)
Supplement: Unedited blot and gel images [file jci-136-195507-s036.pdf]

Uncut blot images for Supplemental Figure 16A

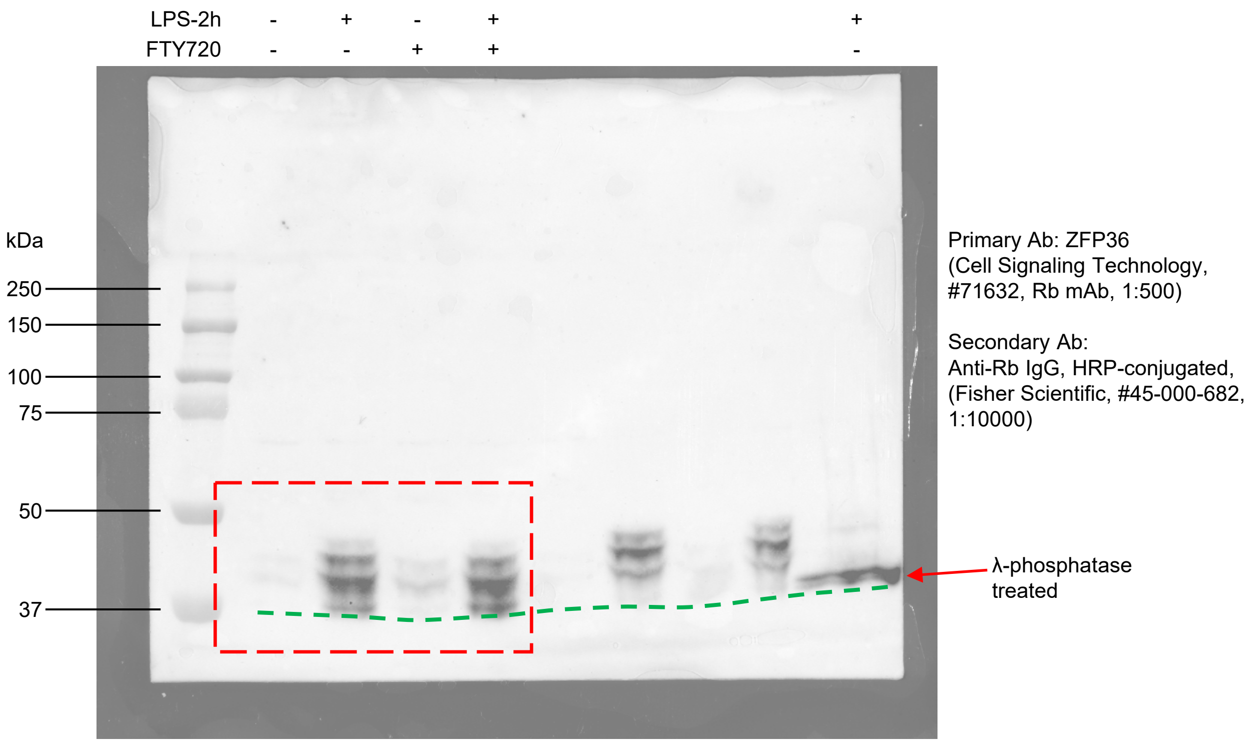

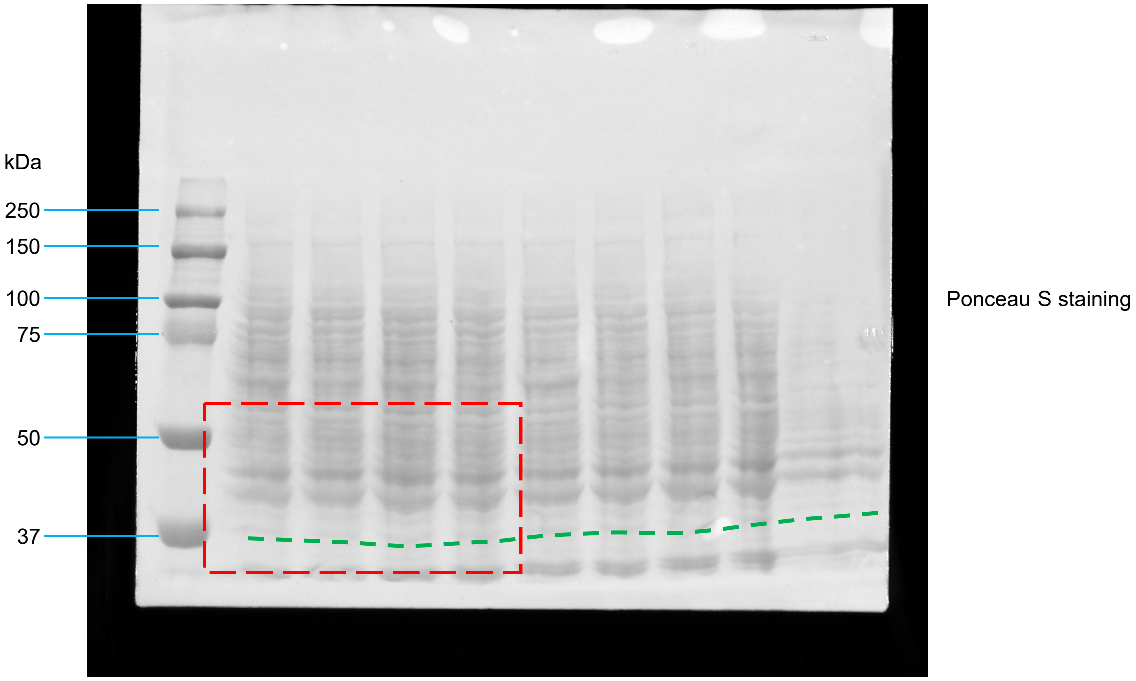

Uncut blot images. Red boxed regions are used for representative images. Lambda Protein Phosphatase (λ-phosphatase)-treated control lysate sample (red arrow) was used as a reference to determine the unphosphorylated form of ZFP36. Green dotted lines indicate bands of the same molecular weight across all lanes.
